# The cost of inaction on physical inactivity to public health-care systems: a population-attributable fraction analysis

**DOI:** 10.1016/S2214-109X(22)00464-8

**Published:** 2022-12-05

**Authors:** Andreia Costa Santos, Juana Willumsen, Filip Meheus, Andre Ilbawi, Fiona C Bull

**Affiliations:** a WHO, Geneva, Switzerland

## Abstract

**Background:**

Physical inactivity is an important modifiable risk factor for non-communicable diseases (NCDs) and mental health conditions. We aimed to estimate the public health-care costs associated with these diseases because of physical inactivity, which will help policy makers to prioritise investment in policy actions to promote and enable more people to be more active.

**Methods:**

We used a population-attributable fraction formula to estimate the direct public health-care costs of NCDs and mental health conditions for 2020–30. The disease outcomes that we included were incident cases of coronary heart disease, stroke, type 2 diabetes, hypertension, cancer (breast, colon, bladder, endometrial, oesophageal, gastric, and renal), dementia, and depression in adults aged at least 18 years. We used the most recent health and economic data evidence available for 194 countries.

**Findings:**

499·2 million new cases of preventable major NCDs would occur globally by 2030 if the prevalence of physical inactivity does not change, with direct health-care costs of INT$520 billion. The global cost of inaction on physical inactivity would reach approximately $47·6 billion per year. Although 74% of new cases of NCDs would occur in low-income and middle-countries, high-income countries would bear a larger proportion (63%) of the economic costs. The cost of treatment and management of NCDs varied—although dementia accounted for only 3% of new preventable NCDs, the disease corresponded to 22% of all costs; type 2 diabetes accounted for 2% of new preventable cases but 9% of all costs; and cancers accounted for 1% of new preventable cases but 15% of all costs.

**Interpretation:**

This health and economic burden of physical inactivity is avoidable. Further investments in and implementation of known and effective policy interventions will support countries to reach the Sustainable Development Goal of reduction of NCD mortality by 2030.

**Funding:**

None.

## Introduction

Most countries are falling behind on their commitments to the 2030 UN Sustainable Development Goal (SDG) 3.4 to reduce by a third the premature mortality from non-communicable diseases (NCDs), the leading cause of death and ill health globally.^1^ Also of concern is the increasing global burden of mental health problems, exacerbated by the COVID-19 pandemic.^2^ At this pace, countries are unlikely to achieve their 2030 SDG 3 commitments of ensuring healthy lives and promoting wellbeing for all at all ages.^1^

Reducing the prevalence of modifiable risk factors, such as tobacco use, harmful use of alcohol, unhealthy diets, and physical inactivity, is a cost-effective strategy to reduce the burden of NCDs and mental health problems. Every US$1 invested in scaling up effective interventions to reduce risk factors and manage NCDs, for example, could generate a return of up to US$7 in low-income and middle-income countries (LMICs), where almost 85% of all premature deaths due to NCDs occur every year.^3^ Yet, slow progress has been observed over the years, especially in those settings.

Physical inactivity is a major modifiable risk factor for NCDs and mental health conditions including stroke, hypertension, type 2 diabetes, coronary heart disease, several types of cancers, dementia, depression, and all-cause mortality; in particular, deaths due to cardiovascular diseases.^4^ The global costs of physical inactivity to health-care systems, based on only five health outcomes (coronary heart disease, stroke, type 2 diabetes mellitus, breast cancer, and colon cancer), were estimated at INT$53·8 billion (2013), of which 58% was paid by the public sector.^5^

To support countries' responses, WHO identified 20 evidence-based policy recommendations, outlined in the WHO Global Action Plan on Physical Activity 2018–30, to guide national efforts to increase population levels of physical activity.^6^ But global progress on reducing levels of physical inactivity has been slow.^6^ Stronger advocacy is needed to establish the multisectoral action necessary to promote and enable more physical activity. This advocacy can be supported with an economic case for governments and non-governmental organisations to invest in physical activity.^7^

**Research in context**

**Evidence before this study**

Physical inactivity increases the risk of death from non-communicable diseases (NCDs). Only one previous global study has estimated the direct health-care costs resulting from physical inactivity using a disease prevalence-based approach, reporting an economic cost to society of INT$53·8 billion (2013 prices), of which 58% was paid by the public sector. That study included five health outcomes for which estimates of the relative risks were available at the time. However, the study did not address the important questions of what would be the current and future potential preventable public health-care costs that could be averted if levels of physical inactivity were to be reduced or eliminated.

**Added value of this study**

This is the first global study to provide estimates of the number of new cases and associated public health-care costs that would occur from 2020 to 2030 that could be prevented if levels of physical inactivity were reduced or eliminated. This study provides new population-attributable fractions for seven health outcomes and mental health conditions (coronary heart disease, stroke, type 2 diabetes, cancers [breast, colon, bladder, endometrial, gastric, oesophageal, and renal], depression, and dementia) with strong evidence for the association with physical inactivity.

**Implications of all the available evidence**

This study combines the assessment of the health and economic impacts of NCDs and mental health conditions associated with physical inactivity, and provides policy makers with empirical data on the cost of not acting to reduce physical inactivity. These data will equip policy makers with evidence to inform and advocate for greater investment in policy interventions that increase physical activity levels. This study calls for urgent action by countries to prioritise investments in interventions that reduce this modifiable risk factor. WHO's Global Action Plan on Physical Activity provides clear guidance on evidence-based policy recommendations, which if implemented by countries will improve health, reduce the burden on health systems, and save money.

Making the investment case for physical activity is key to informing decision making and prioritising resources and generating political and societal support for policy implementation. Estimating the health and economic costs of continuing with no action to reduce levels of physical inactivity is the first step in building a case for investment in physical activity.

We aimed to estimate the cost to public health-care systems of inaction on physical inactivity. This study is the first global estimate of the number of new (incident) cases of disease and their associated public health-care costs that would occur from 2020 to 2030 and to present the costs that could potentially be averted if levels of physical inactivity were reduced or eliminated.

## Methods

### Study design

We assessed the total direct costs to the public health system incurred by new cases (ie, using an incidence-based approach) of seven diseases strongly associated with physical inactivity to estimate the cost of inaction on physical inactivity. The disease outcomes that we included were incident cases of coronary heart disease, stroke, type 2 diabetes, hypertension, cancer (breast, colon, bladder, endometrial, oesophageal, gastric, and renal), dementia, and depression, which we selected because these diseases were identified in the latest WHO Guidelines on Physical Activity and Sedentary Behaviour.^8^ We provide a conceptual framework, the rationale for using an incidence approach, and the methodological steps for the economic assessment in the appendix (pp 2–4).

Estimates of the economic costs of physical inactivity are presented by country, WHO region, and the World Bank income-level classification. All costs are provided in 2020 market exchange prices (US$) and INT$ using 2020 purchasing power parity conversion factors. INT$ refers to the amount of goods and services that an individual (or government) would buy in their respective country compared with what individuals (or the government) of the USA would buy in the country.^9^ Using INT$ provides an international comparison by having the US$ as the currency of reference.^9^

### Attributing incident cases to physical inactivity

We used the most recent global comparable national estimates for the prevalence of physical inactivity from WHO, for adults aged 18 years old and older, and by sex.^10^, ^11^ Physical inactivity was defined as not meeting the WHO physical activity recommendations: for adults, at least 150 min of moderate-intensity aerobic physical activity or at least 75 min of vigorous-intensity aerobic physical activity or an equivalent combination of moderate-intensity and vigorous-intensity activity per week.^8^ This definition excludes the muscle strengthening component of the WHO guidelines^8^ but aligns with the available measures of physical inactivity at the population level that are available.^4^

We also used published estimates of the multivariable-adjusted relative risks (RRs) for each of the seven diseases outcomes.^4^ The RRs were derived from the most recent meta-analysis or pooled data of systematic literature reviews that provided strong evidence of association with the outcomes of interest. These adjusted RR estimates (controlled by confounders) are based on an assessment of the precision of the effect, risk of bias, consistency of results from different studies, and directness of the evidence.^4^ NCD and mental health outcomes with moderate, weak, or insufficient evidence for associations with physical inactivity were not included in this analysis. We excluded, for example, lung, head, and neck cancers, and attention-deficit hyperactivity disorder (appendix p 9).^6^, ^8^

We used these data to estimate the population-attributable fraction (PAF) of incident cases due to physical inactivity using the following formula, where *p*_i_ is the prevalence of physical inactivity.^12^

$$semi-adjustedPAF=\frac{p_{i}(adjustedRR-1)}{[p_{i}(adjustedRR-1)]+1}$$

We used the semi-adjusted PAF formula.^12^ According to Rockhill and colleagues,^12^ the semi-adjusted PAF should be used with unadjusted RRs to avoid bias in the analysis. However, given the data availability and the worldwide scope of our analysis, we opted for the use of multivariable-adjusted RRs with the semi-adjusted formula. Wong and colleagues^13^ assessed with simulations the effect of bias in the semi-adjusted PAF and concluded that the semi-adjusted PAF fell within the interval for low bias (–10% to +10%) almost always when multivariable-adjusted RRs were used.^13^

We interpreted the PAF as the proportion of disease risk in the total population that could be reduced or eliminated, to keep both the theoretical interpretation of the PAF and to call attention to the unrealistic expectation of total elimination of all risks.^14^

We obtained annual incidence for each outcome from various sources. Total incident cases by type of cancer were obtained from the WHO International Agency for Research on Cancer, which provides estimates for 2020.^15^ For type 2 diabetes, depression, coronary heart disease, and stroke, data were obtained on incident cases from the 2019 Global Burden of Diseases, Injuries, and Risk Factors Study (GBD).^16^ Depression and anxiety were considered together in this analysis because these conditions are difficult to disaggregate for cost assessments. Incidence rates per 1000 population were obtained from Alzheimer's Disease International to estimate the incident cases for dementia.^17^ We used 2014 WHO data on crude prevalence for high blood pressure, defined as the percentage of the defined population with high blood pressure (systolic blood pressure ≥140 mm Hg or diastolic blood pressure ≥90 mm Hg), as a proxy for the number of incident cases of hypertension.^18^

We used the most recent number of incident cases by disease as baseline (using 2020 as the reference year), and estimated the number of new cases, for each year, from 2020 to 2030. When these data were only available before 2020, we used UN data on total population^19^ and applied population growth rates to the number of incident cases from the year of data availability until 2020. For example, data on incidence of depression, coronary heart disease, stroke, and type 2 diabetes were obtained from the GBD study for 2019, the most recent estimates available, and we applied 1-year average UN population growth rate, by country, to bring these estimates to 2020.

We estimated the total number of new cases by multiplying the PAFs by the total number of incident cases of diseases in the population, per year, from 2020 to 2030, and using population growth rates (appendix p 4).

### Attributing direct medical costs to physical inactivity

We adopted the perspective of the public health sector and excluded private sector and household costs, as well as societal costs with productivity losses and death, because of methodological challenges in assessing these costs.^20^

We collated total direct costs to the public health system that were attributed to physical inactivity for each health condition from multiple sources. For cancer outcomes, we obtained country-specific costs of incident cases of cancers from the WHO Cancer Unit (unpublished). These costs were based on a cohort of new cases of cancers for which the cost of diagnosing and treating these cancers were identified and quantified from the perspective of the public health system, using an ingredient-based approach in which the use of each input (eg, medical and non-medical time, medication, tests, and overheads) was estimated—from diagnosis throughout the first year of treatment of the disease—and inputted as a price (appendix p 24).

For type 2 diabetes, we used the most recent estimates from the International Diabetes Federation (IDF).^21^ Costs estimates by the IDF include costs for complications of type 2 diabetes associated with coronary heart disease and stroke. To avoid double counting the costs of stroke and coronary heart disease in patients with type 2 diabetes, we deducted the direct health-care costs per case of (1) type 2 diabetes and stroke; (2) type 2 diabetes and coronary heart disease; and (3) type 2 diabetes, coronary heart disease, and stroke from the total estimates of costs attributed to physical inactivity from the total global costs (appendix p 79).

We obtained costs to treat dementia from a WHO publication on public health response to dementia.^22^ These costs, however, were only available, and were thus presented, by WHO regions and World Bank country-income levels, and not by country (appendix p 5).

For stroke, hypertension, coronary heart disease, and depression, no global datasets on the direct health-care costs were available, thus we adopted the same approach used by Ding and colleagues, the Economist Intelligence Unit, and the World Economic Forum.^5^ Following this approach, we obtained data on the national disease-specific health-care costs from OECD for 28 European Union countries (EU28) at the time, and for hypertension, from the National Health Service (NHS) England,^23^ and extrapolated these costs to other countries by using a country weighting factor. The country weighting factor was constructed as the country health expenditure for a specific year divided by the mean of health-care expenditure for the EU28 (and NHS England for hypertension) for the same year, at purchasing power parity. This weighting factor was then applied to the average health-care costs, per type of disease, for the EU28's and NHS England's costs. The health-care expenditure per country was obtained from the WHO Global Health Expenditure Database for the public sector.^24^ Costs estimates on stroke, coronary heart disease, and depression are given in the appendix (p 30).

For each disease, we calculated the total annual direct costs to the health system per country by multiplying the estimated total number of new cases of diseases attributed to physical inactivity by the estimated mean annual costs per disease; annual costs in subsequent years for the treatment of prevalent cases were not included in the analysis. Total annual costs were extrapolated from 2020 to 2030 by using country-specific inflation rates as informed by the World Bank.^25^

### Sensitivity analysis

We presented all estimates as the mean, lower, and upper bounds of the CIs for the RRs. Because cost estimates were derived from European countries and applied to all other countries, uncertainty around the true costs to LMICs were accounted for by applying a reduction in costs of 30% and 50% to reflect the potential variation in costs across LMICs (appendix p 6).

## Results

**Figure 1:**
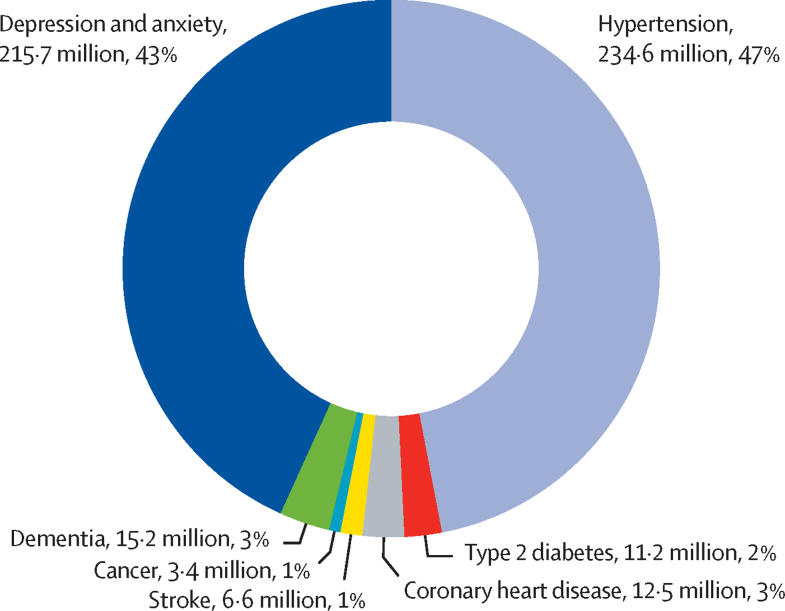
Total global number and proportion of new cases of non-communicable diseases and mental health conditions attributed to physical inactivity, 2020–30

**Figure 2:**
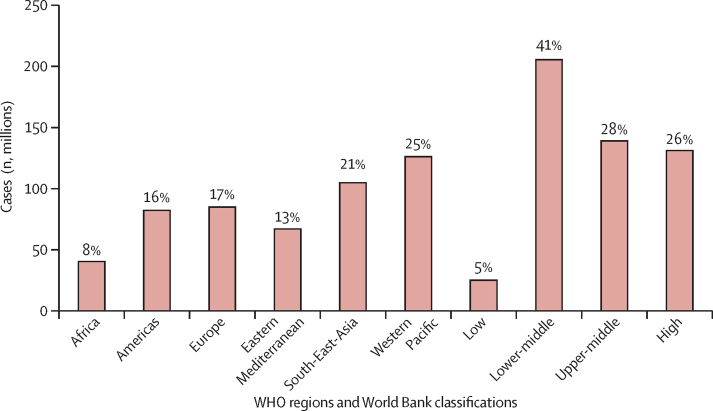
Number and proportion of new cases of non-communicable diseases and mental health conditions attributed to physical inactivity by WHO region and World Bank country-income level, 2020–30

We estimated that 499·2 million new cases of preventable NCDs and mental health conditions would occur globally, from 2020 to 2030, if the current prevalence of physical inactivity does not change. 234·6 million (47%) of these new cases would be hypertension and 215·7 million (43%) would be due to depression and anxiety (figure 1). 368·4 million (74%) new cases would occur in LMICs, and of the total number of new cases of NCDs and mental health conditions due to physical inactivity 125·9 million (25%) would occur in the Western Pacific region and 103·5 million (21%) in the South-East Asia region (figure 2). Results of the analyses by country and WHO region and World Bank income classification are given in the appendix (pp 35, 49).

**Figure 3:**
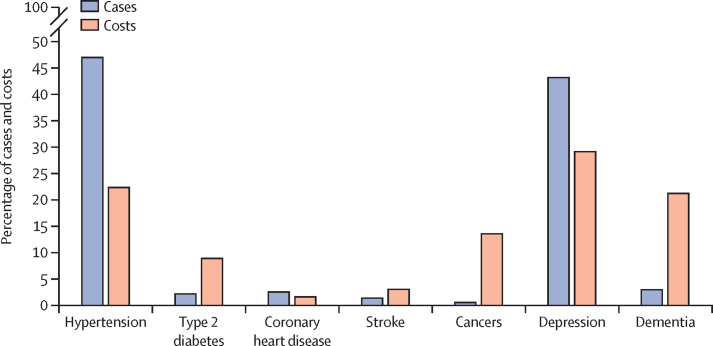
Total global proportion of new cases and direct health-care costs of non-communicable diseases and mental health conditions attributed to physical inactivity, 2020–30

We estimated the global cost of all preventable NCDs and mental health conditions to reach INT$523·9 billion (US$301·8 billion) for the period 2020–30, approximately INT$47·6 billion (US$27·4 billion) per year (table; appendix pp 51–75). The costs of treatment and management of NCDs and mental health conditions varied such that although dementia accounted for only 3% of preventable new cases, the disease accounted for 22% of total direct health-care costs (figure 3). Furthermore, type 2 diabetes accounted for 2% of preventable cases but 9% of all costs, and cancer accounted for 1% of all cases but 15% of all costs (figure 3).

**Table:** Direct public health-care costs (in 1 000 000 INT$) attributable to physical inactivity, 2020–30

	**Cancers**	**Coronary heart disease**	**Dementia**	**Depression**	**Hypertension**	**Stroke**	**Type 2 diabetes**	**Total**
**WHO region**								
African region	375 (80–755)	141 (98–191)	389 (203–642)	6598 (247–13 781)	3128 (1573–4667)	314 (152–456)	1151 (753–1541)	12 098 (3106–22 032)
Region of the Americas	9465 (1899–18 323)	1400 (977–1873)	55 560 (29 804–88 239)	37 536 (1466–75 007)	21 018 (10 618–31 207)	2601 (1274–3723)	32 479 (21 439–43 090)	160 058 (67 478–261 464)
Eastern Mediterranean region	6969 (1131–14 273)	2733 (1912–3647)	862 (459–1382)	42 283 (1670–83 567)	21 546 (10 899–31 951)	3066 (1507–4374)	3847 (2546–5092)	81 307 (20 123–144 286)
European region	30 325 (5804–60 120)	4696 (3268–6304)	27 889 (14 719–45 236)	70 224 (2706–142 348)	51 111 (25 779–76 008)	6168 (3005–8867)	10 269 (6758–13 664)	200 682 (62 040–352 546)
South-East Asia region	1633 (305–3351)	1166 (808–1572)	10 441 (5521–16 895)	18 668 (706–38 605)	15 360 (7731–22 890)	1663 (805–2405)	1622 (1063–2167)	50 554 (16 938–87 885)
Western Pacific region	19 536 (3035–40 141)	1913 (1332–2567)	24 382 (12 628–40 562)	30 852 (1192–62 357)	29 387 (14 827–43 688)	6991 (3410–10 041)	10 207 (6722–13 574)	123 269 (43 146–212 930)
**World Bank country-income level**								
Low	136 (24–290)	33 (23–45)	140 (72–234)	1606 (60–3377)	759 (381–1133)	79 (38–115)	125 (81 to 167)	2878 (679–5361)
Lower-middle	2338 (401–4850)	1619 (1127–2172)	6238 (3295–10 104)	28 873 (1114–58 434)	19 244 (9708–28 613)	2297 (1120–3301)	3944 (2596 to 5246)	64 553 (19 362–112 720)
Upper-middle	8033 (1291–16 843)	2435 (1688–3283)	24 732 (12 896–40 767)	41 517 (1572–85 784)	30 656 (15 430–45 680)	6511 (3152–9413)	14 489 (9498 to 19 352)	128 372 (45 527–221 122)
High	60 730 (11 497–119 174)	4538 (3171–6064)	80 067 (42 778–127 816)	80 755 (3170–160 598)	66 535 (33 631–98 736)	7227 (3545–10 331)	28 255 (18 671 to 37 450)	328 108 (116 462–560 168)
Total by health outcomes and income level	71 238 (13 212–14 1156)	8626 (6008–11 565)	11 1176 (59 042–178 921)	152 751 (5916–30 8193)	117 193 (59 150–174 162)	16 114 (7855–23 160)	46 813 (30 847 to 62 215)	523 911 (182 030–899 371)

Costs are INT$ (95% CI).

*Excludes double counting (appendix p 79).

**Figure 4:**
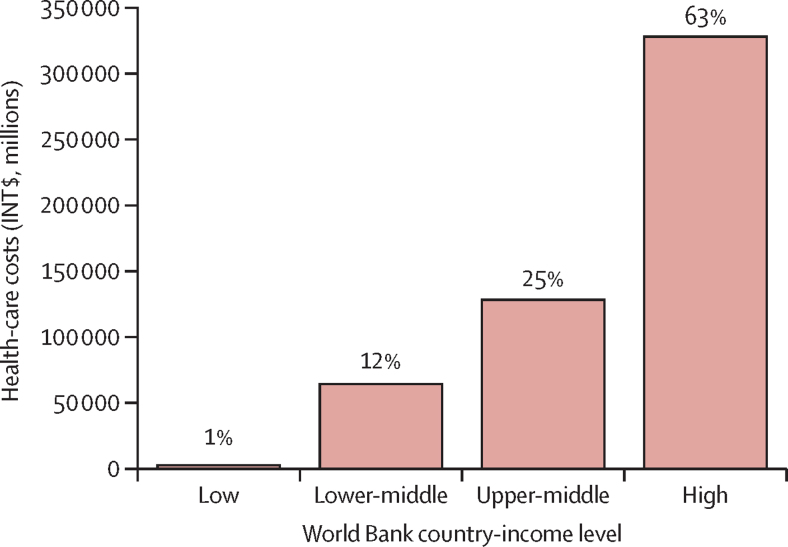
Costs and proportions of direct health-care costs of new cases of non-communicable diseases and mental health conditions attributed to physical inactivity by World Bank country-income level, 2020–30

**Figure 5:**
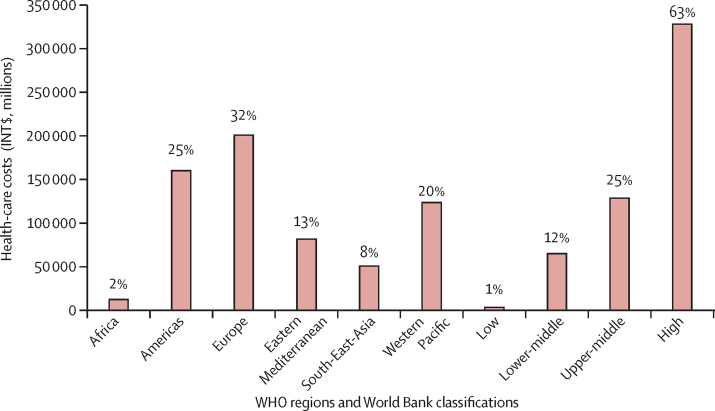
Total direct health-care costs and proportion of costs of new cases of non-communicable diseases and mental health conditions attributed to physical inactivity by WHO region and World Bank country-income level, 2020–30

Although most (74%) new cases would occur in LMICs (figure 2), high-income countries would bear the largest proportion of economic costs (63%; figure 4). The economic burden attributable to physical inactivity was highest in the European region (32%), followed by the region of the Americas (25%), Western Pacific region (20%), Eastern Mediterranean region (13%), South-East Asia region (8%), and African region (2%; figure 5).

New cases of preventable diseases would vary from 154 million to 860 million cases, when assessing the lower and upper bounds of RRs, with costs varying from INT$182 billion to INT$900 billion for these RRs (table). When dealing with uncertainties around costs estimates, a variation of –50% would reduce the cost of inaction to INT$344 billion (US$164 billion) and a variation of –30% by INT$499 billion (US$219 billion; appendix p 77).

## Discussion

We estimated that the total cost of physical inactivity globally would be approximately INT$520 billion over an 11-year period (2020–30) if global levels of physical activity are not increased. Of particular concern is the high burden of physical inactivity seen in preventable cases of dementia and cancers because, despite the relatively lower incidence of these conditions compared with other NCDs, these diseases incur a high cost because of requirements of diagnosis, treatment, and long-term management. Furthermore, although most predicted new cases of NCDs would occur in LMICs, high-income countries will bear a larger proportion of the economic burden. This finding reflects the increased coverage and cost of health care in wealthy countries compared with lower-income settings. Our findings also highlight the high number of cases of depression and anxiety, rates of which steadily increased during the COVID-19 pandemic.^2^, ^26^ Incidence and prevalence of those mental health disorders can also be effectively reduced by increasing physical activity levels, which would also help to reduce costs to health-care systems and increase wellbeing.^6^

This is the first study to assess the global cost of inaction to the public health-care system of new (incident) cases of NCD and mental health diseases due to physical inactivity that could be prevented; therefore, the comparison with previous studies is limited. One prominent study by Ding and colleagues^5^ used a prevalence approach, whereby new and existing cases were included in the cost estimate associated with physical inactivity.^5^ Although Ding and colleagues' assessment gives policy makers the economic value associated with the treatment and management of NCD cases, their assessment does not provide an estimate of the potential costs averted (ie, savings) if physical inactivity were reduced or eliminated, because most chronic diseases cannot be averted once they exist (appendix p 3). Thus, their estimate of INT$31·2 billion (in 2013 prices) in direct health-care costs to the public health system would be higher than our reported INT$47·6 billion (in 2020 prices) if replicated today and conducted with the same seven health outcomes studied here; however, direct comparison of costs between these studies is not appropriate because of important methodological differences.

One other global study reported the potential global economic benefits of a reduction in physical inactivity levels in terms of productivity gains and deaths averted.^27^ The authors report costs of between US$314 billion and $446 billion per year (in 2019 prices), depending on the levels of physical activity, but did not report separately the potential direct costs to health-care systems.^27^ Given the scarce published evidence on the economic impact of physical inactivity on public health-care systems, our study contributes useful new data and aims to stimulate further research.

We used the latest available prevalence estimates of physical inactivity from WHO and forecasted estimates of disease incidence for seven health outcomes. However, we are likely to have underestimated the total cost of physical inactivity as we only considered expenditures related to the initial treatment of new cases of NCDs (usually costs related to the first year of treatment) per year, not the accumulated costs of the treatment, complications, and management of these new cases over the years; nor did we include the costs associated with mortality. Chronic conditions, and particularly multiple chronic conditions per patient, accrue a disproportionate burden to health systems and so inclusion would greatly increase the economic impact of physical inactivity to public health care. Furthermore, because of methodological challenges, we did not include the costs associated with productivity losses or deaths. The addition of these costs would better reflect the higher health and economic burden of physical inactivity.

Additionally, our analysis used a narrow definition of physical inactivity that excluded details on muscle strengthening because of the absence of any global data. If muscle strengthening were included, the prevalence of physical inactivity would likely be higher and its inclusion would result in even greater costs to public health systems. Future studies should use a wider definition of physical inactivity when appropriate global data are available.

Lastly, when country data were not available on health-care costs, we extrapolated costs from the EU28 to other countries and assumed, as in Ding and colleagues,^5^ that the cross-country differences in disease-specific costs per case are solely driven by the differences in overall health-care expenditure per capita. Given the scarcity of costing data, especially in LMICs, the methodology of using weighting factor to extrapolate EU28 costs is arguably the best available approach as a proxy to calculate health-care costs in lower-income settings.^5^ However, better data and further methodological development are needed to produce more robust and contextualised evidence in lower-income settings. Despite these limitations, this study provides important new insights on the global health and economic burden of physical inactivity to public health-care systems in all countries.

The results of this study should not be interpreted as the total cost that would be avoided if physical inactivity were reduced. An underlying assumption of the PAF is that the removal of the exposure would not affect other risk factors.^28^ This assumption might not hold because, for example, individuals who become more active might also adopt a better diet, stop smoking, and decrease alcohol consumption, making the interpretation of the physical activity PAF for diseases difficult. Additionally, an intervention will not necessarily lead to the complete removal of new cases of diseases, corresponding to an effectiveness of 100%, even if a multisectoral approach is implemented, as recommended in the WHO Global Action Plan on Physical Activity 2018–30. Even if programmes and infrastructure are provided to support people to be more active, not everyone would opt to be physically active.^6^, ^28^ Thus, for this study, we cautiously interpret the PAF as the proportion of disease risk in the total population that could be reduced or eliminated.

Additionally, estimating the total avoidable costs of physical inactivity requires calculating the net cost of interventions implemented to increase physical activity—ie, the total benefits of reducing levels of physical inactivity minus the costs of interventions to promote physical activity.^3^ Future work should undertake net cost analyses once the necessary data on an agreed set of interventions are available.

All government-policy decision makers are faced with competing demands on limited financial and human resources and consequently require information on the cost of not acting to increase physical activity. These data can support the development and strengthening of national investment cases and resource mobilisation to support the implementation of policy and programmes that enable more people to be more active, more often.

Modelling of cost-of-inaction and return-on-investment analyses have been important contributors to national and global progress on other NCD risk factors such as tobacco control.^3^ Yet, to date, few economic analyses have been done on physical inactivity despite recognition of the urgent need for such research.^5^ This study shows that without effective action to increase levels of physical activity by 2030, countries will incur substantial costs in terms of preventable new cases of NCDs. Additionally, countries will not reap the associated benefits that increasing physical activity can have on other important national agendas, such as reducing air pollution and fossil fuel consumption through increased walking and cycling and the wider effects of increasing sports participation on social and economic development.^6^

Despite WHO's Global Action Plan on Physical Activity providing countries with clear guidance on a set of effective and adaptable physical activity interventions, national progress on implementing relevant policy has been slow and global levels of physical activity in adults remain little changed in over a decade.^6^ The first WHO Global Status Report on Physical Activity reveals that although most countries report having a national policy on NCDs, which includes physical activity, a profound gap exists in relevant policy implementation.^29^ This inaction perpetuates inequalities in levels of physical activity and health. Given that only 8 years remain to achieve the global target to reduce physical inactivity by a relative 15% from the 2010 baseline, the findings of this study should be used by advocates and decision makers to encourage mobilisation of resources and acceleration of the implementation of national policy commitments to reducing physical inactivity.^6^ Additionally, global NCD prevention and management initiatives such as HEARTS,^30^ Diabetes Compact,^31^ Resolve to Save Lives, Decade of Healthy Ageing,^32^ Mental Health Action Plan 2013–30,^33^ and Second Decade of Action on Road Safety^34^ should be used as opportunities to strengthen alignment and implementation of interventions on physical activity. Finally, the scientific community is encouraged to further advance methods and evidence on the health system and societal impact and economic returns from increasing physical activity. Coordinated and accelerated action to reduce the prevalence of physical inactivity will make a substantial impact and further investments and scaling up of multisectoral actions are needed to achieve this aim.^7^

## Data sharing

Except for the cost estimates for incident cancers, no original data were collected for this research. Data on cost estimates for incident cancers are available in the appendix (p 24). All other data used in this analysis are already in the public domain.

## Declaration of interests

We declare no competing interests.
