# Supplementary material for: The cost of inaction on physical inactivity to public health-care systems: a population-attributable fraction analysis
Source: Lancet Glob Health. 2022 Dec 5;11(1):e32–9. doi: 10.1016/S2214-109X(22)00464-8 (PMC9748301; doi:10.1016/S2214-109X(22)00464-8)
Supplement: Supplementary appendix [file mmc1.pdf]

THE LANCET
Global Health

Supplementary appendix

This appendix formed part of the original submission and has been peer reviewed. We post it as supplied by the authors.

Supplement to: Santos AC, Willumsen J, Meheus F, Ilbawi A, Bull FC. The cost of inaction on physical inactivity to public health-care systems: a population-attributable fraction analysis. *Lancet Glob Health* 2022; published online Dec 5. https://doi.org/10.1016/S2214-109X(22)00464-8.

SUPPLEMENTARY MATERIAL

Table of Contents

1. A conceptual framework ... 2

Figure S1. Conceptual framework ... 2

2. Discussion of the incidence and prevalence approaches to calculating the cost of illness ... 3

3. Methodological steps to estimate the economic cost of NCDs & mental health conditions per year attributable to physical inactivity ... 4

Figure S2. Methodological steps to estimate the economic cost of NCDs & mental health conditions per year attributable to physical inactivity ... 4

4. Additional information about the sources of cost data for dementia and diabetes ... 5

5. Methods for assessing uncertainly ... 6

References ... 7

Table S1. Summary of the adjusted relative risks per selected health outcomes, comparisons for summary relative risks, and number of studies and individuals included ... 9

Table S2. Estimated populational attributable fraction (PAFs) by health outcomes and countries (% and 95% confidence interval), 2020 ... 10

Table S3. Estimated populational attributable fraction (PAFs) by health outcomes, WHO Regions, and World Bank income classification (% and 95% confidence interval), 2020 ... 23

Table S4. Estimated direct healthcare cost per incident case of cancer by WHO Region and country in US$ 2020 ... 24

Table S5. Estimated direct healthcare costs for stroke, coronary heart disease and hypertension, by country and WHO Regions, in US$ 2020 ... 30

Table S6. Total number of estimated cases by health outcomes attributed to physical inactivity by country, 2020-2030 ... 35

Table S7. Total number of estimated cases by health outcomes attributed to physical inactivity by WHO Regions and World Bank income classification, 2020-2030 ... 49

Table S8. Direct healthcare costs attributable to physical inactivity, by health outcomes and by country (in US$), 2020-2030 ... 51

Table S9. Direct healthcare costs attributable to physical inactivity, by health outcomes and by country (in 1,000,000 INT$), 2020-2030 ... 68

Table S10. Direct healthcare costs attributable to physical inactivity, by health outcomes, WHO regions and World Bank income classification (in US$), 2020-2030 ... 75

Table S11. Sensitivity analysis: varying costs of incident cases by -30% and -50%, by WHO Regions and World Bank income classification, in US$, 2020-2030 ... 77

Appendix 1. WHO Regions and countries ... 78

Appendix 2. Estimating the costs for diabetes complications: the approach used to avoid double counting ... 79

1. A conceptual framework

Figure S1 presents a conceptual framework for the study and the analytical associations between the key variables used to estimate the burden of diseases attributable to physical inactivity. It shows that levels of **physical inactivity** are associated with **new (incident) cases of NCDs and mental health conditions** and the other established risk factors that can also influence the risk of developing these conditions (for example, the development of coronary heart disease (CHD) is associated with other known risk factors, including, tobacco use, harmful use of alcohol, unhealthy diet and air pollution as well as hypertension and diabetes, and other socio-economic characteristics of individuals (e.g., level of education)). [1–3] Other non-modifiable factors also affect the likelihood of NCD and mental health conditions such as age, sex, genetic predisposition, and unknown risk.[1] Collectively these are known as **confounders**, and are factors that influence both the exposure (i.e., physical inactivity) and the NCDs or mental health outcomes.[4]

Relative risk (RR) is a measure commonly used to represent the effect of physical inactivity on diseases.[5] It measures the ratio of the probability of a disease outcome (e.g. CHD) in the physically inactive group (known as 'exposed') to the probability of CHD in the physically active group (known as 'unexposed'). It can be adjusted or not adjusted for potential confounders factors (crude RR).[6] For this study, we used an adjusted RR.

The public health impact of new cases of NCDs and mental health conditions attributable to physical inactivity, can be estimated using the **population attributable fraction (PAF)**, which is in function of the (adjusted) RRs and the prevalence of physical inactivity (exposure).[7] PAF provides an estimate of the proportion of the disease that can be attributed to physical inactivity.

Figure S1. Conceptual framework

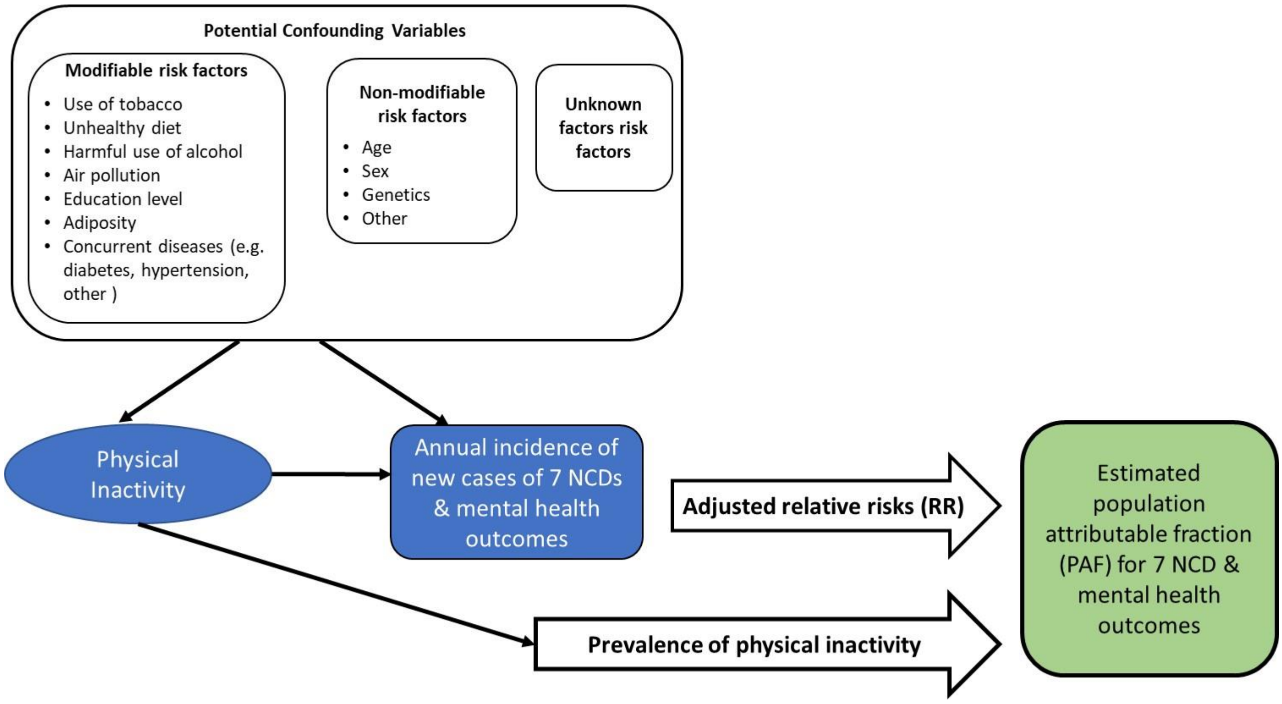

2. Discussion of the incidence and prevalence approaches to calculating the cost of illness

There are two main approaches used to estimate the cost of illness: the prevalence and the incident approaches.[8] In the prevalence approach, the direct costs for any disease or group of diseases are estimated.[8] Prevalence includes new cases and existing cases (and costs) of diseases occurring in a given period (typically, in a year).[9] The prevalence approach can answer the question of *what are the current (and future) costs of existing disease (s)?* The prevalence approach would help decision-makers understand the current economic burden of disease to plan their budget allocation for the *management of existing diseases*, in the present and make provisions for future years. However, this approach **cannot** answer questions *what is the proportion of costs (and cases) that could be eliminated or reduced if a risk factor is eliminated or reduced (e.g., physical inactivity)?*[10] This question is of importance to policy makers in their decisions about resource allocation for investments in health prevention and promotion, the foundations of the UN Sustainable Development Goals (SDGs).

The **incidence approach** is typically based on microsimulation models. Microsimulation models are the most rigorous and common approach used for modelling and projecting the long-term health impacts of a disease.[11,12] It starts with a cohort of new cases of disease and follows this cohort for life (this is different from using prevalent cases), until death, and assesses the costs of complications that can occur for those new cases in a lifetime (ideal time horizon). The complications are defined by health status (proportion) and transition probabilities (e.g., the probability of an individual progressing from one health status, say, from diabetic nephropathy to another, say end-stage renal disease, or to death).[13] Although a microsimulation incidence approach is considered the "gold standard" for economic analysis, there is a lack of data available for health status and transition probabilities on a worldwide scale to allow us to conduct this assessment. Health status and transition probabilities are dependent on healthcare quality and accessibility, household socio-economic background, and affordability, which differ largely amongst countries, even among high-income countries.

3. **Methodological steps to estimate the economic cost of NCDs & mental health conditions per year attributable to physical inactivity**

Figure S2 shows the key steps undertaken to estimate the economic cost of NCDs & mental health conditions per year attributable to physical inactivity - referred to as the "cost of inaction" on physical inactivity.

Step 1 combined information of the adjusted RRs and the prevalence of physical inactivity to estimate the PAF. The PAF was multiplied by the estimates of annual incidence of seven NCDs and mental conditions in 2020. We then applied UN population projections to estimate the annual incidence of seven NCDs and mental conditions from 2020 to 2030 (health burden). We opted to use a **simple incidence approach** where we estimated the *yearly* attributable health burden for each disease associated with physical inactivity (incident cases occurring yearly *x* PAF) – this approach also helps to minimise the potential effect of diseases overlapping for the assessment of the economic burden of physical inactivity as a risk factor.

Step 2 multiplied the annual health burden of each disease attributed to physical inactivity by the estimated annual average direct healthcare cost per disease, which was projected from 2020 to 2030 by using inflation rates as estimated by the World Bank[14], to produce an estimate attributable economic burden of diseases associated with physical inactivity.

Figure S2. Methodological steps to estimate the cost of NCDs & mental health conditions per year attributable to physical inactivity

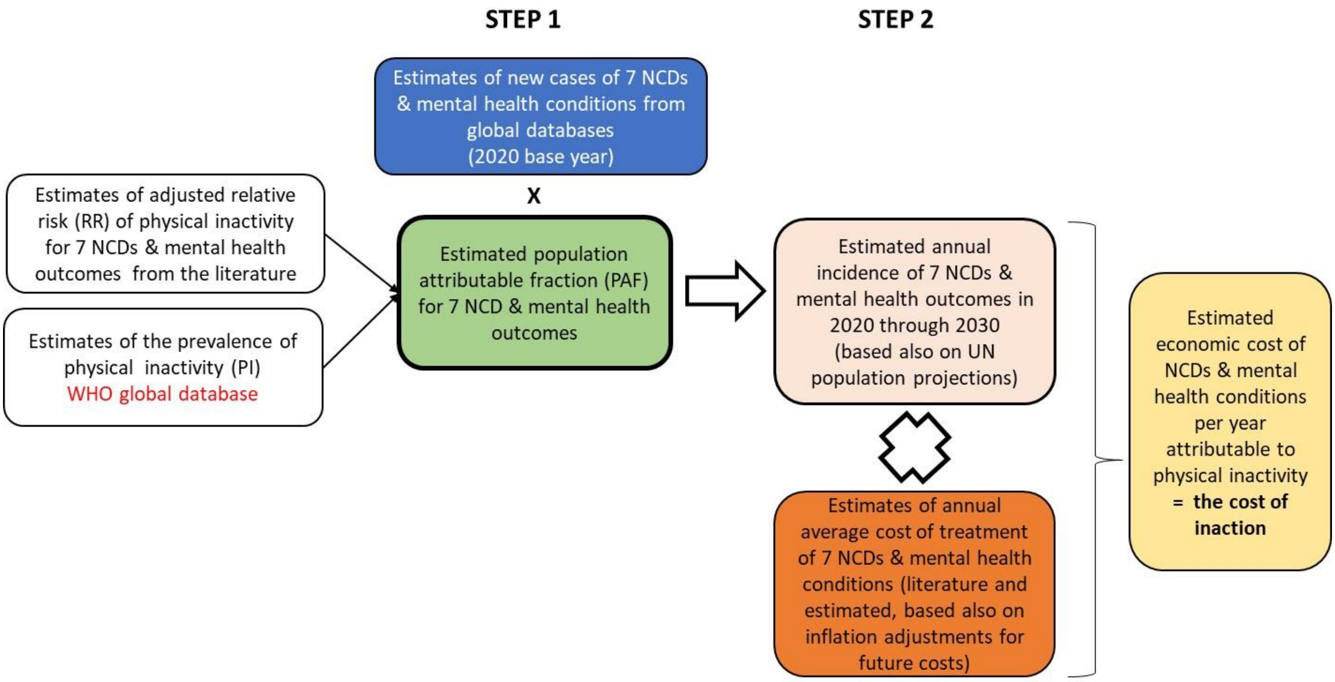

4. Additional information about the sources of cost data for dementia and diabetes

Dementia. Data were obtained from the WHO Mental Health Unit using findings from a commissioned systematic literature review assessing the use of resources and costs to treat dementia.[15] Direct medical care costs to the public health system was estimated as the costs of hospital care, drugs, diagnostic tests, and visits to clinics (specialist care, primary care). New cases of patients with dementia can arrive at the public health system in an initial or more advanced stage of the disease. The costs estimated by the WHO Unit assumed an average of these costs. Costs of more advanced stage dementia include those not always borne by the public health system, such as community services related to food supply ('meals on wheels'), transport, and residential or nursing home care/long-term care. Therefore, these potentially 'external' costs were not included. Direct medical costs per patient with dementia were estimated for the WHO Regions and World Bank country income levels, not by country.

Diabetes. Data were obtained from cost estimates developed by International Diabetes Federation (IDF) and they included the provision of health services (preventive and curative), family planning activities, nutrition activities, and emergency aid designated for health, which include mild to moderate cases (i.e., those manageable at primary care facilities and that usually do not require treatment for complications) and severe cases (e.g., those with kidney failure). We assessed from the literature that it is not uncommon for new patients of type 2 diabetes (or any other particular health outcome) to be diagnosed for the first time (incidence) and presented in the health system with complications, even in high-income countries. For example, a retrospective cohort study in the United States demonstrated that a substantial proportion of patients with type 2 diabetes had existing complications, especially chronic kidney disease.[16] Recognising that severe cases can overestimate the cost of illness, and reflecting on the fact that the costs of diabetes hugely vary among countries, reflecting not only the country's prevalence of the disease but also access to and quality of its health system, we decided to adopt an average cost for estimates by WHO Region and World Bank income classification, rather than median or weighted costs; thus, we avoided the influence of countries with big populations and big costs to the Region and income group levels. For example, the average annual cost of treatment for diabetes, per patient, in the U.S. was estimated by IDF at US$ 379,470 while for Brazil and Germany, it was estimated at US$ 42,928 and US$ 41,296, respectively.[17] The cost of almost 9 times in the U.S compared to Brazil and Germany essentially reflects the characteristics of their health system, where in the U.S. private insurance defines their prices, while in Brazil and Germany, the population has access to a public health system for treatment, although in Brazil with a much less extent coverage compared to Germany. We used IDF country-specific cost estimates to assess the costs at the country level.

5. Methods for assessing uncertainly

Uncertainty in economic and epidemiological analyses is typically related to four main areas: the variability of data (inputs), generalisability and extrapolation of data, and the appropriateness of the analytical method.[23] One-way or multivariate deterministic analysis, when one or multiple parameters are varied to assess uncertainties in the modelling, is the most common approach used in economic evaluation. It allows the analyst to assess the four main areas of uncertainty in economic studies as described above. However, some limitations are related to this method, the main ones being the choice of the variables and what alternative values are included in the sensitivity analysis, creating the potential for selection bias.[24] Alternatively, probabilistic sensitivity analysis, using Monte Carlo simulations would overcome this issue by computing confidence intervals for mean costs and effects, and allowing for a degree of variation in the results, producing, at least in theory, more robust results.[25]

To conduct a Monte Carlo probabilistic sensitivity analysis, one would need to define a variable quantity with a known range of possible values and an associated distribution function for each parameter (or at least, for the main parameters) used in the modelling.[26,27] For example, it would be necessary to know or make reasonable assumptions about the (assumed) variation of the upper- and lower-limit of the baseline values, their statistical distribution (e.g., normal, log-normal), and the standard deviation and standard error, for each variable. An accurate and robust Monte Carlo probabilistic sensitivity analysis is only possible to the extent that the assumed distributions and their parameters are correctly specified.[26,27]

However, we do not have the necessary data for the main parameters of our analysis, i.e., RRs, the prevalence of physical inactivity, and costing data; thus, assumptions would need to be made.

Costing data is the main source of uncertainty in our analysis, given that the RRs and their confidence intervals were estimated based on a strong GRADE assessment.[28] Similarly, the prevalence of physical inactivity has been standardised to remove confounding effects.[29]

Given the scope of analysis and the restriction of data, we opted for a one-way deterministic sensitivity analysis where we assumed a reduction in costs of 30% and 50% on the mean estimated costs. These parameters were based on discussions with experts from the WHO, and who have experience with the cost of disease treatment and management in low- and middle-income countries. They believe the range adopted would reflect the possible variation in costs in those income settings.

References

1 Havranek EP, Mujahid MS, Barr DA, *et al.* Social Determinants of Risk and Outcomes for Cardiovascular Disease: A Scientific Statement From the American Heart Association. *Circulation* 2015; **132**: 873–98.
2 Hajar R. Risk factors for coronary artery disease: Historical perspectives. *Heart Views* 2017; **18**: 109.
3 Joshi SS, Miller MR, Newby DE. Air pollution and cardiovascular disease: the Paul Wood Lecture, British Cardiovascular Society 2021. *Heart* 2022; **108**: 1267–73.
4 McNamee R. Confounding and confounders. *Occupational and Environmental Medicine* 2003; **60**: 227–34.
5 Katzmarzyk PT, Friedenreich C, Shiroma EJ, Lee I-M. Physical inactivity and non-communicable disease burden in low-income, middle-income and high-income countries. *Br J Sports Med* 2021; : bjsports-2020-103640.
6 Tenny S, Hoffman MR. Relative Risk. In: StatPearls. Treasure Island (FL): StatPearls Publishing, 2022. http://www.ncbi.nlm.nih.gov/books/NBK430824/ (accessed Oct 9, 2022).
7 Rockhill B, Newman B, Weinberg C. Use and misuse of population attributable fractions. *Am J Public Health* 1998; **88**: 15–9.
8 Tarricone R. Cost-of-illness analysis. *Health Policy* 2006; **77**: 51–63.
9 Sedgwick P. Prevalence and incidence. *BMJ* 2010; **341**: c4709–c4709.
10 Byford S. Economic Note: Cost of illness studies. *BMJ* 2000; **320**: 1335–1335.
11 Caro JJ, Briggs AH, Siebert U, Kuntz KM. Modeling Good Research Practices—Overview: A Report of the ISPOR-SMDM Modeling Good Research Practices Task Force-1. *Value in Health* 2012; **15**: 796–803.
12 Lay-Yee R, Cotterell G. The Role of Microsimulation in the Development of Public Policy. In: Janssen M, Wimmer MA, Deljoo A, eds. Policy Practice and Digital Science. Cham: Springer International Publishing, 2015: 305–20.
13 Krijkamp EM, Alarid-Escudero F, Enns EA, Jalal HJ, Hunink MGM, Pechlivanoglou P. Microsimulation Modeling for Health Decision Sciences Using R: A Tutorial. *Med Decis Making* 2018; **38**: 400–22.
14 World Bank. Inflation, consumer prices (annual %): International Monetary Fund, International Financial Statistics and data files. 2021. https://data.worldbank.org/indicator/FP.CPI.TOTL.ZG.
15 World Health Organization. Global status report on the public health response to dementia. Geneva: World Health Organization, 2021 https://apps.who.int/iris/handle/10665/344701 (accessed Nov 29, 2021).
16 An J, Nichols GA, Qian L, *et al.* Prevalence and incidence of microvascular and macrovascular complications over 15 years among patients with incident type 2 diabetes. *BMJ Open Diab Res Care* 2021; **9**: e001847.
17 International Diabetes Federation (IDF). Diabetes-related health expenditure per person, USD: IDF Diabetes Atlas 10th edition 2021. Diabetes-related health expenditure. https://diabetesatlas.org/data/en/indicators/19/ (accessed Nov 19, 2021).
18 National Health Services (NHS). National Cost Collection for the NHS, 2019-2020. 2020. https://www.england.nhs.uk/costing-in-the-nhs/national-cost-collection/ (accessed May 8, 2021).
19 OECD, European Union. Health at a Glance: Europe 2018: State of Health in the EU Cycle. OECD, 2018 DOI:10.1787/health_glance_eur-2018-en.
20 OECD. OECD Stats. OECD Stats. 2020. https://stats.oecd.org/ (accessed July 15, 2021).
21 Munich Center for the Economics of Aging (MEA). SHARE - Survey of Health, Ageing and Retirement in Europe. 2020. http://www.share-project.org/home0.html (accessed July 15, 2021).
22 Luengo-Fernandez R, Violato M, Candio P, Leal J. Economic burden of stroke across Europe: A population-based cost analysis. *European Stroke Journal* 2020; **5**: 17–25.
23 Briggs A, Sculpher M, Buxton M. Uncertainty in the economic evaluation of health care technologies: The role of sensitivity analysis. *Health Econ* 1994; **3**: 95–104.
24 Briggs AH. Handling Uncertainty in Cost-Effectiveness Models: *PharmacoEconomics* 2000; **17**: 479–500.
25 Halpern EF, Weinstein MC, Hunink MGM, Gazelle GS. Representing Both First- and Second-order Uncertainties by Monte Carlo Simulation for Groups of Patients. *Med Decis Making* 2000; **20**: 314–22.
26 Claxton K, Sculpher M, McCabe C, *et al.* Probabilistic sensitivity analysis for NICE technology assessment: not an optional extra. *Health Econ* 2005; **14**: 339–47.
27 Hatswell AJ, Bullement A, Briggs A, Paulden M, Stevenson MD. Probabilistic Sensitivity Analysis in Cost-Effectiveness Models: Determining Model Convergence in Cohort Models. *PharmacoEconomics* 2018; **36**: 1421–6.
28 World Health Organization. WHO Guidelines on Physical Activity and Sedentary Behaviour. 2020.
29 Guthold R, Stevens GA, Riley LM, Bull FC. Global trends in insufficient physical activity among adolescents: a pooled analysis of 298 population-based surveys with 1·6 million participants. *The Lancet Child & Adolescent Health* 2020; **4**: 23–35.
30 Schuch FB, Vancampfort D, Firth J, *et al.* Physical Activity and Incident Depression: A Meta-Analysis of Prospective Cohort Studies. *AJP* 2018; **175**: 631–48.
31 Kyu HH, Bachman VF, Alexander LT, *et al.* Physical activity and risk of breast cancer, colon cancer, diabetes, ischemic heart disease, and ischemic stroke events: systematic review and dose-response meta-analysis for the Global Burden of Disease Study 2013. *BMJ* 2016; : i3857.
32 Liu X, Zhang D, Liu Y, *et al.* Dose–Response Association Between Physical Activity and Incident Hypertension: A Systematic Review and Meta-Analysis of Cohort Studies. *Hypertension* 2017; **69**: 813–20.
33 Matthews CE, Moore SC, Arem H, *et al.* Amount and Intensity of Leisure-Time Physical Activity and Lower Cancer Risk. *JCO* 2020; **38**: 686–97.

34 Kivimäki M, Singh-Manoux A, Pentti J, *et al.* Physical inactivity, cardiometabolic disease, and risk of dementia: an individual-participant meta-analysis. *BMJ* 2019; : l1495.
35 Livingston G, Huntley J, Sommerlad A, *et al.* Dementia prevention, intervention, and care: 2020 report of the Lancet Commission. *The Lancet* 2020; **396**: 413–46.
36 The Emerging Risk Factors Collaboration. Diabetes mellitus, fasting blood glucose concentration, and risk of vascular disease: a collaborative meta-analysis of 102 prospective studies. *The Lancet* 2010; **375**: 2215–22.

Table S1. Summary of the adjusted relative risks per selected health outcomes, comparisons for summary relative risks, and number of studies and individuals included

Outcomes and sources of information	Relative risk (95% CI)	Comparisons for summary relative risk	Number of studies and individuals included[&]
Depression*<br>*Schuch et al., 2018*[30]	1·28 (1·01 to 1·62)	Lowest vs 150min of moderate-to-vigorous physical activity per week	49 prospective studies (266,939 individuals)
Coronary heart disease (CHD)*<br>*Kyu et al, 2016*[31]	1·19 (1·13 to 1·26)	<600 MET-min/week vs 600–3999 METmin/week of total physical activity across all domains	43 prospective studies (16,583,824 person years)
Stroke*<br>*Kyu et al, 2016*[31]	1·19 (1·09 to 1·28)	<600 MET-min/week vs 600–3999 METmin/week of total physical activity across all domains	26 prospective studies (13,670,573 person years)
Type 2 diabetes*<br>*Kyu et al, 2016*[31]	1·17 (1·11 to 1·23)	<600 MET-min/week vs 600–3999 METmin/week of total physical activity across all domains	55 prospective studies (14,051,132 person years),
Hypertension*<br>*Liu et al., 2017*[32]	1·06 (1·03 to 1·09)	None vs 10 MET-hour/week of leisure-time physical activity	24 prospective studies (330,222 individuals)
Bladder cancer*<br>*Matthews et al., 2020*[33]	1·08 (0·93 to 1·25)	For all cancers outcomes:<br>None vs 7·5–14·9 MET-hour/week of leisure-time physical activity	Basis for all cancers outcomes: 9 prospective studies (755,459 individuals)
Breast cancer*<br>*Matthews et al., 2020*[33]	1·09 (1·03 to 1·15)		
Colon cancer*<br>*Matthews et al., 2020*[33]	1·11 (1·03 to 1·19)		
Endometrial cancer*<br>*Matthews et al., 2020*[33]	1·09 (0·96 to 1·22)		
Oesophageal cancer*<br>*Matthews et al., 2020*[33]	1·28 (0·85 to 1·96)		
Gastric cancer*<br>*Matthews et al., 2020*[33]	1·27 (0·93 to 1·69)		
Renal cancer*<br>*Matthews et al., 2020*[33]	1·28 (1·06 to 1·54)		
Dementia**<br>*Kivimäki et al., 2019*[34]	1·40 (1·20 to 1·70)	Low vs moderate to vigorous physical activity	19 observational studies (404,840 individuals)

CI: confidence interval; * as reported by Katzmarzyk and colleagues (2021);[5] **as reported by Livingstone and colleagues (2020);[35] [&]number of studies included in the meta-analysis or pooled-analysis.

Table S2. Estimated populational attributable fraction (PAFs) by health outcomes and countries (% and 95% confidence interval), 2020

African Region	Breast cancer	Colorectal cancer	Endometrial cancer	Gastric cancer	Oseophageal cancer	Renal cancer
Algeria	0·03 (0·01 to 0·06)	0·04 (0·01 to 0·06)	0·03 (-0·02 to 0·08)	0·08 (-0·02 to 0·19)	0·09 (-0·05 to 0·24)	0·09 (0·02 to 0·15)
Angola	0·02 (0·01 to 0·04)	0·02 (0·01 to 0·03)	0·02 (-0·01 to 0·05)	0·05 (-0·01 to 0·11)	0·05 (-0·03 to 0·15)	0·05 (0·01 to 0·09)
Benin	0·02 (0·01 to 0·03)	0·02 (0 to 0·03)	0·02 (-0·01 to 0·04)	0·04 (-0·01 to 0·1)	0·04 (-0·02 to 0·13)	0·04 (0·01 to 0·08)
Botswana	0·02 (0·01 to 0·04)	0·02 (0·01 to 0·04)	0·02 (-0·01 to 0·05)	0·06 (-0·02 to 0·13)	0·06 (-0·03 to 0·17)	0·06 (0·01 to 0·1)
Burkina Faso	0·02 (0·01 to 0·03)	0·02 (0·01 to 0·04)	0·02 (-0·01 to 0·05)	0·05 (-0·01 to 0·12)	0·05 (-0·03 to 0·16)	0·05 (0·01 to 0·1)
Burundi	0·02 (0·01 to 0·04)	0·02 (0·01 to 0·03)	0·02 (-0·01 to 0·05)	0·05 (-0·01 to 0·11)	0·05 (-0·03 to 0·15)	0·05 (0·01 to 0·09)
Cabo Verde	0·02 (0·01 to 0·04)	0·02 (0·01 to 0·04)	0·02 (-0·01 to 0·05)	0·05 (-0·01 to 0·12)	0·05 (-0·03 to 0·16)	0·05 (0·01 to 0·1)
Cameroon	0·03 (0·01 to 0·05)	0·03 (0·01 to 0·05)	0·03 (-0·01 to 0·07)	0·07 (-0·02 to 0·16)	0·07 (-0·04 to 0·21)	0·07 (0·02 to 0·13)
Central Africa Republic	0·01 (0 to 0·02)	0·02 (0 to 0·03)	0·01 (-0·01 to 0·03)	0·04 (-0·01 to 0·09)	0·04 (-0·02 to 0·12)	0·04 (0·01 to 0·07)
Chad	0·02 (0·01 to 0·04)	0·02 (0·01 to 0·04)	0·02 (-0·01 to 0·06)	0·06 (-0·02 to 0·14)	0·06 (-0·04 to 0·18)	0·06 (0·01 to 0·11)
Comoros (The)	0·02 (0·01 to 0·03)	0·02 (0 to 0·03)	0·02 (-0·01 to 0·04)	0·04 (-0·01 to 0·09)	0·04 (-0·02 to 0·12)	*
Congo	0·03 (0·01 to 0·04)	0·03 (0·01 to 0·05)	0·03 (-0·01 to 0·06)	0·07 (-0·02 to 0·16)	0·07 (-0·04 to 0·21)	0·07 (0·02 to 0·13)
Côte d'Ivoire	0·03 (0·01 to 0·05)	0·04 (0·01 to 0·06)	0·03 (-0·02 to 0·08)	0·08 (-0·02 to 0·19)	0·08 (-0·05 to 0·24)	0·08 (0·02 to 0·15)
Democratic Republic of the Congo	0·02 (0·01 to 0·04)	0·03 (0·01 to 0·04)	0·02 (-0·01 to 0·06)	0·06 (-0·02 to 0·14)	0·06 (-0·04 to 0·19)	0·06 (0·01 to 0·11)
Equatorial Guinea	0·02 (0·01 to 0·04)	0·02 (0·01 to 0·03)	0·02 (-0·01 to 0·05)	0·05 (-0·01 to 0·11)	0·05 (-0·03 to 0·15)	0·05 (0·01 to 0·09)
Eritrea	0·03 (0·01 to 0·04)	0·02 (0·01 to 0·04)	0·03 (-0·01 to 0·06)	0·06 (-0·02 to 0·13)	0·06 (-0·03 to 0·18)	0·06 (0·01 to 0·11)
Eswatini	0·03 (0·01 to 0·05)	0·03 (0·01 to 0·05)	0·03 (-0·01 to 0·07)	0·07 (-0·02 to 0·16)	0·07 (-0·04 to 0·21)	0·07 (0·02 to 0·13)
Ethiopia	0·02 (0·01 to 0·03)	0·02 (0 to 0·03)	0·02 (-0·01 to 0·04)	0·04 (-0·01 to 0·09)	0·04 (-0·02 to 0·13)	0·04 (0·01 to 0·07)
Gabon	0·03 (0·01 to 0·05)	0·03 (0·01 to 0·05)	0·03 (-0·01 to 0·07)	0·06 (-0·02 to 0·15)	0·07 (-0·04 to 0·2)	0·07 (0·01 to 0·12)
Ghana	0·02 (0·01 to 0·04)	0·02 (0·01 to 0·04)	0·02 (-0·01 to 0·05)	0·06 (-0·02 to 0·13)	0·06 (-0·03 to 0·17)	0·06 (0·01 to 0·11)
Guinea	0·02 (0·01 to 0·03)	0·02 (0 to 0·03)	0·02 (-0·01 to 0·04)	0·04 (-0·01 to 0·09)	0·04 (-0·02 to 0·12)	0·04 (0·01 to 0·07)
Guinea-Bissau	0·02 (0·01 to 0·04)	0·02 (0·01 to 0·03)	0·02 (-0·01 to 0·05)	0·05 (-0·01 to 0·11)	0·05 (-0·03 to 0·15)	0·05 (0·01 to 0·09)
Kenya	0·01 (0·01 to 0·02)	0·02 (0 to 0·03)	0·01 (-0·01 to 0·04)	0·04 (-0·01 to 0·1)	0·04 (-0·02 to 0·13)	0·04 (0·01 to 0·08)
Lesotho	0·01 (0 to 0·01)	0·01 (0 to 0·01)	0·01 (0 to 0·01)	0·02 (0 to 0·04)	0·02 (-0·01 to 0·06)	0·02 (0 to 0·03)
Liberia	0·03 (0·01 to 0·04)	0·03 (0·01 to 0·05)	0·03 (-0·01 to 0·06)	0·06 (-0·02 to 0·15)	0·07 (-0·04 to 0·19)	0·07 (0·01 to 0·12)
Madagascar	0·02 (0·01 to 0·03)	0·02 (0·01 to 0·03)	0·02 (-0·01 to 0·04)	0·04 (-0·01 to 0·11)	0·05 (-0·03 to 0·14)	0·05 (0·01 to 0·08)
Malawi	0·02 (0·01 to 0·03)	0·02 (0 to 0·03)	0·02 (-0·01 to 0·04)	0·04 (-0·01 to 0·1)	0·04 (-0·02 to 0·13)	0·04 (0·01 to 0·08)
Mali	0·04 (0·01 to 0·07)	0·04 (0·01 to 0·07)	0·04 (-0·02 to 0·09)	0·1 (-0·03 to 0·22)	0·1 (-0·06 to 0·28)	0·1 (0·02 to 0·18)
Mauritania	0·04 (0·01 to 0·06)	0·04 (0·01 to 0·07)	0·04 (-0·02 to 0·09)	0·1 (-0·03 to 0·22)	0·1 (-0·07 to 0·28)	0·1 (0·02 to 0·18)
Mauritius	0·03 (0·01 to 0·05)	0·03 (0·01 to 0·05)	0·03 (-0·01 to 0·07)	0·07 (-0·02 to 0·17)	0·08 (-0·05 to 0·22)	0·08 (0·02 to 0·14)

Mozambique	0·01 (0 to 0·01)	0·01 (0 to 0·01)	0·01 (0 to 0·01)	0·01 (0 to 0·04)	0·02 (-0·01 to 0·05)	0·02 (0 to 0·03)
Namibia	0·03 (0·01 to 0·05)	0·04 (0·01 to 0·06)	0·03 (-0·02 to 0·08)	0·08 (-0·02 to 0·19)	0·09 (-0·05 to 0·24)	0·09 (0·02 to 0·15)
Niger	0·02 (0·01 to 0·04)	0·02 (0·01 to 0·04)	0·02 (-0·01 to 0·05)	0·06 (-0·02 to 0·13)	0·06 (-0·03 to 0·18)	0·06 (0·01 to 0·11)
Nigeria	0·03 (0·01 to 0·04)	0·03 (0·01 to 0·05)	0·03 (-0·01 to 0·06)	0·07 (-0·02 to 0·16)	0·07 (-0·04 to 0·21)	0·07 (0·02 to 0·13)
Rwanda	0·02 (0·01 to 0·03)	0·02 (0 to 0·03)	0·02 (-0·01 to 0·04)	0·04 (-0·01 to 0·09)	0·04 (-0·02 to 0·12)	0·04 (0·01 to 0·07)
Sao Tome and Principe	0·02 (0·01 to 0·03)	0·02 (0 to 0·03)	*	0·04 (-0·01 to 0·1)	0·04 (-0·02 to 0·13)	0·04 (0·01 to 0·08)
Senegal	0·02 (0·01 to 0·04)	0·02 (0·01 to 0·04)	0·02 (-0·01 to 0·06)	0·06 (-0·02 to 0·14)	0·06 (-0·04 to 0·18)	0·06 (0·01 to 0·11)
Seychelles	*	*	*	*	*	*
Sierra Leone	0·02 (0·01 to 0·03)	0·02 (0 to 0·03)	0·02 (-0·01 to 0·04)	0·04 (-0·01 to 0·09)	0·04 (-0·02 to 0·12)	0·04 (0·01 to 0·07)
South Africa	0·04 (0·01 to 0·07)	0·04 (0·01 to 0·07)	0·04 (-0·02 to 0·09)	0·09 (-0·03 to 0·21)	0·1 (-0·06 to 0·27)	0·1 (0·02 to 0·17)
South Sudan	0·02 (0·01 to 0·04)	0·02 (0·01 to 0·03)	0·02 (-0·01 to 0·05)	0·05 (-0·01 to 0·11)	0·05 (-0·03 to 0·15)	0·05 (0·01 to 0·09)
the Gambia	0·02 (0·01 to 0·04)	0·02 (0·01 to 0·04)	0·02 (-0·01 to 0·05)	0·05 (-0·01 to 0·13)	0·06 (-0·03 to 0·17)	*
Togo	0·01 (0 to 0·02)	0·01 (0 to 0·02)	0·01 (0 to 0·02)	0·03 (-0·01 to 0·06)	0·03 (-0·01 to 0·09)	0·03 (0·01 to 0·05)
Uganda	0·01 (0 to 0·01)	0·01 (0 to 0·01)	0·01 (0 to 0·01)	0·01 (0 to 0·04)	0·02 (-0·01 to 0·05)	0·02 (0 to 0·03)
United Republic of Tanzania	0·01 (0 to 0·01)	0·01 (0 to 0·01)	0·01 (0 to 0·02)	0·02 (0 to 0·04)	0·02 (-0·01 to 0·06)	0·02 (0 to 0·03)
Zambia	*	*	*	*	*	*
Zimbabwe	*	*	*	*	*	*
**Region of the Americas**						
Antigua and Barbuda	*	*	*	*	*	*
Argentina	0·04 (0·01 to 0·06)	0·04 (0·01 to 0·07)	0·04 (-0·02 to 0·09)	0·1 (-0·03 to 0·22)	0·1 (-0·07 to 0·29)	0·1 (0·02 to 0·18)
Bahamas (The)	0·05 (0·02 to 0·08)	0·05 (0·01 to 0·08)	0·05 (-0·02 to 0·11)	0·1 (-0·03 to 0·23)	0·11 (-0·07 to 0·29)	0·11 (0·03 to 0·19)
Barbados	0·05 (0·02 to 0·08)	0·05 (0·01 to 0·08)	0·05 (-0·02 to 0·11)	0·1 (-0·03 to 0·23)	0·11 (-0·07 to 0·29)	0·11 (0·03 to 0·19)
Belize	0·04 (0·01 to 0·06)	0·04 (0·01 to 0·06)	0·04 (-0·02 to 0·09)	0·09 (-0·03 to 0·19)	0·09 (-0·06 to 0·25)	0·09 (0·02 to 0·16)
Bolivia (Plurinational State of)	0·04 (0·01 to 0·06)	0·04 (0·01 to 0·06)	0·04 (-0·02 to 0·09)	0·09 (-0·03 to 0·19)	0·09 (-0·06 to 0·25)	0·09 (0·02 to 0·16)
Brazil	0·05 (0·02 to 0·07)	0·05 (0·01 to 0·08)	0·05 (-0·02 to 0·1)	0·11 (-0·03 to 0·24)	0·12 (-0·08 to 0·31)	0·12 (0·03 to 0·2)
Canada	0·03 (0·01 to 0·04)	0·03 (0·01 to 0·05)	0·03 (-0·01 to 0·06)	0·07 (-0·02 to 0·16)	0·07 (-0·04 to 0·22)	0·07 (0·02 to 0·13)
Chile	0·03 (0·01 to 0·04)	0·03 (0·01 to 0·05)	0·03 (-0·01 to 0·06)	0·07 (-0·02 to 0·16)	0·07 (-0·04 to 0·2)	0·07 (0·02 to 0·13)
Colombia	0·04 (0·01 to 0·07)	0·05 (0·01 to 0·08)	0·04 (-0·02 to 0·1)	0·11 (-0·03 to 0·23)	0·11 (-0·07 to 0·3)	0·11 (0·03 to 0·19)
Costa Rica	0·05 (0·02 to 0·08)	0·05 (0·01 to 0·08)	0·05 (-0·02 to 0·11)	0·11 (-0·03 to 0·24)	0·11 (-0·07 to 0·31)	0·11 (0·03 to 0·2)
Cuba	0·04 (0·01 to 0·06)	0·04 (0·01 to 0·07)	0·04 (-0·02 to 0·09)	0·09 (-0·03 to 0·2)	0·09 (-0·06 to 0·26)	0·09 (0·02 to 0·17)
Dominica	*	*	*	*	*	*
Dominican Republic (The)	0·04 (0·01 to 0·06)	0·04 (0·01 to 0·07)	0·04 (-0·02 to 0·09)	0·1 (-0·03 to 0·21)	0·1 (-0·06 to 0·27)	0·1 (0·02 to 0·17)

Ecuador	0·03 (0·01 to 0·04)	0·03 (0·01 to 0·05)	0·03 (-0·01 to 0·06)	0·07 (-0·02 to 0·16)	0·07 (-0·04 to 0·21)	0·07 (0·02 to 0·13)
El Salvador	0·04 (0·01 to 0·06)	0·04 (0·01 to 0·06)	0·04 (-0·02 to 0·09)	0·09 (-0·03 to 0·19)	0·09 (-0·06 to 0·25)	0·09 (0·02 to 0·16)
Grenada	*	*	*	*	*	*
Guatemala	0·03 (0·01 to 0·05)	0·04 (0·01 to 0·07)	0·03 (-0·02 to 0·08)	0·09 (-0·03 to 0·2)	0·09 (-0·06 to 0·26)	0·09 (0·02 to 0·17)
Guyana	0·04 (0·01 to 0·06)	0·04 (0·01 to 0·06)	0·04 (-0·02 to 0·09)	0·09 (-0·03 to 0·19)	0·09 (-0·06 to 0·25)	0·09 (0·02 to 0·16)
Haiti	0·04 (0·01 to 0·06)	0·04 (0·01 to 0·06)	0·04 (-0·02 to 0·09)	0·09 (-0·03 to 0·19)	0·09 (-0·06 to 0·25)	0·09 (0·02 to 0·16)
Honduras	0·04 (0·01 to 0·06)	0·04 (0·01 to 0·06)	0·04 (-0·02 to 0·09)	0·09 (-0·03 to 0·19)	0·09 (-0·06 to 0·25)	0·09 (0·02 to 0·16)
Jamaica	0·03 (0·01 to 0·05)	0·03 (0·01 to 0·06)	0·03 (-0·01 to 0·07)	0·08 (-0·02 to 0·18)	0·08 (-0·05 to 0·24)	0·08 (0·02 to 0·15)
Mexico	0·03 (0·01 to 0·05)	0·03 (0·01 to 0·05)	0·03 (-0·01 to 0·07)	0·07 (-0·02 to 0·17)	0·07 (-0·05 to 0·22)	0·07 (0·02 to 0·13)
Nicaragua	0·04 (0·01 to 0·06)	0·04 (0·01 to 0·06)	0·04 (-0·02 to 0·09)	0·09 (-0·03 to 0·19)	0·09 (-0·06 to 0·25)	0·09 (0·02 to 0·16)
Panama	0·04 (0·01 to 0·06)	0·04 (0·01 to 0·06)	0·04 (-0·02 to 0·09)	0·09 (-0·03 to 0·19)	0·09 (-0·06 to 0·25)	0·09 (0·02 to 0·16)
Paraguay	0·03 (0·01 to 0·05)	0·04 (0·01 to 0·07)	0·03 (-0·01 to 0·07)	0·09 (-0·03 to 0·21)	0·09 (-0·06 to 0·26)	0·09 (0·02 to 0·17)
Peru	0·04 (0·01 to 0·06)	0·04 (0·01 to 0·06)	0·04 (-0·02 to 0·09)	0·09 (-0·03 to 0·19)	0·09 (-0·06 to 0·25)	0·09 (0·02 to 0·16)
Saint Kitts and Nevis	*	*	*	*	*	*
Saint Lucia	0·04 (0·02 to 0·07)	0·04 (0·01 to 0·07)	0·04 (-0·02 to 0·1)	0·1 (-0·03 to 0·22)	0·1 (-0·06 to 0·28)	0·1 (0·02 to 0·18)
Saint Vicent and the Grenadines	*	*	*	*	*	*
Suriname	0·04 (0·01 to 0·07)	0·05 (0·01 to 0·08)	0·04 (-0·02 to 0·1)	0·11 (-0·03 to 0·23)	0·11 (-0·07 to 0·3)	0·11 (0·03 to 0·19)
Trinidad and Tobago	0·04 (0·01 to 0·07)	0·04 (0·01 to 0·07)	0·04 (-0·02 to 0·1)	0·09 (-0·03 to 0·21)	0·1 (-0·06 to 0·27)	0·1 (0·02 to 0·17)
United States of America	0·04 (0·01 to 0·07)	0·04 (0·01 to 0·07)	0·04 (-0·02 to 0·1)	0·1 (-0·03 to 0·22)	0·1 (-0·06 to 0·28)	0·1 (0·02 to 0·18)
Uruguay	0·02 (0·01 to 0·04)	0·02 (0·01 to 0·04)	0·02 (-0·01 to 0·05)	0·06 (-0·02 to 0·13)	0·06 (-0·03 to 0·18)	0·06 (0·01 to 0·11)
Venezuela (Bolivarian Republic of)	0·03 (0·01 to 0·05)	0·03 (0·01 to 0·06)	0·03 (-0·01 to 0·07)	0·08 (-0·02 to 0·18)	0·08 (-0·05 to 0·23)	0·08 (0·02 to 0·14)
**Eastern Mediterran Region**						
Afghanistan	0·04 (0·01 to 0·06)	0·04 (0·01 to 0·07)	0·04 (-0·02 to 0·09)	0·1 (-0·03 to 0·21)	0·1 (-0·06 to 0·27)	0·1 (0·02 to 0·18)
Bahrain	0·04 (0·01 to 0·06)	0·04 (0·01 to 0·07)	0·04 (-0·02 to 0·09)	0·1 (-0·03 to 0·21)	0·1 (-0·06 to 0·27)	0·1 (0·02 to 0·18)
Egypt	0·03 (0·01 to 0·05)	0·03 (0·01 to 0·06)	0·03 (-0·02 to 0·08)	0·08 (-0·02 to 0·18)	0·08 (-0·05 to 0·23)	0·08 (0·02 to 0·14)
Iran (Islamic Republic of)	0·04 (0·01 to 0·06)	0·04 (0·01 to 0·06)	0·04 (-0·02 to 0·09)	0·08 (-0·02 to 0·19)	0·09 (-0·05 to 0·24)	0·09 (0·02 to 0·15)
Iraq	0·05 (0·02 to 0·09)	0·05 (0·02 to 0·09)	0·05 (-0·03 to 0·12)	0·12 (-0·04 to 0·26)	0·13 (-0·08 to 0·33)	0·13 (0·03 to 0·22)
Jordan	0·01 (0 to 0·02)	0·01 (0 to 0·02)	0·01 (-0·01 to 0·03)	0·03 (-0·01 to 0·08)	0·03 (-0·02 to 0·1)	0·03 (0·01 to 0·06)
Kuwait	0·06 (0·02 to 0·1)	0·07 (0·02 to 0·11)	0·06 (-0·03 to 0·14)	0·15 (-0·05 to 0·32)	0·16 (-0·11 to 0·39)	0·16 (0·04 to 0·27)
Lebanon	0·03 (0·01 to 0·05)	0·04 (0·01 to 0·06)	0·03 (-0·01 to 0·07)	0·09 (-0·03 to 0·2)	0·09 (-0·06 to 0·26)	0·09 (0·02 to 0·16)
Libya	0·04 (0·01 to 0·06)	0·04 (0·01 to 0·06)	0·04 (-0·02 to 0·08)	0·09 (-0·03 to 0·2)	0·09 (-0·06 to 0·26)	0·09 (0·02 to 0·16)
Morocco	0·03 (0·01 to 0·04)	0·03 (0·01 to 0·05)	0·03 (-0·01 to 0·06)	0·07 (-0·02 to 0·15)	0·07 (-0·04 to 0·2)	0·07 (0·02 to 0·12)

Oman	0·03 (0·01 to 0·06)	0·03 (0·01 to 0·06)	0·03 (-0·02 to 0·08)	0·08 (-0·02 to 0·19)	0·08 (-0·05 to 0·24)	0·08 (0·02 to 0·15)
Pakistan	0·04 (0·01 to 0·06)	0·04 (0·01 to 0·06)	0·04 (-0·02 to 0·09)	0·08 (-0·02 to 0·19)	0·09 (-0·05 to 0·24)	0·09 (0·02 to 0·15)
Qatar	0·04 (0·01 to 0·07)	0·04 (0·01 to 0·07)	0·04 (-0·02 to 0·1)	0·09 (-0·03 to 0·2)	0·09 (-0·06 to 0·26)	0·09 (0·02 to 0·17)
Saudi Arabia	0·06 (0·02 to 0·09)	0·06 (0·02 to 0·09)	0·06 (-0·03 to 0·13)	0·13 (-0·04 to 0·27)	0·13 (-0·09 to 0·34)	0·13 (0·03 to 0·22)
Somalia	0·04 (0·01 to 0·06)	0·04 (0·01 to 0·07)	0·04 (-0·02 to 0·09)	0·1 (-0·03 to 0·21)	0·1 (-0·06 to 0·27)	0·1 (0·02 to 0·18)
Sudan	0·04 (0·01 to 0·06)	0·04 (0·01 to 0·07)	0·04 (-0·02 to 0·09)	0·1 (-0·03 to 0·21)	0·1 (-0·06 to 0·27)	0·1 (0·02 to 0·18)
Syrian Arab Republic	0·04 (0·01 to 0·06)	0·04 (0·01 to 0·07)	0·04 (-0·02 to 0·09)	0·1 (-0·03 to 0·21)	0·1 (-0·06 to 0·27)	0·1 (0·02 to 0·18)
Tunisia	0·03 (0·01 to 0·05)	0·03 (0·01 to 0·05)	0·03 (-0·01 to 0·07)	0·08 (-0·02 to 0·17)	0·08 (-0·05 to 0·23)	0·08 (0·02 to 0·14)
United Arab Emirates	0·04 (0·01 to 0·07)	0·04 (0·01 to 0·07)	0·04 (-0·02 to 0·1)	0·1 (-0·03 to 0·22)	0·1 (-0·07 to 0·28)	0·1 (0·02 to 0·18)
Yemen	*	*	*	*	*	*
Djibouti	0·04 (0·01 to 0·06)	0·04 (0·01 to 0·07)	0·04 (-0·02 to 0·09)	0·1 (-0·03 to 0·21)	0·1 (-0·06 to 0·27)	0·1 (0·02 to 0·18)
**European Region**						
Albania	0·03 (0·01 to 0·05)	0·03 (0·01 to 0·05)	0·03 (-0·01 to 0·07)	0·07 (-0·02 to 0·17)	0·08 (-0·05 to 0·22)	0·08 (0·02 to 0·14)
Andorra	*	*	*	*	*	*
Armenia	0·02 (0·01 to 0·03)	0·02 (0·01 to 0·04)	0·02 (-0·01 to 0·05)	0·06 (-0·02 to 0·13)	0·06 (-0·04 to 0·18)	0·06 (0·01 to 0·11)
Austria	0·03 (0·01 to 0·05)	0·03 (0·01 to 0·05)	0·03 (-0·01 to 0·07)	0·08 (-0·02 to 0·17)	0·08 (-0·05 to 0·22)	0·08 (0·02 to 0·14)
Azerbaijan	0·03 (0·01 to 0·05)	0·03 (0·01 to 0·05)	0·03 (-0·01 to 0·07)	0·07 (-0·02 to 0·17)	0·08 (-0·05 to 0·22)	0·08 (0·02 to 0·14)
Belarus	0·01 (0 to 0·02)	0·02 (0 to 0·03)	0·01 (-0·01 to 0·03)	0·04 (-0·01 to 0·09)	0·04 (-0·02 to 0·12)	0·04 (0·01 to 0·07)
Belgium	0·04 (0·01 to 0·06)	0·04 (0·01 to 0·06)	0·04 (-0·02 to 0·08)	0·09 (-0·03 to 0·2)	0·09 (-0·06 to 0·26)	0·09 (0·02 to 0·16)
Bosnia and Herzegovina	0·02 (0·01 to 0·04)	0·03 (0·01 to 0·05)	0·02 (-0·01 to 0·06)	0·06 (-0·02 to 0·15)	0·07 (-0·04 to 0·2)	0·07 (0·02 to 0·12)
Bulgaria	0·04 (0·01 to 0·06)	0·04 (0·01 to 0·07)	0·04 (-0·02 to 0·08)	0·09 (-0·03 to 0·21)	0·1 (-0·06 to 0·27)	0·1 (0·02 to 0·17)
Croatia	0·03 (0·01 to 0·05)	0·03 (0·01 to 0·06)	0·03 (-0·01 to 0·07)	0·08 (-0·02 to 0·18)	0·08 (-0·05 to 0·23)	0·08 (0·02 to 0·14)
Cyprus	0·04 (0·01 to 0·07)	0·05 (0·01 to 0·08)	0·04 (-0·02 to 0·1)	0·11 (-0·03 to 0·23)	0·11 (-0·07 to 0·3)	0·11 (0·03 to 0·19)
Czechia	0·03 (0·01 to 0·05)	0·03 (0·01 to 0·06)	0·03 (-0·01 to 0·07)	0·08 (-0·02 to 0·18)	0·08 (-0·05 to 0·23)	0·08 (0·02 to 0·14)
Denmark	0·03 (0·01 to 0·04)	0·03 (0·01 to 0·05)	0·03 (-0·01 to 0·06)	0·07 (-0·02 to 0·16)	0·07 (-0·04 to 0·21)	0·07 (0·02 to 0·13)
Estonia	0·03 (0·01 to 0·05)	0·03 (0·01 to 0·06)	0·03 (-0·01 to 0·07)	0·08 (-0·02 to 0·18)	0·08 (-0·05 to 0·24)	0·08 (0·02 to 0·15)
Finland	0·01 (0 to 0·02)	0·02 (0 to 0·03)	0·01 (-0·01 to 0·03)	0·04 (-0·01 to 0·1)	0·04 (-0·03 to 0·14)	0·04 (0·01 to 0·08)
France	0·03 (0·01 to 0·05)	0·03 (0·01 to 0·05)	0·03 (-0·01 to 0·07)	0·07 (-0·02 to 0·17)	0·08 (-0·05 to 0·22)	0·08 (0·02 to 0·14)
Georgia	0·02 (0·01 to 0·03)	0·02 (0·01 to 0·03)	0·02 (-0·01 to 0·04)	0·05 (-0·01 to 0·11)	0·05 (-0·03 to 0·15)	0·05 (0·01 to 0·09)
Germany	0·04 (0·01 to 0·06)	0·04 (0·01 to 0·07)	0·04 (-0·02 to 0·09)	0·1 (-0·03 to 0·23)	0·11 (-0·07 to 0·29)	0·11 (0·02 to 0·19)
Greece	0·04 (0·01 to 0·06)	0·04 (0·01 to 0·07)	0·04 (-0·02 to 0·08)	0·09 (-0·03 to 0·21)	0·1 (-0·06 to 0·27)	0·1 (0·02 to 0·17)
Hungary	0·04 (0·01 to 0·06)	0·04 (0·01 to 0·07)	0·04 (-0·02 to 0·09)	0·09 (-0·03 to 0·21)	0·1 (-0·06 to 0·27)	0·1 (0·02 to 0·17)

Iceland	0·03 (0·01 to 0·05)	0·03 (0·01 to 0·05)	0·03 (-0·01 to 0·07)	0·07 (-0·02 to 0·17)	0·08 (-0·05 to 0·22)	0·08 (0·02 to 0·14)
Ireland	0·03 (0·01 to 0·05)	0·03 (0·01 to 0·06)	0·03 (-0·02 to 0·08)	0·08 (-0·02 to 0·18)	0·08 (-0·05 to 0·24)	0·08 (0·02 to 0·15)
Israel	0·03 (0·01 to 0·05)	0·03 (0·01 to 0·05)	0·03 (-0·01 to 0·07)	0·07 (-0·02 to 0·17)	0·08 (-0·05 to 0·22)	0·08 (0·02 to 0·14)
Italy	0·04 (0·01 to 0·06)	0·04 (0·01 to 0·07)	0·04 (-0·02 to 0·09)	0·1 (-0·03 to 0·22)	0·1 (-0·07 to 0·28)	0·1 (0·02 to 0·18)
Kazakhstan	0·03 (0·01 to 0·04)	0·03 (0·01 to 0·05)	0·03 (-0·01 to 0·06)	0·07 (-0·02 to 0·16)	0·07 (-0·04 to 0·21)	0·07 (0·02 to 0·13)
Kyrgyzstan	0·01 (0 to 0·02)	0·02 (0 to 0·03)	0·01 (-0·01 to 0·04)	0·04 (-0·01 to 0·09)	0·04 (-0·02 to 0·12)	0·04 (0·01 to 0·07)
Latvia	0·03 (0·01 to 0·05)	0·03 (0·01 to 0·05)	0·03 (-0·01 to 0·07)	0·07 (-0·02 to 0·17)	0·08 (-0·05 to 0·22)	0·08 (0·02 to 0·14)
Lithuania	0·03 (0·01 to 0·04)	0·03 (0·01 to 0·05)	0·03 (-0·01 to 0·06)	0·07 (-0·02 to 0·15)	0·07 (-0·04 to 0·2)	0·07 (0·02 to 0·13)
Luxembourg	0·03 (0·01 to 0·04)	0·03 (0·01 to 0·05)	0·03 (-0·01 to 0·06)	0·07 (-0·02 to 0·16)	0·07 (-0·04 to 0·21)	0·07 (0·02 to 0·13)
Malta	0·04 (0·01 to 0·07)	0·04 (0·01 to 0·07)	0·04 (-0·02 to 0·09)	0·1 (-0·03 to 0·22)	0·1 (-0·07 to 0·29)	0·1 (0·02 to 0·18)
Monaco	*	*	*	*	*	*
Montenegro	0·03 (0·01 to 0·05)	0·03 (0·01 to 0·05)	0·03 (-0·01 to 0·07)	0·07 (-0·02 to 0·17)	0·08 (-0·05 to 0·22)	0·08 (0·02 to 0·14)
Netherlands	0·03 (0·01 to 0·04)	0·03 (0·01 to 0·05)	0·03 (-0·01 to 0·06)	0·07 (-0·02 to 0·16)	0·07 (-0·04 to 0·21)	0·07 (0·02 to 0·13)
North Macedonia	0·03 (0·01 to 0·05)	0·03 (0·01 to 0·05)	0·03 (-0·01 to 0·07)	0·07 (-0·02 to 0·17)	0·08 (-0·05 to 0·22)	0·08 (0·02 to 0·14)
Norway	0·03 (0·01 to 0·05)	0·03 (0·01 to 0·06)	0·03 (-0·01 to 0·07)	0·08 (-0·02 to 0·18)	0·08 (-0·05 to 0·23)	0·08 (0·02 to 0·15)
Poland	0·03 (0·01 to 0·05)	0·03 (0·01 to 0·06)	0·03 (-0·01 to 0·07)	0·08 (-0·02 to 0·18)	0·08 (-0·05 to 0·24)	0·08 (0·02 to 0·15)
Portugal	0·04 (0·01 to 0·07)	0·05 (0·01 to 0·08)	0·04 (-0·02 to 0·1)	0·1 (-0·03 to 0·23)	0·11 (-0·07 to 0·29)	0·11 (0·03 to 0·19)
Republic of Moldova	0·01 (0 to 0·02)	0·01 (0 to 0·02)	0·01 (0 to 0·02)	0·03 (-0·01 to 0·07)	0·03 (-0·02 to 0·1)	0·03 (0·01 to 0·06)
Romania	0·03 (0·01 to 0·05)	0·04 (0·01 to 0·06)	0·03 (-0·02 to 0·08)	0·09 (-0·03 to 0·2)	0·09 (-0·06 to 0·25)	0·09 (0·02 to 0·16)
Russian Federation	0·02 (0·01 to 0·03)	0·02 (0·01 to 0·03)	0·02 (-0·01 to 0·04)	0·04 (-0·01 to 0·11)	0·05 (-0·03 to 0·14)	0·05 (0·01 to 0·08)
San Marino	*	*	*	*	*	*
Serbia	0·04 (0·01 to 0·06)	0·04 (0·01 to 0·07)	0·04 (-0·02 to 0·09)	0·1 (-0·03 to 0·21)	0·1 (-0·06 to 0·27)	0·1 (0·02 to 0·18)
Slovakia	0·03 (0·01 to 0·05)	0·04 (0·01 to 0·06)	0·03 (-0·02 to 0·08)	0·09 (-0·03 to 0·19)	0·09 (-0·06 to 0·25)	0·09 (0·02 to 0·16)
Slovenia	0·03 (0·01 to 0·05)	0·03 (0·01 to 0·06)	0·03 (-0·01 to 0·07)	0·08 (-0·02 to 0·18)	0·08 (-0·05 to 0·24)	0·08 (0·02 to 0·15)
Spain	0·03 (0·01 to 0·04)	0·03 (0·01 to 0·05)	0·03 (-0·01 to 0·06)	0·07 (-0·02 to 0·16)	0·07 (-0·04 to 0·2)	0·07 (0·02 to 0·13)
Sweden	0·02 (0·01 to 0·04)	0·02 (0·01 to 0·04)	0·02 (-0·01 to 0·05)	0·06 (-0·02 to 0·14)	0·06 (-0·04 to 0·18)	0·06 (0·01 to 0·11)
Switzerland	0·02 (0·01 to 0·04)	0·03 (0·01 to 0·04)	0·02 (-0·01 to 0·05)	0·06 (-0·02 to 0·14)	0·06 (-0·04 to 0·19)	0·06 (0·01 to 0·11)
Tajikistan	0·03 (0·01 to 0·05)	0·03 (0·01 to 0·05)	0·03 (-0·02 to 0·08)	0·07 (-0·02 to 0·17)	0·08 (-0·05 to 0·22)	0·08 (0·02 to 0·14)
Turkey	0·03 (0·01 to 0·05)	0·03 (0·01 to 0·05)	0·03 (-0·02 to 0·08)	0·08 (-0·02 to 0·17)	0·08 (-0·05 to 0·23)	0·08 (0·02 to 0·14)
Turkmenistan	0·03 (0·01 to 0·05)	0·03 (0·01 to 0·05)	0·03 (-0·01 to 0·07)	0·07 (-0·02 to 0·17)	0·08 (-0·05 to 0·22)	0·08 (0·02 to 0·14)
Ukraine	0·02 (0·01 to 0·03)	0·02 (0·01 to 0·04)	0·02 (-0·01 to 0·04)	0·05 (-0·01 to 0·12)	0·05 (-0·03 to 0·16)	0·05 (0·01 to 0·1)
United Kingdom of Great Britain and Northern Ireland (the)	0·03 (0·01 to 0·06)	0·04 (0·01 to 0·06)	0·03 (-0·02 to 0·08)	0·09 (-0·03 to 0·2)	0·09 (-0·06 to 0·26)	0·09 (0·02 to 0·16)

Uzbekistan	0·02 (0·01 to 0·04)	0·02 (0·01 to 0·04)	0·02 (-0·01 to 0·05)	0·05 (-0·01 to 0·12)	0·05 (-0·03 to 0·15)	0·05 (0·01 to 0·09)
**South-East Asian Region**						
Bangladesh	0·03 (0·01 to 0·06)	0·03 (0·01 to 0·05)	0·03 (-0·02 to 0·08)	0·07 (-0·02 to 0·16)	0·07 (-0·04 to 0·21)	0·07 (0·02 to 0·13)
Bhutan	*	*	*	*	*	*
Democratic People's Republic of Korea	0·03 (0·01 to 0·05)	0·02 (0·01 to 0·04)	0·03 (-0·02 to 0·08)	0·06 (-0·02 to 0·13)	0·06 (-0·03 to 0·18)	0·06 (0·01 to 0·11)
India	0·04 (0·01 to 0·06)	0·04 (0·01 to 0·06)	0·04 (-0·02 to 0·09)	0·08 (-0·02 to 0·19)	0·09 (-0·05 to 0·25)	0·09 (0·02 to 0·16)
Indonesia	0·02 (0·01 to 0·03)	0·02 (0·01 to 0·04)	0·02 (-0·01 to 0·05)	0·06 (-0·02 to 0·13)	0·06 (-0·04 to 0·18)	0·06 (0·01 to 0·11)
Maldives	0·03 (0·01 to 0·05)	0·03 (0·01 to 0·05)	0·03 (-0·01 to 0·07)	*	*	*
Myanmar	0·01 (0 to 0·02)	0·01 (0 to 0·02)	0·01 (-0·01 to 0·03)	0·03 (-0·01 to 0·07)	0·03 (-0·02 to 0·09)	0·03 (0·01 to 0·05)
Nepal	0·01 (0 to 0·02)	0·01 (0 to 0·02)	0·01 (-0·01 to 0·03)	0·03 (-0·01 to 0·08)	0·04 (-0·02 to 0·11)	0·04 (0·01 to 0·07)
Sri Lanka	0·03 (0·01 to 0·05)	0·03 (0·01 to 0·05)	0·03 (-0·01 to 0·07)	0·07 (-0·02 to 0·17)	0·07 (-0·05 to 0·22)	0·07 (0·02 to 0·13)
Thailand	0·02 (0·01 to 0·04)	0·03 (0·01 to 0·04)	0·02 (-0·01 to 0·06)	0·06 (-0·02 to 0·15)	0·06 (-0·04 to 0·19)	0·06 (0·01 to 0·12)
Timor-Leste	0·02 (0·01 to 0·04)	0·02 (0·01 to 0·03)	0·02 (-0·01 to 0·05)	0·05 (-0·01 to 0·11)	0·05 (-0·03 to 0·15)	0·05 (0·01 to 0·09)
**Western Pacific Region**						
Australia	0·03 (0·01 to 0·05)	0·03 (0·01 to 0·04)	0·03 (-0·01 to 0·07)	0·08 (-0·02 to 0·17)	0·08 (-0·05 to 0·23)	0·08 (0·02 to 0·14)
Brunei Darussalam	0·03 (0·01 to 0·05)	0·03 (0·01 to 0·04)	0·03 (-0·01 to 0·07)	0·07 (-0·02 to 0·16)	0·07 (-0·04 to 0·21)	0·07 (0·02 to 0·13)
Cambodia	0·01 (0 to 0·02)	0·01 (0 to 0·02)	0·01 (0 to 0·02)	0·03 (-0·01 to 0·07)	0·03 (-0·02 to 0·09)	0·03 (0·01 to 0·05)
China	0·01 (0 to 0·02)	0·02 (0 to 0·02)	0·01 (0 to 0·03)	0·04 (-0·01 to 0·09)	0·04 (-0·02 to 0·12)	0·04 (0·01 to 0·07)
Cook Islands	*	*	*	*	*	*
Fiji	*	0·02 (0·01 to 0·03)	0·02 (-0·01 to 0·05)	0·04 (-0·01 to 0·11)	0·05 (-0·03 to 0·14)	0·05 (0·01 to 0·09)
Japan	0·03 (0·01 to 0·05)	0·04 (0·01 to 0·05)	0·03 (-0·02 to 0·08)	0·09 (-0·03 to 0·2)	0·09 (-0·06 to 0·25)	0·09 (0·02 to 0·16)
Kiribati	*	*	*	*	*	*
Lao People's Democratic Republic	0·02 (0·01 to 0·03)	0·02 (0 to 0·02)	0·02 (-0·01 to 0·04)	0·04 (-0·01 to 0·1)	0·04 (-0·03 to 0·14)	0·04 (0·01 to 0·08)
Malaysia	0·04 (0·01 to 0·06)	0·04 (0·01 to 0·05)	0·04 (-0·02 to 0·09)	0·09 (-0·03 to 0·21)	0·1 (-0·06 to 0·27)	0·1 (0·02 to 0·17)
Marshall Islands	*	*	*	*	*	*
Micronesia (Federated States of)	0·04 (0·01 to 0·06)	0·04 (0·01 to 0·05)	0·04 (-0·02 to 0·08)	0·09 (-0·03 to 0·2)	0·09 (-0·06 to 0·26)	0·09 (0·02 to 0·17)
Mongolia	0·02 (0·01 to 0·03)	0·02 (0·01 to 0·03)	0·02 (-0·01 to 0·04)	0·05 (-0·01 to 0·11)	0·05 (-0·03 to 0·15)	0·05 (0·01 to 0·09)
Nauru	*	*	*	*	*	*
New Zealand	0·04 (0·01 to 0·06)	0·04 (0·01 to 0·06)	0·04 (-0·02 to 0·09)	0·1 (-0·03 to 0·23)	0·11 (-0·07 to 0·29)	0·11 (0·02 to 0·19)
Niue	*	*	*	*	*	*
Palau	*	*	*	*	*	*
Papua New Guinea	0·02 (0·01 to 0·03)	0·02 (0 to 0·02)	0·02 (-0·01 to 0·04)	0·04 (-0·01 to 0·09)	0·04 (-0·02 to 0·12)	0·04 (0·01 to 0·07)

Philippines	0·04 (0·01 to 0·07)	0·04 (0·01 to 0·06)	0·04 (-0·02 to 0·1)	0·1 (-0·03 to 0·22)	0·1 (-0·06 to 0·28)	0·1 (0·02 to 0·18)
Republic of Korea	0·04 (0·01 to 0·06)	0·04 (0·01 to 0·05)	0·04 (-0·02 to 0·08)	0·09 (-0·03 to 0·2)	0·09 (-0·06 to 0·25)	0·09 (0·02 to 0·16)
Samoa	0·02 (0·01 to 0·03)	0·01 (0 to 0·02)	0·02 (-0·01 to 0·04)	0·03 (-0·01 to 0·08)	0·03 (-0·02 to 0·11)	0·03 (0·01 to 0·06)
Singapore	0·03 (0·01 to 0·05)	0·04 (0·01 to 0·05)	0·03 (-0·02 to 0·08)	0·09 (-0·03 to 0·2)	0·09 (-0·06 to 0·26)	0·09 (0·02 to 0·16)
Solomon Islands	0·02 (0·01 to 0·03)	0·02 (0·01 to 0·03)	0·02 (-0·01 to 0·05)	0·05 (-0·01 to 0·11)	0·05 (-0·03 to 0·15)	*
Tonga	*	*	*	*	*	*
Tuvalu	*	*	*	*	*	*
Vanuatu	0·01 (0 to 0·01)	0·01 (0 to 0·01)	0·01 (0 to 0·02)	0·02 (-0·01 to 0·05)	*	*
Viet Nam	0·03 (0·01 to 0·04)	0·03 (0·01 to 0·04)	0·03 (-0·01 to 0·06)	0·06 (-0·02 to 0·15)	0·07 (-0·04 to 0·2)	0·07 (0·02 to 0·12)

*No data available

(continued from previous table)

**African Region**	**Bladder cancer**	**Stroke**	**Coronary Heart Disease**	**Type 2 diabetes**
Algeria	0·03 (-0·02 to 0·08)	0·06 (0·03 to 0·09)	0·06 (0·04 to 0·08)	0·05 (0·04 to 0·07)
Angola	0·01 (-0·01 to 0·04)	0·03 (0·02 to 0·05)	0·03 (0·02 to 0·05)	0·03 (0·02 to 0·04)
Benin	0·01 (-0·01 to 0·04)	0·03 (0·01 to 0·04)	0·03 (0·02 to 0·04)	0·03 (0·02 to 0·04)
Botswana	0·02 (-0·02 to 0·05)	0·04 (0·02 to 0·06)	0·04 (0·03 to 0·05)	0·04 (0·02 to 0·05)
Burkina Faso	0·02 (-0·01 to 0·05)	0·04 (0·02 to 0·05)	0·04 (0·03 to 0·05)	0·03 (0·02 to 0·04)
Burundi	0·01 (-0·01 to 0·04)	0·03 (0·02 to 0·05)	0·03 (0·02 to 0·05)	0·03 (0·02 to 0·04)
Cabo Verde	0·02 (-0·01 to 0·05)	0·04 (0·02 to 0·05)	0·04 (0·02 to 0·05)	0·03 (0·02 to 0·04)
Cameroon	0·02 (-0·02 to 0·07)	0·05 (0·03 to 0·07)	0·05 (0·04 to 0·07)	0·05 (0·03 to 0·06)
Central Africa Republic	0·01 (-0·01 to 0·03)	0·03 (0·01 to 0·04)	0·03 (0·02 to 0·04)	0·02 (0·02 to 0·03)
Chad	0·02 (-0·02 to 0·06)	0·04 (0·02 to 0·06)	0·04 (0·03 to 0·06)	0·04 (0·02 to 0·05)
Comoros (The)	0·01 (-0·01 to 0·03)	0·03 (0·01 to 0·04)	0·03 (0·02 to 0·04)	0·02 (0·02 to 0·03)
Congo	0·02 (-0·02 to 0·07)	0·05 (0·02 to 0·07)	0·05 (0·04 to 0·07)	0·05 (0·03 to 0·06)
Côte d'Ivoire	0·03 (-0·02 to 0·08)	0·06 (0·03 to 0·08)	0·06 (0·04 to 0·08)	0·05 (0·04 to 0·07)
Democratic Republic of the Congo	0·02 (-0·02 to 0·06)	0·04 (0·02 to 0·06)	0·04 (0·03 to 0·06)	0·04 (0·03 to 0·05)
Equatorial Guinea	0·01 (-0·01 to 0·04)	0·03 (0·02 to 0·05)	0·03 (0·02 to 0·05)	0·03 (0·02 to 0·04)
Eritrea	0·02 (-0·02 to 0·05)	0·04 (0·02 to 0·06)	0·04 (0·03 to 0·06)	0·04 (0·02 to 0·05)
Eswatini	0·02 (-0·02 to 0·07)	0·05 (0·02 to 0·07)	0·05 (0·04 to 0·07)	0·05 (0·03 to 0·06)
Ethiopia	0·01 (-0·01 to 0·04)	0·03 (0·01 to 0·04)	0·03 (0·02 to 0·04)	0·02 (0·02 to 0·03)
Gabon	0·02 (-0·02 to 0·06)	0·05 (0·02 to 0·07)	0·05 (0·03 to 0·06)	0·04 (0·03 to 0·05)

Ghana	0·02 (-0·02 to 0·05)	0·04 (0·02 to 0·06)	0·04 (0·03 to 0·05)	0·04 (0·02 to 0·05)
Guinea	0·01 (-0·01 to 0·03)	0·03 (0·01 to 0·04)	0·03 (0·02 to 0·04)	0·02 (0·02 to 0·03)
Guinea-Bissau	0·01 (-0·01 to 0·04)	0·03 (0·02 to 0·05)	0·03 (0·02 to 0·05)	0·03 (0·02 to 0·04)
Kenya	0·01 (-0·01 to 0·04)	0·03 (0·01 to 0·04)	0·03 (0·02 to 0·04)	0·03 (0·02 to 0·03)
Lesotho	0·01 (0 to 0·02)	0·01 (0·01 to 0·02)	0·01 (0·01 to 0·02)	0·01 (0·01 to 0·01)
Liberia	0·02 (-0·02 to 0·06)	0·05 (0·02 to 0·07)	0·05 (0·03 to 0·06)	0·04 (0·03 to 0·05)
Madagascar	0·01 (-0·01 to 0·04)	0·03 (0·02 to 0·05)	0·03 (0·02 to 0·04)	0·03 (0·02 to 0·04)
Malawi	0·01 (-0·01 to 0·04)	0·03 (0·01 to 0·04)	0·03 (0·02 to 0·04)	0·03 (0·02 to 0·03)
Mali	0·03 (-0·03 to 0·09)	0·07 (0·04 to 0·1)	0·07 (0·05 to 0·1)	0·06 (0·04 to 0·09)
Mauritania	0·03 (-0·03 to 0·09)	0·07 (0·04 to 0·1)	0·07 (0·05 to 0·1)	0·07 (0·04 to 0·09)
Mauritius	0·02 (-0·02 to 0·07)	0·05 (0·03 to 0·08)	0·05 (0·04 to 0·07)	0·05 (0·03 to 0·06)
Mozambique	0 (0 to 0·01)	0·01 (0·01 to 0·02)	0·01 (0·01 to 0·01)	0·01 (0·01 to 0·01)
Namibia	0·03 (-0·02 to 0·08)	0·06 (0·03 to 0·09)	0·06 (0·04 to 0·08)	0·05 (0·04 to 0·07)
Niger	0·02 (-0·02 to 0·05)	0·04 (0·02 to 0·06)	0·04 (0·03 to 0·06)	0·04 (0·02 to 0·05)
Nigeria	0·02 (-0·02 to 0·06)	0·05 (0·02 to 0·07)	0·05 (0·03 to 0·07)	0·04 (0·03 to 0·06)
Rwanda	0·01 (-0·01 to 0·04)	0·03 (0·01 to 0·04)	0·03 (0·02 to 0·04)	0·02 (0·02 to 0·03)
Sao Tome and Principe	0·01 (-0·01 to 0·04)	0·03 (0·01 to 0·04)	0·03 (0·02 to 0·04)	0·03 (0·02 to 0·03)
Senegal	0·02 (-0·02 to 0·05)	0·04 (0·02 to 0·06)	0·04 (0·03 to 0·06)	0·04 (0·02 to 0·05)
Seychelles	*	0·03 (0·02 to 0·05)	0·03 (0·02 to 0·05)	0·03 (0·02 to 0·04)
Sierra Leone	0·01 (-0·01 to 0·03)	0·03 (0·01 to 0·04)	0·03 (0·02 to 0·04)	0·02 (0·02 to 0·03)
South Africa	0·03 (-0·03 to 0·09)	0·07 (0·03 to 0·1)	0·07 (0·05 to 0·09)	0·06 (0·04 to 0·08)
South Sudan	0·01 (-0·01 to 0·04)	0·03 (0·02 to 0·05)	0·03 (0·02 to 0·05)	0·03 (0·02 to 0·04)
the Gambia	0·02 (-0·01 to 0·05)	0·04 (0·02 to 0·06)	0·04 (0·03 to 0·05)	0·03 (0·02 to 0·05)
Togo	0·01 (-0·01 to 0·02)	0·02 (0·01 to 0·03)	0·02 (0·01 to 0·02)	0·02 (0·01 to 0·02)
Uganda	0 (0 to 0·01)	0·01 (0 to 0·02)	0·01 (0·01 to 0·01)	0·01 (0·01 to 0·01)
United Republic of Tanzania	0·01 (0 to 0·02)	0·01 (0·01 to 0·02)	0·01 (0·01 to 0·02)	0·01 (0·01 to 0·01)
Zambia	*	0·04 (0·02 to 0·06)	0·04 (0·03 to 0·05)	0·04 (0·02 to 0·05)
Zimbabwe	*	0·05 (0·02 to 0·07)	0·05 (0·03 to 0·07)	0·04 (0·03 to 0·06)
**Region of the Americas**				
Antigua and Barbuda	*	0·06 (0·03 to 0·09)	0·06 (0·04 to 0·08)	0·06 (0·04 to 0·07)
Argentina	0·03 (-0·03 to 0·09)	0·07 (0·04 to 0·1)	0·07 (0·05 to 0·1)	0·07 (0·04 to 0·09)
Bahamas (The)	0·03 (-0·03 to 0·1)	0·08 (0·04 to 0·11)	0·08 (0·05 to 0·1)	0·07 (0·05 to 0·09)

Barbados	0·03 (-0·03 to 0·1)	0·08 (0·04 to 0·11)	0·08 (0·05 to 0·1)	0·07 (0·05 to 0·09)
Belize	*	0·06 (0·03 to 0·09)	0·06 (0·04 to 0·08)	0·06 (0·04 to 0·07)
Bolivia (Plurinational State of)	0·03 (-0·03 to 0·08)	0·06 (0·03 to 0·09)	0·06 (0·04 to 0·08)	0·06 (0·04 to 0·07)
Brazil	0·04 (-0·03 to 0·11)	0·08 (0·04 to 0·12)	0·08 (0·06 to 0·11)	0·07 (0·05 to 0·1)
Canada	0·02 (-0·02 to 0·07)	0·05 (0·03 to 0·07)	0·05 (0·04 to 0·07)	0·05 (0·03 to 0·06)
Chile	0·02 (-0·02 to 0·06)	0·05 (0·02 to 0·07)	0·05 (0·03 to 0·06)	0·04 (0·03 to 0·06)
Colombia	0·03 (-0·03 to 0·1)	0·08 (0·04 to 0·11)	0·08 (0·05 to 0·1)	0·07 (0·05 to 0·09)
Costa Rica	0·04 (-0·03 to 0·1)	0·08 (0·04 to 0·11)	0·08 (0·06 to 0·11)	0·07 (0·05 to 0·1)
Cuba	0·03 (-0·03 to 0·08)	0·07 (0·03 to 0·09)	0·07 (0·05 to 0·09)	0·06 (0·04 to 0·08)
Dominica	*	0·08 (0·04 to 0·11)	0·08 (0·05 to 0·1)	0·07 (0·05 to 0·09)
Dominican Republic (The)	0·03 (-0·03 to 0·09)	0·07 (0·03 to 0·1)	0·07 (0·05 to 0·09)	0·06 (0·04 to 0·08)
Ecuador	0·02 (-0·02 to 0·06)	0·05 (0·02 to 0·07)	0·05 (0·03 to 0·07)	0·04 (0·03 to 0·06)
El Salvador	0·03 (-0·03 to 0·08)	0·06 (0·03 to 0·09)	0·06 (0·04 to 0·08)	0·06 (0·04 to 0·07)
Grenada	*	0·05 (0·03 to 0·07)	0·05 (0·04 to 0·07)	0·05 (0·03 to 0·06)
Guatemala	0·03 (-0·03 to 0·08)	0·07 (0·03 to 0·09)	0·07 (0·05 to 0·09)	0·06 (0·04 to 0·08)
Guyana	0·03 (-0·03 to 0·08)	0·06 (0·03 to 0·09)	0·06 (0·04 to 0·08)	0·06 (0·04 to 0·07)
Haiti	0·03 (-0·03 to 0·08)	0·06 (0·03 to 0·09)	0·06 (0·04 to 0·08)	0·06 (0·04 to 0·07)
Honduras	0·03 (-0·03 to 0·08)	0·06 (0·03 to 0·09)	0·06 (0·04 to 0·08)	0·06 (0·04 to 0·07)
Jamaica	0·03 (-0·02 to 0·08)	0·06 (0·03 to 0·08)	0·06 (0·04 to 0·08)	0·05 (0·03 to 0·07)
Mexico	0·02 (-0·02 to 0·07)	0·05 (0·03 to 0·07)	0·05 (0·04 to 0·07)	0·05 (0·03 to 0·06)
Nicaragua	0·03 (-0·03 to 0·08)	0·06 (0·03 to 0·09)	0·06 (0·04 to 0·08)	0·06 (0·04 to 0·07)
Panama	0·03 (-0·03 to 0·08)	0·06 (0·03 to 0·09)	0·06 (0·04 to 0·08)	0·06 (0·04 to 0·07)
Paraguay	0·03 (-0·03 to 0·09)	0·07 (0·03 to 0·09)	0·07 (0·05 to 0·09)	0·06 (0·04 to 0·08)
Peru	0·03 (-0·03 to 0·08)	0·06 (0·03 to 0·09)	0·06 (0·04 to 0·08)	0·06 (0·04 to 0·07)
Saint Kitts and Nevis	*	0·05 (0·03 to 0·08)	0·06 (0·04 to 0·08)	0·05 (0·04 to 0·07)
Saint Lucia	0·03 (-0·03 to 0·09)	0·07 (0·03 to 0·1)	0·07 (0·05 to 0·09)	0·06 (0·04 to 0·08)
Saint Vicent and the Grenadines	*	0·06 (0·03 to 0·09)	0·06 (0·04 to 0·08)	0·06 (0·04 to 0·07)
Suriname	0·03 (-0·03 to 0·1)	0·08 (0·04 to 0·11)	0·08 (0·05 to 0·1)	0·07 (0·05 to 0·09)
Trinidad and Tobago	0·03 (-0·03 to 0·09)	0·07 (0·03 to 0·1)	0·07 (0·05 to 0·09)	0·06 (0·04 to 0·08)
United States of America	0·03 (-0·03 to 0·09)	0·07 (0·03 to 0·1)	0·07 (0·05 to 0·09)	0·06 (0·04 to 0·08)
Uruguay	0·02 (-0·02 to 0·05)	0·04 (0·02 to 0·06)	0·04 (0·03 to 0·06)	0·04 (0·02 to 0·05)
Venezuela (Bolivarian Republic of)	0·02 (-0·02 to 0·07)	0·06 (0·03 to 0·08)	0·06 (0·04 to 0·08)	0·05 (0·03 to 0·07)

Eastern Mediterran Region				
Afghanistan	0·03 (-0·03 to 0·09)	0·07 (0·03 to 0·1)	0·07 (0·05 to 0·09)	0·06 (0·04 to 0·08)
Bahrain	0·03 (-0·03 to 0·09)	0·07 (0·03 to 0·1)	0·07 (0·05 to 0·09)	0·06 (0·04 to 0·08)
Egypt	0·02 (-0·02 to 0·07)	0·06 (0·03 to 0·08)	0·06 (0·04 to 0·07)	0·05 (0·03 to 0·07)
Iran (Islamic Republic of)	0·03 (-0·02 to 0·08)	0·06 (0·03 to 0·09)	0·06 (0·04 to 0·08)	0·05 (0·04 to 0·07)
Iraq	0·04 (-0·04 to 0·12)	0·09 (0·04 to 0·13)	0·09 (0·06 to 0·12)	0·08 (0·05 to 0·11)
Jordan	0·01 (-0·01 to 0·03)	0·02 (0·01 to 0·03)	0·02 (0·02 to 0·03)	0·02 (0·01 to 0·03)
Kuwait	0·05 (-0·05 to 0·14)	0·11 (0·06 to 0·16)	0·11 (0·08 to 0·15)	0·1 (0·07 to 0·13)
Lebanon	0·03 (-0·03 to 0·08)	0·06 (0·03 to 0·09)	0·06 (0·05 to 0·09)	0·06 (0·04 to 0·08)
Libya	0·03 (-0·03 to 0·08)	0·06 (0·03 to 0·09)	0·06 (0·05 to 0·09)	0·06 (0·04 to 0·08)
Morocco	0·02 (-0·02 to 0·06)	0·05 (0·02 to 0·07)	0·05 (0·03 to 0·06)	0·04 (0·03 to 0·06)
Oman	0·03 (-0·02 to 0·08)	0·06 (0·03 to 0·08)	0·06 (0·04 to 0·08)	0·05 (0·03 to 0·07)
Pakistan	0·03 (-0·02 to 0·08)	0·06 (0·03 to 0·09)	0·06 (0·04 to 0·08)	0·05 (0·04 to 0·07)
Qatar	0·03 (-0·03 to 0·08)	0·07 (0·03 to 0·09)	0·07 (0·05 to 0·09)	0·06 (0·04 to 0·08)
Saudi Arabia	0·04 (-0·04 to 0·12)	0·09 (0·05 to 0·13)	0·09 (0·06 to 0·12)	0·08 (0·06 to 0·11)
Somalia	0·03 (-0·03 to 0·09)	0·07 (0·03 to 0·1)	0·07 (0·05 to 0·09)	0·06 (0·04 to 0·08)
Sudan	0·03 (-0·03 to 0·09)	0·07 (0·03 to 0·1)	0·07 (0·05 to 0·09)	0·06 (0·04 to 0·08)
Syrian Arab Republic	0·03 (-0·03 to 0·09)	0·07 (0·03 to 0·1)	0·07 (0·05 to 0·09)	0·06 (0·04 to 0·08)
Tunisia	0·02 (-0·02 to 0·07)	0·05 (0·03 to 0·08)	0·05 (0·04 to 0·07)	0·05 (0·03 to 0·07)
United Arab Emirates	0·03 (-0·03 to 0·09)	0·07 (0·04 to 0·1)	0·07 (0·05 to 0·1)	0·07 (0·04 to 0·09)
Yemen	*	0·07 (0·03 to 0·1)	0·07 (0·05 to 0·09)	0·06 (0·04 to 0·08)
Djibouti	0·03 (-0·03 to 0·09)	0·07 (0·03 to 0·1)	0·07 (0·05 to 0·09)	0·06 (0·04 to 0·08)
**European Region**				
Albania	0·07 (-0·02 to 0·07)	0·05 (0·03 to 0·08)	0·05 (0·04 to 0·07)	0·05 (0·03 to 0·06)
Andorra	*	0·07 (0·03 to 0·1)	0·07 (0·05 to 0·09)	0·06 (0·04 to 0·08)
Armenia	0·05 (-0·02 to 0·05)	0·04 (0·02 to 0·06)	0·04 (0·03 to 0·06)	0·04 (0·02 to 0·05)
Austria	0·07 (-0·02 to 0·07)	0·05 (0·03 to 0·08)	0·05 (0·04 to 0·07)	0·05 (0·03 to 0·06)
Azerbaijan	0·07 (-0·02 to 0·07)	0·05 (0·03 to 0·08)	0·05 (0·04 to 0·07)	0·05 (0·03 to 0·06)
Belarus	0·03 (-0·01 to 0·03)	0·03 (0·01 to 0·04)	0·03 (0·02 to 0·04)	0·02 (0·02 to 0·03)
Belgium	0·08 (-0·03 to 0·08)	0·06 (0·03 to 0·09)	0·06 (0·04 to 0·08)	0·06 (0·04 to 0·08)
Bosnia and Herzegovina	0·06 (-0·02 to 0·06)	0·05 (0·02 to 0·07)	0·05 (0·03 to 0·06)	0·04 (0·03 to 0·06)
Bulgaria	0·09 (-0·03 to 0·09)	0·07 (0·03 to 0·1)	0·07 (0·05 to 0·09)	0·06 (0·04 to 0·08)

Croatia	0·07 (-0·02 to 0·07)	0·06 (0·03 to 0·08)	0·06 (0·04 to 0·07)	0·05 (0·03 to 0·07)
Cyprus	0·1 (-0·03 to 0·1)	0·08 (0·04 to 0·11)	0·08 (0·05 to 0·1)	0·07 (0·05 to 0·09)
Czechia	0·07 (-0·02 to 0·07)	0·06 (0·03 to 0·08)	0·06 (0·04 to 0·07)	0·05 (0·03 to 0·07)
Denmark	0·07 (-0·02 to 0·07)	0·05 (0·03 to 0·07)	0·05 (0·04 to 0·07)	0·05 (0·03 to 0·06)
Estonia	0·07 (-0·02 to 0·07)	0·06 (0·03 to 0·08)	0·06 (0·04 to 0·08)	0·05 (0·03 to 0·07)
Finland	0·04 (-0·01 to 0·04)	0·03 (0·01 to 0·04)	0·03 (0·02 to 0·04)	0·03 (0·02 to 0·04)
France	0·07 (-0·02 to 0·07)	0·05 (0·03 to 0·08)	0·05 (0·04 to 0·07)	0·05 (0·03 to 0·06)
Georgia	0·04 (-0·01 to 0·04)	0·03 (0·02 to 0·05)	0·03 (0·02 to 0·04)	0·03 (0·02 to 0·04)
Germany	0·1 (-0·03 to 0·1)	0·07 (0·04 to 0·11)	0·07 (0·05 to 0·1)	0·07 (0·04 to 0·09)
Greece	0·09 (-0·03 to 0·09)	0·07 (0·03 to 0·1)	0·07 (0·05 to 0·09)	0·06 (0·04 to 0·08)
Hungary	0·09 (-0·03 to 0·09)	0·07 (0·03 to 0·1)	0·07 (0·05 to 0·09)	0·06 (0·04 to 0·08)
Iceland	0·07 (-0·02 to 0·07)	0·05 (0·03 to 0·08)	0·05 (0·04 to 0·07)	0·05 (0·03 to 0·06)
Ireland	0·08 (-0·02 to 0·08)	0·06 (0·03 to 0·08)	0·06 (0·04 to 0·08)	0·05 (0·03 to 0·07)
Israel	0·07 (-0·02 to 0·07)	0·05 (0·03 to 0·08)	0·05 (0·04 to 0·07)	0·05 (0·03 to 0·06)
Italy	0·09 (-0·03 to 0·09)	0·07 (0·04 to 0·1)	0·07 (0·05 to 0·1)	0·07 (0·04 to 0·09)
Kazakhstan	0·06 (-0·02 to 0·06)	0·05 (0·02 to 0·07)	0·05 (0·03 to 0·07)	0·04 (0·03 to 0·06)
Kyrgyzstan	0·03 (-0·01 to 0·03)	0·03 (0·01 to 0·04)	0·03 (0·02 to 0·03)	0·02 (0·02 to 0·03)
Latvia	0·07 (-0·02 to 0·07)	0·05 (0·03 to 0·08)	0·05 (0·04 to 0·07)	0·05 (0·03 to 0·06)
Lithuania	0·06 (-0·02 to 0·06)	0·05 (0·02 to 0·07)	0·05 (0·03 to 0·06)	0·04 (0·03 to 0·06)
Luxembourg	0·07 (-0·02 to 0·07)	0·05 (0·02 to 0·07)	0·05 (0·04 to 0·07)	0·05 (0·03 to 0·06)
Malta	0·09 (-0·03 to 0·09)	0·07 (0·04 to 0·1)	0·07 (0·05 to 0·1)	0·07 (0·04 to 0·09)
Monaco	*	0·05 (0·03 to 0·08)	0·05 (0·04 to 0·07)	0·05 (0·03 to 0·06)
Montenegro	0·07 (-0·02 to 0·07)	0·05 (0·03 to 0·08)	0·05 (0·04 to 0·07)	0·05 (0·03 to 0·06)
Netherlands	0·06 (-0·02 to 0·06)	0·05 (0·02 to 0·07)	0·05 (0·03 to 0·07)	0·04 (0·03 to 0·06)
North Macedonia	0·07 (-0·02 to 0·07)	0·05 (0·03 to 0·08)	0·05 (0·04 to 0·07)	0·05 (0·03 to 0·06)
Norway	0·07 (-0·02 to 0·07)	0·06 (0·03 to 0·08)	0·06 (0·04 to 0·08)	0·05 (0·03 to 0·07)
Poland	0·08 (-0·02 to 0·08)	0·06 (0·03 to 0·08)	0·06 (0·04 to 0·08)	0·05 (0·03 to 0·07)
Portugal	0·1 (-0·03 to 0·1)	0·08 (0·04 to 0·11)	0·08 (0·05 to 0·1)	0·07 (0·05 to 0·09)
Republic of Moldova	0·03 (-0·01 to 0·03)	0·02 (0·01 to 0·03)	0·02 (0·01 to 0·03)	0·02 (0·01 to 0·03)
Romania	0·08 (-0·03 to 0·08)	0·06 (0·03 to 0·09)	0·06 (0·04 to 0·08)	0·06 (0·04 to 0·08)
Russian Federation	0·04 (-0·01 to 0·04)	0·03 (0·02 to 0·05)	0·03 (0·02 to 0·04)	0·03 (0·02 to 0·04)
San Marino	*	0·05 (0·03 to 0·08)	0·05 (0·04 to 0·07)	0·05 (0·03 to 0·06)

Serbia	0·09 (-0·03 to 0·09)	0·07 (0·03 to 0·1)	0·07 (0·05 to 0·09)	0·06 (0·04 to 0·08)
Slovakia	0·08 (-0·03 to 0·08)	0·06 (0·03 to 0·09)	0·06 (0·04 to 0·08)	0·06 (0·04 to 0·07)
Slovenia	0·07 (-0·02 to 0·07)	0·06 (0·03 to 0·08)	0·06 (0·04 to 0·08)	0·05 (0·03 to 0·07)
Spain	0·06 (-0·02 to 0·06)	0·05 (0·02 to 0·07)	0·05 (0·03 to 0·07)	0·04 (0·03 to 0·06)
Sweden	0·05 (-0·02 to 0·05)	0·04 (0·02 to 0·06)	0·04 (0·03 to 0·06)	0·04 (0·02 to 0·05)
Switzerland	0·06 (-0·02 to 0·06)	0·04 (0·02 to 0·06)	0·04 (0·03 to 0·06)	0·04 (0·03 to 0·05)
Tajikistan	0·07 (-0·02 to 0·07)	0·05 (0·03 to 0·08)	0·05 (0·04 to 0·07)	0·05 (0·03 to 0·06)
Turkey	0·07 (-0·02 to 0·07)	0·05 (0·03 to 0·08)	0·05 (0·04 to 0·07)	0·05 (0·03 to 0·07)
Turkmenistan	0·07 (-0·02 to 0·07)	0·05 (0·03 to 0·08)	0·05 (0·04 to 0·07)	0·05 (0·03 to 0·06)
Ukraine	0·05 (-0·01 to 0·05)	0·04 (0·02 to 0·05)	0·04 (0·02 to 0·05)	0·03 (0·02 to 0·04)
United Kingdom of Great Britain and Northern Ireland (the)	0·08 (-0·03 to 0·08)	0·06 (0·03 to 0·09)	0·06 (0·04 to 0·09)	0·06 (0·04 to 0·08)
Uzbekistan	0·05 (-0·01 to 0·05)	0·04 (0·02 to 0·05)	0·04 (0·02 to 0·05)	0·03 (0·02 to 0·04)
**South-East Asian Region**				
Bangladesh	0·02 (-0·02 to 0·06)	0·05 (0·02 to 0·07)	0·05 (0·03 to 0·07)	0·05 (0·03 to 0·06)
Bhutan	*	0·04 (0·02 to 0·06)	0·04 (0·04 to 0·04)	0·04 (0·04 to 0·04)
Democratic People's Republic of Korea	0·02 (-0·02 to 0·05)	0·04 (0·02 to 0·06)	0·04 (0·03 to 0·05)	0·04 (0·02 to 0·05)
India	0·03 (-0·02 to 0·08)	0·06 (0·03 to 0·09)	0·06 (0·04 to 0·08)	0·05 (0·04 to 0·07)
Indonesia	0·02 (-0·02 to 0·05)	0·04 (0·02 to 0·06)	0·04 (0·03 to 0·06)	0·04 (0·02 to 0·05)
Maldives	0·02 (-0·02 to 0·07)	0·05 (0·03 to 0·08)	0·05 (0·04 to 0·07)	0·05 (0·03 to 0·07)
Myanmar	0·01 (-0·01 to 0·03)	0·02 (0·01 to 0·03)	0·02 (0·01 to 0·03)	0·02 (0·01 to 0·02)
Nepal	0·01 (-0·01 to 0·03)	0·02 (0·01 to 0·04)	0·02 (0·02 to 0·03)	0·02 (0·01 to 0·03)
Sri Lanka	0·02 (-0·02 to 0·07)	0·05 (0·03 to 0·07)	0·05 (0·04 to 0·07)	0·05 (0·03 to 0·06)
Thailand	0·02 (-0·02 to 0·06)	0·04 (0·02 to 0·06)	0·04 (0·03 to 0·06)	0·04 (0·03 to 0·05)
Timor-Leste	0·01 (-0·01 to 0·04)	0·03 (0·02 to 0·05)	0·03 (0·02 to 0·04)	0·03 (0·02 to 0·04)
**Western Pacific Region**				
Australia	0·07 (-0·02 to 0·07)	0·05 (0·03 to 0·08)	0·05 (0·04 to 0·07)	0·05 (0·03 to 0·07)
Brunei Darussalam	0·06 (-0·02 to 0·06)	0·05 (0·02 to 0·07)	0·05 (0·03 to 0·07)	0·04 (0·03 to 0·06)
Cambodia	0·03 (-0·01 to 0·03)	0·02 (0·01 to 0·03)	0·02 (0·01 to 0·03)	0·02 (0·01 to 0·02)
China	0·03 (-0·01 to 0·03)	0·03 (0·01 to 0·04)	0·03 (0·02 to 0·04)	0·02 (0·02 to 0·03)
Cook Islands	*	0·03 (0·02 to 0·05)	0·03 (0·02 to 0·05)	0·03 (0·02 to 0·04)
Fiji	0·04 (-0·01 to 0·04)	0·03 (0·02 to 0·05)	0·03 (0·02 to 0·04)	0·03 (0·02 to 0·04)
Japan	0·08 (-0·03 to 0·08)	0·06 (0·03 to 0·09)	0·06 (0·04 to 0·08)	0·06 (0·04 to 0·08)

Kiribati	*	0·07 (0·04 to 0·1)	0·07 (0·05 to 0·1)	0·06 (0·04 to 0·09)
Lao People's Democratic Republic	0·04 (-0·01 to 0·04)	0·03 (0·01 to 0·04)	0·03 (0·02 to 0·04)	0·03 (0·02 to 0·04)
Malaysia	0·09 (-0·03 to 0·09)	0·07 (0·03 to 0·1)	0·07 (0·05 to 0·09)	0·06 (0·04 to 0·08)
Marshall Islands	*	0·08 (0·04 to 0·11)	0·08 (0·05 to 0·1)	0·07 (0·05 to 0·09)
Micronesia (Federated States of)	0·08 (-0·03 to 0·08)	0·07 (0·03 to 0·09)	0·07 (0·05 to 0·09)	0·06 (0·04 to 0·08)
Mongolia	0·04 (-0·01 to 0·04)	0·03 (0·02 to 0·05)	0·03 (0·02 to 0·05)	0·03 (0·02 to 0·04)
Nauru	*	0·07 (0·03 to 0·1)	0·07 (0·05 to 0·09)	0·06 (0·04 to 0·08)
New Zealand	0·1 (-0·03 to 0·1)	0·07 (0·04 to 0·11)	0·07 (0·05 to 0·1)	0·07 (0·04 to 0·09)
Niue	*	0·07 (0·03 to 0·1)	0·07 (0·05 to 0·09)	0·06 (0·04 to 0·08)
Palau	*	0·07 (0·03 to 0·1)	0·07 (0·05 to 0·09)	0·06 (0·04 to 0·08)
Papua New Guinea	0·04 (-0·01 to 0·04)	0·03 (0·01 to 0·04)	0·03 (0·02 to 0·04)	0·02 (0·02 to 0·03)
Philippines	0·09 (-0·03 to 0·09)	0·07 (0·03 to 0·1)	0·07 (0·05 to 0·09)	0·06 (0·04 to 0·08)
Republic of Korea	0·08 (-0·03 to 0·08)	0·06 (0·03 to 0·09)	0·06 (0·04 to 0·08)	0·06 (0·04 to 0·08)
Samoa	0·03 (-0·01 to 0·03)	0·02 (0·01 to 0·03)	0·02 (0·02 to 0·03)	0·02 (0·01 to 0·03)
Singapore	0·08 (-0·03 to 0·08)	0·06 (0·03 to 0·09)	0·06 (0·05 to 0·09)	0·06 (0·04 to 0·08)
Solomon Islands	0·04 (-0·01 to 0·04)	0·03 (0·02 to 0·05)	0·03 (0·02 to 0·05)	0·03 (0·02 to 0·04)
Tonga	*	0·03 (0·02 to 0·05)	0·03 (0·02 to 0·04)	0·03 (0·02 to 0·04)
Tuvalu	*	0·07 (0·03 to 0·1)	0·07 (0·05 to 0·09)	0·06 (0·04 to 0·08)
Vanuatu	0·02 (-0·01 to 0·02)	0·01 (0·01 to 0·02)	0·01 (0·01 to 0·02)	0·01 (0·01 to 0·02)
Viet Nam	0·06 (-0·02 to 0·06)	0·05 (0·02 to 0·07)	0·05 (0·03 to 0·06)	0·04 (0·03 to 0·06)

*No data available

Table S3. Estimated populational attributable fraction (PAFs) by health outcomes, WHO Regions, and World Bank income classification (% and 95% confidence interval), 2020

	Breast cancer	Colorectal cancer	Endometrial cancer	Gastric cancer	Oseophageal cancer	Renal cancer	Bladder cancer
**African Region (AFR)**	0·02 (0·01 to 0·04)	0·02 (0·01 to 0·04)	0·02 (-0·01 to 0·05)	0·05 (-0·01 to 0·11)	0·05 (-0·03 to 0·15)	0·05 (0·01 to 0·09)	0·01 (-0·01 to 0·04)
**Region of the Americas (AMR)**	0·04 (0·01 to 0·06)	0·04 (0·01 to 0·06)	0·04 (-0·02 to 0·09)	0·09 (-0·03 to 0·19)	0·09 (-0·06 to 0·25)	0·09 (0·02 to 0·16)	0·03 (-0·03 to 0·08)
**Eastern Mediterranean Region (EMR)**	0·04 (0·01 to 0·06)	0·04 (0·01 to 0·07)	0·04 (-0·02 to 0·09)	0·1 (-0·03 to 0·21)	0·1 (-0·06 to 0·27)	0·1 (0·02 to 0·18)	0·03 (-0·03 to 0·09)
**European Region (EUR)**	0·03 (0·01 to 0·05)	0·03 (0·01 to 0·05)	0·03 (-0·01 to 0·07)	0·07 (-0·02 to 0·17)	0·08 (-0·05 to 0·22)	0·08 (0·02 to 0·14)	0·02 (-0·02 to 0·07)
**South-East Asia Region (SEAR)**	0·03 (0·01 to 0·05)	0·02 (0·01 to 0·03)	0·03 (-0·02 to 0·08)	0·06 (-0·02 to 0·13)	0·06 (-0·03 to 0·18)	0·06 (0·01 to 0·11)	0·02 (-0·02 to 0·05)
**Western Pacific Region (WPR)**	0·02 (0·01 to 0·03)	0·03 (0·01 to 0·05)	0·02 (-0·01 to 0·04)	0·08 (-0·02 to 0·17)	0·08 (-0·05 to 0·23)	0·08 (0·02 to 0·14)	0·02 (-0·02 to 0·07)
**Low-income**	0·02 (0·01 to 0·03)	0·02 (0 to 0·03)	0·02 (-0·01 to 0·04)	0·04 (-0·01 to 0·1)	0·04 (-0·02 to 0·13)	0·04 (0·01 to 0·08)	0·01 (-0·01 to 0·04)
**Lower-middle-income**	0·03 (0·01 to 0·05)	0·03 (0·01 to 0·05)	0·03 (-0·02 to 0·08)	0·07 (-0·02 to 0·17)	0·08 (-0·05 to 0·22)	0·08 (0·02 to 0·14)	0·02 (-0·02 to 0·07)
**Upper-middle-income**	0·02 (0·01 to 0·03)	0·02 (0·01 to 0·04)	0·02 (-0·01 to 0·05)	0·06 (-0·02 to 0·13)	0·06 (-0·03 to 0·18)	0·06 (0·01 to 0·11)	0·02 (-0·02 to 0·05)
**High-income**	0·04 (0·01 to 0·06)	0·04 (0·01 to 0·06)	0·04 (-0·02 to 0·08)	0·09 (-0·03 to 0·2)	0·09 (-0·06 to 0·26)	0·09 (0·02 to 0·17)	0·03 (-0·03 to 0·08)

(continued from previous table)

	Coronary Heart Disease	Depression	Dementia	Hypertension	Stroke	Type 2 diabetes
**African Region (AFR)**	0·03 (0·02 to 0·05)	0·05 (0 to 0·1)	0·08 (0·04 to 0·13)	0·01 (0·01 to 0·02)	0·03 (0·02 to 0·05)	0·03 (0·02 to 0·04)
**Region of the Americas (AMR)**	0·06 (0·04 to 0·08)	0·09 (0 to 0·18)	0·14 (0·07 to 0·22)	0·02 (0·01 to 0·03)	0·06 (0·03 to 0·09)	0·06 (0·04 to 0·07)
**Eastern Mediterranean Region (EMR)**	0·07 (0·05 to 0·09)	0·1 (0 to 0·2)	0·12 (0·07 to 0·2)	0·02 (0·01 to 0·03)	0·07 (0·03 to 0·1)	0·06 (0·04 to 0·08)
**European Region (EUR)**	0·05 (0·04 to 0·07)	0·08 (0 to 0·15)	0·11 (0·06 to 0·17)	0·02 (0·01 to 0·03)	0·05 (0·03 to 0·08)	0·05 (0·03 to 0·06)
**South-East Asia Region (SEAR)**	0·04 (0·03 to 0·05)	0·06 (0 to 0·12)	0·11 (0·06 to 0·18)	0·01 (0·01 to 0·02)	0·04 (0·02 to 0·06)	0·04 (0·02 to 0·05)
**Western Pacific Region (WPR)**	0·05 (0·04 to 0·07)	0·08 (0 to 0·16)	0·07 (0·04 to 0·12)	0·02 (0·01 to 0·03)	0·05 (0·03 to 0·08)	0·05 (0·03 to 0·07)
**Low-income**	0·03 (0·02 to 0·04)	0·04 (0 to 0·09)	0·06 (0·03 to 0·1)	0·01 (0 to 0)	0·03 (0·01 to 0·04)	0·03 (0·02 to 0·04)
**Lower-middle-income**	0·05 (0·04 to 0·07)	0·08 (0 to 0·16)	0·11 (0·06 to 0·17)	0·02 (0·01 to 0·03)	0·05 (0·03 to 0·08)	0·05 (0·03 to 0·06)
**Upper-middle-income**	0·04 (0·03 to 0·06)	0·06 (0 to 0·12)	0·08 (0·04 to 0·14)	0·01 (0·01 to 0·02)	0·04 (0·02 to 0·06)	0·04 (0·02 to 0·05)
**High-income**	0·07 (0·05 to 0·09)	0·09 (0 to 0·19)	0·13 (0·07 to 0·2)	0·02 (0·01 to 0·03)	0·07 (0·03 to 0·09)	0·06 (0·04 to 0·08)

Table S4. Estimated direct healthcare cost per incident case of cancer by WHO Region and country in US$ 2020

African region (AFR)	Breast	Colorectal	Endometrial	Gastric	Oesophageal	Renal	Bladder
Algeria	1806	2317	2107	1204	1204	1987	2710
Angola	1248	1348	1373	832	832	1373	1872
Benin	598	897	658	399	399	658	897
Botswana	1737	2926	2660	1158	1158	1910	2605
Burkina Faso	791	1291	870	527	527	870	1186
Burundi	1058	1599	1629	987	987	1629	2221
Cabo Verde	1290	1277	1257	860	860	1419	1759
Cameroon	1118	1561	1953	746	746	1230	1525
Central African Republic	597	832	657	398	398	657	895
Chad	594	1362	654	396	396	654	891
Comoros	1628	2001	1790	1194	1194	1791	1791
Congo	1622	2818	1849	1189	1189	1784	1784
Côte d'Ivoire	1436	1685	1709	1053	1053	1580	1580
Democratic Republic of the Congo	1039	882	743	450	450	743	1013
Equatorial Guinea	2691	2374	1885	1866	1794	2960	4037
Eritrea	860	993	497	301	301	497	677
Eswatini	1350	1466	800	800	1350	1485	2025
Ethiopia	800	776	660	400	400	660	900
Gabon	1815	3769	1979	1210	1210	1997	2723
Gambia	806	1828	1862	1128	1128	1862	2539
Ghana	1835	2648	869	1668	1668	2018	2752
Guinea	448	985	1969	1193	1193	1969	2685
Guinea-Bissau	1189	1284	1307	792	792	1307	1783
Kenya	1383	1568	858	1383	1383	1522	2075
Lesotho	1761	1460	1368	1291	1291	1420	1937
Liberia	892	2158	981	594	594	981	1337
Madagascar	948	1896	1043	632	632	1043	1422
Malawi	899	1723	989	599	599	989	1349
Mali	996	784	575	349	349	575	784
Mauritania	1402	2325	2034	1402	1402	1542	2103
Mauritius	2638	1969	1790	1759	1759	2902	3957

Mozambique	1196	1128	877	532	532	877	1196
Namibia	1935	3567	1779	1290	1290	2128	2902
Niger	768	1742	1775	1076	1076	1775	2420
Nigeria	1453	1719	1467	1065	1065	1598	1598
Rwanda	792	1235	654	396	396	654	891
Sao Tome and Principe	1464	1719	1110	1110	927	1576	1342
Senegal	847	1514	1625	565	565	932	1271
Sierra Leone	809	2053	890	539	539	890	1213
Somalia	1251	870	894	542	542	894	1220
South Africa	1557	4052	3684	1038	1038	1713	2336
South Sudan	918	1114	1010	612	612	1010	1378
Tanzania United Republic of	1248	1348	1373	832	832	1373	1872
Togo	774	1168	568	344	344	568	774
Uganda	1110	1069	814	493	493	814	1110
Zambia	1434	1458	1640	956	956	1434	1434
Zimbabwe	1686	1508	919	1237	1237	1360	1855
**AFR (mean)**	**1245**	**1704**	**1329**	**881**	**887**	**1353**	**1758**
**Region of the America (AMR)**	**Breast**	**Colorectal**	**Endometrial**	**Gastric**	**Oesophageal**	**Renal**	**Bladder**
Argentina	2103	3469	3154	1402	1402	2313	3154
Bahamas	10793	12128	15160	12735	10107	22235	16777
Barbados	7526	7931	9913	8327	6609	14539	10971
Belize	1940	2850	2591	1294	1294	2134	2911
Bolivia	1675	1787	791	791	1228	1351	1842
Brazil	1517	3041	2765	1011	1011	1669	2276
Canada	25895	17573	21967	18452	14644	32218	24310
Chile	26708	19052	23815	20005	15877	34929	26356
Colombia	1247	3032	2756	831	831	1372	1871
Costa Rica	1158	3167	2436	772	772	1273	1737
Cuba	1501	3180	2891	1001	1001	1651	2251
Dominican Republic	1880	1691	1537	1253	1253	2068	2820
Ecuador	1424	3817	3470	949	949	1566	2136
El Salvador	1422	1636	2035	1422	1422	1564	2132
Guatemala	1598	6535	5941	1065	1065	1758	2397
Guyana	1881	2462	1939	1254	1254	2069	2822

Haiti	922	996	1015	615	615	1015	1383
Honduras	1415	1617	2476	1415	1415	1557	2123
Jamaica	1605	3148	2862	1070	1070	1766	2408
Mexico	1351	3392	3084	901	901	1486	2027
Nicaragua	1382	1484	1024	1014	1014	1521	1521
Panama	17867	18205	22756	19115	15171	33375	25183
Paraguay	1773	2189	1990	1182	1182	1950	2659
Peru	1488	3396	3088	992	992	1637	2232
Saint Lucia	2034	2260	2054	1356	1356	2237	3051
Suriname	1185	1907	1734	790	790	1304	1778
Trinidad and Tobago	16222	32693	27244	45770	18163	29969	36180
United States of America	11911	12398	15498	13018	10332	22730	17151
Uruguay	7941	8266	10332	8679	6888	15153	11434
Venezuela (Bolivarian Republic of)	1450	3474	3158	967	967	1595	2175
**AMR (mean)**	**5293·82**	**6292·56**	**6715·78**	**5648·24**	**4052·47**	**8066·81**	**7268·89**
**Eastern Mediterranean Region (EMR)**	**Breast**	**Colorectal**	**Endometrial**	**Gastric**	**Oesophageal**	**Renal**	**Bladder**
Afghanistan	889	2005	978	593	593	978	1334
Bahrain	54946	71409	89262	74980	59508	130917	98783
Djibouti	1633	2151	1412	1198	1198	1796	1796
Egypt	1366	1682	809	1366	1366	1502	2049
Iran Islamic Republic of	1203	4957	4506	802	802	1324	1805
Iraq	1802	4437	903	903	903	1982	2702
Jordan	1354	3931	3573	903	903	1490	2031
Kuwait	42898	58490	73113	61415	48742	107232	80912
Lebanon	1251	2371	2156	834	834	1376	1876
Libya	1076	6245	4804	718	718	1184	1615
Morocco	1381	1817	2040	1381	1381	1519	2072
Oman	36162	35726	44657	37512	29771	65497	49420
Pakistan	1649	1585	1789	1209	1209	1814	1814
Qatar	21762	20726	25907	21762	17272	37998	28671
Saudi Arabia	27070	26453	33067	27776	22045	48498	36594
Sudan	1681	1370	1698	1233	1233	1356	1849
Syrian Arab Republic	854	939	939	569	569	939	1281
Tunisia	1664	1458	3248	1513	1513	1830	2496

United Arab Emirates	21439	15956	19944	16753	13296	29252	22072
Yemen	653	705	718	435	435	718	979
**EMR (mean)**	**11137**	**13221**	**15776**	**12693**	**10214**	**21960**	**17107**
**European Region (EUR)**	**Breast**	**Colorectal**	**Endometrial**	**Gastric**	**Oesophageal**	**Renal**	**Bladder**
Albania	1558	2511	2283	1039	1039	1714	2337
Armenia	1680	2848	2590	1120	1120	1848	2520
Austria	43754	36059	45074	37862	30049	66108	49882
Azerbaijan	1630	2372	2157	1087	1087	1793	2445
Belarus	1409	2464	2240	939	939	1550	2114
Belgium	27653	24600	30750	25830	20500	45100	34030
Bosnia and Herzegovina	1612	3068	2789	1075	1075	1773	2418
Bulgaria	1190	1911	1738	794	794	1309	1785
Croatia	8822	6660	8325	6993	5550	12210	9213
Cyprus	17028	15339	19174	16106	12782	28121	21219
Czech Republic	14662	10198	12748	10708	8498	18697	14107
Denmark	32302	21986	27483	23086	18322	40308	30414
Estonia	12081	7576	9470	7955	6313	13890	10480
Finland	17745	15264	19080	16027	12720	27984	21115
France	32239	23844	29805	25036	19870	43714	32984
Georgia	2608	2192	854	854	854	2869	3912
Germany	35782	25354	31692	26622	21128	46482	35073
Greece	11917	8205	10256	8615	6837	15042	11350
Hungary	7427	6495	8119	6820	5413	11908	8985
Iceland	29685	26539	33174	27866	22116	48655	36713
Ireland	34268	22645	28306	23777	18871	41515	31325
Israel	39089	41401	51751	43471	34501	75902	57271
Italy	18259	16051	20063	16853	13375	29426	22203
Kazakhstan	1580	4102	3729	1054	1054	1738	2371
Kyrgyzstan	1373	1464	939	915	915	1373	1373
Latvia	8585	7144	8930	7501	5953	13097	9882
Lithuania	11089	8861	11076	9304	7384	16245	12257
Luxembourg	43400	40998	51248	43048	34165	75164	56715
Malta	19228	19725	24656	20711	16438	36163	27286
Moldova Republic of	1524	1674	1534	1758	1395	2250	1698

Montenegro	1165	2522	2293	777	777	1281	1747
Netherlands	32488	26346	32933	27664	21955	48302	36446
North Macedonia	15834	14150	17688	14858	11792	25942	19575
Norway	41865	27852	34815	29244	23210	51061	38528
Poland	10690	7932	9915	8329	6610	14542	10973
Portugal	14077	10121	12651	10627	8434	18555	14001
Romania	1292	2551	2319	862	862	1422	1938
Russian Federation	1321	2842	2584	881	881	1454	1982
Serbia	1304	2098	1907	869	869	1434	1956
Slovakia	14375	10166	12708	10675	8472	18638	14063
Slovenia	17058	11727	14659	12313	9773	21500	16223
Spain	15947	11617	14521	12197	9681	21297	16070
Sweden	23410	18505	23131	19430	15421	33926	25599
Switzerland	62458	46616	58271	48947	38847	85464	64486
Tajikistan	784	1984	863	523	523	863	1177
Turkey	1168	3543	3221	779	779	1285	1752
Turkmenistan	1764	2704	2459	1176	1176	1940	2646
Ukraine	1750	782	798	1284	1284	1412	1926
United Kingdom	95309	62873	78591	66016	52394	115267	86974
Uzbekistan	1314	1451	1713	876	876	1446	1792
**EUR (mean)**	**16731**	**13559**	**16601**	**13663**	**10913**	**23820**	**18307**
**South-East Africa Region (SEAR)**	**Breast**	**Colorectal**	**Endometrial**	**Gastric**	**Oesophageal**	**Renal**	**Bladder**
Bangladesh	1890	2748	1154	1718	1718	2079	2834
Bhutan	731	1410	1683	488	488	804	1097
Democratic People's Republic of Korea	903	1235	662	401	401	662	903
India	2077	3129	11293	1385	1385	2284	3115
Indonesia	1356	1567	685	1356	1356	1492	2035
Maldives	1541	7291	6628	1027	1027	1695	2311
Myanmar	1267	1380	1174	844	844	1393	1727
Nepal	876	1051	963	584	584	963	1313
Sri Lanka	1888	2844	10266	1259	1259	2077	2832
Thailand	1221	3092	2811	814	814	1344	1832
Timor-Leste	599	1060	1391	399	399	659	899
**SEAR (mean)**	**1304**	**2437**	**3519**	**934**	**934**	**1405**	**1900**

Western Pacific Region (WPR)	Breast	Colorectal	Endometrial	Gastric	Oesophageal	Renal	Bladder
Australia	34359	19335	24168	20301	16112	35447	26746
Brunei Darussalam	6893	8590	10737	9019	7158	15748	11882
Cambodia	2119	1055	857	1413	1413	2331	3179
China	1676	3447	3133	1117	1117	1843	2514
Fiji	1346	3743	3403	897	897	1480	2018
Japan	46636	20938	26172	21985	17448	38386	28964
Kiribati	1522	1725	944	1522	1522	1674	2282
Korea Republic of	10037	5103	6378	5358	4252	9355	7059
Lao People's Democratic Republic	676	1595	798	450	450	743	1013
Malaysia	1872	2151	1955	1248	1248	2059	2808
Mongolia	1601	1455	997	1174	1174	1761	1761
New Zealand	22796	13486	16857	14160	11238	24724	18656
Papua New Guinea	1593	1472	1669	1168	1168	1753	1753
Philippines	1708	1612	957	1253	1253	1378	1879
Samoa	1301	3090	2809	867	867	1431	1951
Singapore	14437	10382	12978	10901	8652	19034	14362
Solomon Islands	236	1191	1251	1251	158	260	354
Vanuatu	1774	2293	399	1196	1533	2102	2440
Viet Nam	1020	2159	783	680	680	1122	1391
**WPR (mean)**	**8084**	**5517**	**6171**	**5051**	**4123**	**8559**	**7001**

Table S5. Estimated direct healthcare costs for stroke, coronary heart disease and hypertension, by country and WHO Regions, in US$ 2020 ·

WHO Region	Stroke	Coronary Heat Disease	Hypertension
**African Region**	**283·96**	**86·00**	**55·64**
Algeria	916·32	300·52	178·70
Angola	175·33	54·13	30·57
Benin	77·91	24·80	15·45
Botswana	1030·39	280·34	202·09
Burkina Faso	121·84	29·07	20·74
Burundi	56·09	14·24	12·21
Cabo Verde	354·65	90·48	74·16
Cameroon	120·22	37·78	24·80
Central Africa Republic	51·69	10·97	18·01
Chad	80·92	29·02	14·67
Comoros (The)	123·84	37·79	24·91
Congo	159·66	60·86	23·27
Côte d'Ivoire	164·93	44·59	32·72
Democratic Republic of the Congo	159·66	10·14	5·70
Equatorial Guinea	697·43	252·54	129·30
Eritrea	56·37	21·15	13·68
Eswatini	668·91	202·00	129·13
Ethiopia	61·88	18·15	12·37
Gabon	466·47	138·62	91·11
Ghana	140·17	54·72	31·18
Guinea	95·49	33·78	20·28
Guinea*Bissau	112·18	39·60	22·87
Kenya	148·84	45·65	0·04
Lesotho	267·93	76·20	57·36
Liberia	116·69	39·08	19·09
Madagascar	82·03	24·66	14·68
Malawi	114·28	33·49	22·18
Mali	86·11	24·71	16·71
Mauritania	171·18	54·19	35·29
Mauritius	1207·03	337·26	256·29
Mozambique	104·37	26·63	21·86
Namibia	869·28	317·22	163·84
Niger	73·29	20·25	14·48
Nigeria	208·82	63·67	43·25
Rwanda	136·00	37·97	31·52
Sao Tome and Principe	201·84	48·21	39·71
Senegal	135·35	41·13	27·17
Seychelles	1361·22	350·69	287·52
Sierra Leone	241·11	85·30	47·76
South Africa	1029·48	316·52	209·63
South Sudan	139·64	86·00	21·12
the Gambia	77·77	22·09	15·00
Togo	95·84	28·67	20·30

Uganda	124·93	38·72	25·86
United Republic of Tanzania	103·03	28·95	20·87
Zambia	169·86	50·99	38·69
Zimbabwe	187·76	58·45	36·76
**Region of the Americas**	**1397·72**	**407·96**	**281·54**
Antigua and Barbuda	1203·64	346·52	260·94
Argentina	2063·44	616·21	369·31
Bahamas (The)	2009·94	468·04	372·21
Barbados	*	*	*
Belize	456·09	145·78	93·98
Bolivia (Plurinational State of)	458·48	131·97	92·08
Brazil	1400·11	410·60	284·15
Canada	4744·06	1384·43	965·21
Chile	2071·44	551·41	427·97
Colombia	1052·15	308·45	214·46
Costa Rica	1174·98	357·48	248·08
Cuba	2377·70	930·97	467·63
Dominica	551·22	164·81	131·83
Dominican Republic (The)	901·46	246·47	188·87
Ecuador	896·89	287·40	177·22
El Salvador	544·20	166·48	109·95
Grenada	635·59	180·66	128·78
Guatemala	448·41	133·53	89·65
Guyana	441·43	118·91	95·21
Haiti	138·83	44·61	26·66
Honduras	325·07	99·37	67·24
Jamaica	521·43	141·54	103·78
Mexico	1012·39	310·28	197·86
Nicaragua	446·71	120·63	87·97
Panama	1594·50	443·22	344·63
Paraguay	815·22	233·20	173·61
Peru	659·34	192·20	142·30
Saint Kitts and Nevis	1466·72	406·01	288·74
Saint Lucia	601·31	168·91	113·32
Saint Vicent and the Grenadines	479·24	133·72	102·02
Suriname	882·65	281·69	218·96
Trinidad and Tobago	2065·07	597·15	389·72
United States of America	9642·00	2778·27	1971·97
Uruguay	2043·18	561·69	402·66
Venezuela (Bolivarian Republic of)			
**Eastern Mediterranean Region**	**1455·59**	**394·30**	**264·55**
Afghanistan	215·50	55·98	34·60
Bahrain	2137·22	683·17	362·95
Egypt	624·66	170·88	113·99
Iran (Islamic Republic of)	1661·20	394·65	313·94
Iraq	682·49	147·04	132·89
Jordan	708·13	201·34	136·94
Kuwait	3551·16	881·89	681·03

Lebanon	1021·16	285·38	201·55
Libya	*	*	
Morocco	415·16	117·90	86·71
Oman	1559·73	528·07	321·03
Pakistan	143·67	37·46	33·08
Qatar	3202·82	1149·70	587·64
Saudi Arabia	3551·22	946·37	653·36
Somalia	*	*	
Sudan	139·64	85·14	54·40
Syrian Arab Republic	*	*	
Tunisia	825·73	237·80	169·33
United Arab Emirates	2862·72	736·53	588·89
Yemen			
Djibouti	126·96	43·86	25·04
**European Region**	**2457·25**	**692·39**	**544·01**
Albania	609·58	166·58	129·43
Andorra	3134·85	906·32	669·52
Armenia	941·55	258·48	192·48
Austria	6965·86	1800·85	1091·26
Azerbaijan	618·98	216·65	117·60
Belarus	1034·16	328·24	210·21
Belgium	4031·53	1060·68	1003·25
Bosnia and Herzegovina	1158·55	328·42	241·45
Bulgaria	1487·54	396·70	303·26
Croatia	1674·84	454·82	348·24
Cyprus	941·25	708·21	487·22
Czechia	2244·23	329·88	564·37
Denmark	2440·61	947·35	1075·52
Estonia	3423·09	288·82	450·61
Finland	7274·60	1421·74	827·33
France	3083·01	1027·69	974·58
Georgia	693·74	202·79	147·73
Germany	7298·74	1385·57	1131·93
Greece	1753·80	1014·04	434·38
Hungary	1488·15	439·61	392·62
Iceland	4476·22	1164·86	949·10
Ireland	4154·04	1181·07	1094·53
Israel	2916·28	774·28	595·36
Italy	4198·48	1018·49	672·69
Kazakhstan	761·88	223·23	145·48
Kyrgyzstan	218·41	72·24	48·25
Latvia	753·61	206·14	351·89
Lithuania	1123·26	297·73	429·33
Luxembourg	5319·76	1925·11	1122·58
Malta	2032·11	671·67	723·41
Monaco	*	*	
Montenegro	1616·07	*	
Netherlands	4674·19	1934·54	1045·87

North Macedonia	955·05	256·78	241·80
Norway	6153·64	1798·52	1265·61
Poland	1121·55	608·62	374·07
Portugal	1602·32	688·28	601·84
Republic of Moldova	454·57	152·33	89·07
Romania	1291·77	168·22	292·59
Russian Federation	1312·11	376·64	276·26
San Marino	*	*	
Serbia	1273·96	384·20	275·58
Slovakia	1945·41	592·49	404·56
Slovenia	1747·08	340·90	586·25
Spain	3177·01	1030·99	663·86
Sweden	4758·34	802·60	1081·86
Switzerland	7487·39	2139·49	1506·09
Tajikistan	219·22	57·65	46·36
Turkey	1110·64	310·44	217·32
Turkmenistan	1180·80	295·18	236·68
Ukraine	582·65	173·08	126·69
United Kingdom of Great Britain and Northern Ireland (the)	4010·34	1182·04	857·47
Uzbekistan	392·82	108·26	85·28
**South East Asian Region**	**407·56**	**115·81**	**85·18**
Bangladesh	91·88	26·67	20·35
Bhutan	297·10	91·25	59·79
Democratic People's Republic of Korea	*	*	
India	239·33	64·56	51·07
Indonesia	332·61	96·87	69·64
Maldives	1272·48	344·59	267·77
Myanmar	270·78	81·73	54·15
Nepal	148·98	47·73	33·49
Sri Lanka	470·29	135·11	95·95
Thailand	648·42	174·94	134·15
Timor*Leste	303·70	94·66	65·43
**Western Pacific Region**	**1224·32**	**353·67**	**246·93**
Australia	4462·73	1284·74	928·99
Brunei Darussalam	1710·72	560·25	362·42
Cambodia	224·87	63·71	48·48
China	791·19	205·47	173·59
Cook Islands			
Fiji	310·89	91·21	69·14
Japan	4142·58	1278·71	835·96
Kiribati	226·59	49·39	51·57
Lao People's Democratic Republic	165·56	43·64	31·02
Malaysia	1052·71	302·91	221·60
Marshall Islands	572·18	175·33	125·69
Micronesia (Federated States of)	402·88	122·13	76·85
Mongolia	483·83	150·12	96·39
Nauru	1351·20	438·46	218·00

New Zealand	3603·52	1019·13	747·00
Niue			
Palau	2044·07	598·79	373·37
Papua New Guinea	91·67	21·73	18·80
Philippines	350·20	92·51	73·12
Republic of Korea	2766·22	741·00	596·51
Samoa	341·61	98·80	65·93
Singapore	3960·74	1078·13	824·01
Solomon Islands	104·47	33·99	19·98
Tonga	328·81	80·48	65·96
Tuvalu	653·57	176·02	140·66
Vanuatu	81·45	35·96	20·17
Viet Nam	383·75	99·11	81·70
**World Bank income classification**			
Low	109·26	33·13	19·23
Lower-middle	316·44	91·62	64·03
Upper-middle	929·86	265·73	178·35
High	3239·25	916·90	671·22

*No information

Table S6. Total number of estimated cases by health outcomes attributed to physical inactivity by country, 2020-2030

	Cancers			Coronary Heart Disease			Depression		
**African Region**	**Cost amount**	**Lower limit**	**Upper limit**	**Cost amount**	**Lower limit**	**Upper limit**	**Cost amount**	**Lower limit**	**Upper limit**
Algeria	12792	2764	24503	132533	92435	177437	2061219	80269	4132620
Angola	1898	381	3909	10041	6943	13573	824234	30913	1721609
Benin	708	108	1533	3737	2582	5058	201589	7510	424413
Botswana	196	27	479	1510	1044	2035	68676	2598	142178
Burkina Faso	1178	207	2526	8395	5813	11334	443534	16709	921905
Burundi	763	106	1774	4150	2871	5604	237517	8907	496109
Cabo Verde	127	5	291	410	284	556	18588	697	38706
Cameroon	2710	654	5042	15168	10549	20371	909782	34987	1848620
Central Africa Republic	196	42	395	1616	1115	2189	127657	4734	270040
Chad	1032	237	1985	6280	4353	8460	395330	15007	814780
Comoros (The)	40	5	118	336	227	452	13166	487	27846
Congo	345	71	676	3810	2649	5119	244997	9410	498490
Côte d'Ivoire	2909	680	5393	17916	12494	23994	790714	30755	1587373
Democratic Republic of the Congo	5824	996	12232	41077	28494	55328	2995028	113822	6164201
Equatorial Guinea	86	16	155	434	301	583	41869	1570	87451
Eritrea	361	68	732	2491	1726	3359	182352	6905	376775
Eswatini	69	12	141	822	572	1106	38986	1500	79325
Ethiopia	7365	1549	14832	26280	18139	35601	1772837	65858	3743519
Gabon	217	50	415	1287	893	1733	79973	3050	163918
Ghana	2561	497	5228	17502	12126	23605	812581	30726	1681801
Gambia (The)	77	12	173	1143	791	1540	65363	2468	135553
Guinea	359	62	726	3848	2655	5211	185593	6886	392373
Guinea-Bissau	105	24	195	702	483	949	38008	1425	79394
Kenya	4519	607	10574	15501	10702	20990	970065	36087	2045340
Lesotho	60	6	136	401	273	548	24208	880	52500
Liberia	356	69	695	2767	1920	3728	139657	5323	286397
Madagascar	2076	340	4568	9312	6442	12587	575170	21555	1202363
Malawi	1583	135	4220	5581	3854	7558	262843	9782	553865

Mali	3778	579	7712	15606	10924	20812	586200	23209	1155430
Mauritania	673	122	1330	4305	3009	5732	134629	5345	264785
Mauritius	634	130	1245	1661	1151	2229	52472	2024	106246
Mozambique	547	57	1491	3134	2155	4277	189639	6875	412187
Namibia	448	100	850	2256	1574	3021	77204	3006	154871
Niger	1272	268	2526	7985	5536	10768	408938	15486	844955
Nigeria	16950	4231	31020	106316	73887	142909	4743396	181770	9674681
Rwanda	719	106	1565	3414	2357	4625	253933	9426	536683
Sao Tome and Principe	22	-	42	83	56	113	3088	114	6508
Senegal	1430	245	2947	8855	6141	11939	337038	12787	695025
Seychelles	*	*	*	68	47	93	1569	59	3270
Sierra Leone	407	87	825	2875	1984	3898	131065	4860	277252
South Africa	20631	4008	41458	88884	62142	118670	3134411	123449	6211665
South Sudan	803	124	1803	3308	2289	4469	190134	7132	397135
Togo	303	46	676	1825	1254	2481	92867	3407	199176
Uganda	916	93	2399	3639	2500	4964	395202	14322	859271
United Republic of Tanzania	1478	180	3740	7733	5313	10539	420074	15264	910399
Zambia	*	*	*	6603	4577	8904	387209	14654	800730
Zimbabwe	*	*	*	10156	7054	13653	307154	11763	626982
	**Cancers**			**Coronary Heart Disease**			**Depression**		
**Region of the Americas**	**Cost amount**	**Lower limit**	**Upper limit**	**Cost amount**	**Lower limit**	**Upper limit**	**Cost amount**	**Lower limit**	**Upper limit**
Antigua and Barbuda	*	*	*	265	187	358	3541	138	7074
Argentina	33325	7019	63244	71486	50070	95249	1609903	63926	3163961
Bahamas (The)	218	47	411	1317	923	1752	17813	711	34869
Barbados	284	65	519	1625	1140	2162	16261	648	31857
Belize	71	15	130	838	584	1120	14346	562	28663
Bolivia (Plurinational State of)	2614	380	5280	5238	3655	7008	446991	17462	893212
Brazil	145303	28360	261030	244096	171453	324236	14344734	577022	27832887
Canada	33413	6889	65514	76659	53320	102948	1088541	41870	2211261
Chile	9681	1466	19884	22639	15730	30437	704562	26965	1438982
Colombia	29256	4868	56850	80527	56471	107144	1590063	63506	3107029

Costa Rica	3392	554	6548	8412	5903	11175	252774	10144	491483
Cuba	8604	1463	17470	45748	31965	61129	716858	28143	1425261
Dominica	*	*	*	307	215	410	3345	132	6540
Dominican Republic (The)	3673	731	7024	27643	19333	36889	536910	21185	1061926
Ecuador	4832	686	9975	6614	4595	8887	525744	20151	1072027
El Salvador	2026	352	3961	7514	5244	10058	250583	9790	500734
Grenada	*	*	*	271	186	363	3553	135	7215
Guatemala	3335	430	6778	14192	9917	18961	703934	27649	1398864
Guyana	196	40	334	1876	1309	2511	46382	1810	92685
Haiti	2720	381	5536	19705	13749	26355	458040	17895	915293
Honduras	1747	299	3453	8555	5975	11447	304123	11883	607723
Jamaica	1253	247	2453	8133	5667	10892	99563	3865	200133
Mexico	30839	5856	60226	131867	91735	177057	4551485	175199	9238470
Nicaragua	1634	279	3207	5990	4180	8011	228266	8915	456136
Panama	1623	285	3160	5153	3596	6894	141709	5537	283175
Paraguay	2313	444	4494	4217	2942	5632	307097	12073	609809
Peru	14488	2065	29150	17131	11957	22919	843255	32946	1685053
Saint Kitts and Nevis	*	*	*	169	161	301	3034	163	7864
Saint Lucia	99	17	190	702	487	932	8847	351	17462
Saint Vicent and the Grenadines	*	*	*	380	266	506	4893	189	9781
Suriname	210	52	382	2272	1594	3022	42417	1694	82802
Trinidad and Tobago	804	164	1523	6331	4425	8455	78742	3103	156049
United States of America	382785	80743	735074	774818	542232	1033387	19043730	753296	37572999
Uruguay	2316	451	4639	4602	3191	6205	87117	3300	180002
Venezuela (Bolivarian Republic of)	9668	1895	18756	37820	26346	50701	1017788	39423	2052279
	**Cancers**			**Coronary Heart Disease**			**Depression**		
**Eastern Mediterranean Region**	**Cost amount**	**Lower limit**	**Upper limit**	**Cost amount**	**Lower limit**	**Upper limit**	**Cost amount**	**Lower limit**	**Upper limit**
Afghanistan	6727	791	14322	67342	13330	38684	1819018	71832	3595073
Bahrain	312	67	613	5461	490	1426	110551	4365	218487
Djibouti	272	44	562	1081	423	1219	56895	2247	112449
Egypt	21440	4091	43967	304740	47802	140655	3750040	145105	7569556

Iran (Islamic Republic of)	34907	4449	73584	428073	34663	101623	5629731	219023	11298892
Iraq	10653	2390	19746	153764	30631	87089	2565479	104419	4921216
Jordan	923	186	1843	10194	1862	5665	190520	7023	405968
Kuwait	1476	346	2683	23131	2637	7314	457898	19290	850730
Lebanon	2015	425	3920	25205	2880	8403	324472	12723	645933
Libya	1591	349	3051	22973	3458	10082	416061	16317	828247
Morocco	9044	1884	17685	110011	17172	50949	1815326	69405	3711614
Oman	810	142	1583	8833	1212	3546	226401	8803	454730
Pakistan	37965	5652	82196	391832	65226	191052	7950284	309688	15935724
Qatar	335	71	636	5120	700	2035	184520	7242	366956
Saudi Arabia	9230	2004	17068	121082	21860	62036	3004372	122605	5748961
Somalia	2374	433	4806	11794	5345	15511	770809	30439	1523414
Sudan	6268	1335	12236	95486	18553	53830	1952767	77114	3859413
Syrian Arab Republic	4860	1055	9237	67868	8668	25157	756346	29870	1494828
Tunisia	3188	653	6320	45326	6184	18205	704942	27239	1425193
United Arab Emirates	1302	286	2434	24499	6201	17933	615954	24449	1211126
Yemen	*	*	*	66746	12033	34919	1636718	64630	3234781
	**Cancers**			**Coronary Heart Disease**			**Depression**		
**European Region**	**Cost amount**	**Lower limit**	**Upper limit**	**Cost amount**	**Lower limit**	**Upper limit**	**Cost amount**	**Lower limit**	**Upper limit**
Albania	1777	182	3571	6325	4402	8493	67811	2611	137457
Andorra	*	*	*	1305	800	800	30594	13197	13197
Armenia	1416	209	2599	10910	7566	14706	76029	2880	157003
Austria	7871	1415	13824	23373	16268	31361	321305	12405	650097
Azerbaijan	4236	410	8491	34147	23760	45835	259437	10000	525895
Belarus	4686	683	9123	26253	18115	35587	232764	8632	492688
Belgium	18746	2782	31184	31624	22078	42286	551766	21598	1100343
Bosnia and Herzegovina	2462	374	4435	8954	6219	12047	92834	3544	190171
Bulgaria	8809	1392	14899	37261	26055	49733	293385	11565	580848
Croatia	5685	932	9724	13769	9587	18460	169272	6552	341590
Cyprus	1217	236	1507	2229	1117	2143	64503	1808	94300
Czechia	13860	2488	24163	47090	32798	63141	379295	14680	765416

Denmark	7527	1320	13740	13336	10088	19419	223205	9362	488227
Estonia	1796	289	3172	10469	7099	13665	70415	2655	138425
Finland	3324	618	11054	10384	13197	25401	139703	9751	508451
France	76845	14400	139885	137180	101086	194611	2871992	117440	6123192
Georgia	1531	226	4665	11670	13713	26399	90299	5833	304224
Germany	151813	23529	196867	389863	204094	392919	4816131	141301	7367307
Greece	17334	2553	23233	34527	20072	38640	842559	27360	1426465
Hungary	16686	2926	23371	48720	27773	53471	429588	13688	713627
Iceland	333	60	598	711	523	1007	10060	408	21390
Ireland	5794	1003	9935	9702	6448	12408	243964	9018	470154
Israel	5332	938	9426	11403	8375	16123	362601	14781	770644
Italy	118178	18863	149991	267057	142305	273966	3670804	109562	5712327
Kazakhstan	7668	907	17765	46775	36602	70466	547874	23758	1238746
Kyrgyzstan	834	74	3742	7175	10838	20866	88035	7288	379877
Latvia	2644	418	4959	10797	7905	15222	97578	3964	206731
Lithuania	3268	496	7074	14189	11500	22146	137704	6178	322256
Luxembourg	514	95	1003	835	635	1223	21279	895	46701
Malta	717	129	933	1123	592	1143	21165	628	32724
Monaco	*	*	*	96	71	136	1868	76	3964
Montenegro	536	85	971	1719	1267	2432	19283	786	40986
Netherlands	26010	4291	44515	54229	37924	73008	724880	28081	1464134
North Macedonia	1565	244	2721	5437	3791	7305	62796	2431	126769
Norway	6631	1183	11051	11063	7567	14566	198573	7552	393742
Poland	45010	6958	76245	57104	38150	73450	991565	36859	1921680
Portugal	18222	3043	23714	16797	8567	16497	890912	25494	1329142
Republic of Moldova	1087	168	5309	6869	12486	24034	60519	6016	313602
Romania	23495	3777	36253	79058	48749	93849	684928	23544	1227741
Russian Federation	79383	11910	269759	396143	489190	941782	3092895	209864	10942069
San Marino	*	*	*	66	49	94	1572	66	3335
Serbia	12178	2104	16527	48174	26813	51621	372554	11597	604743
Slovakia	7682	1288	12423	18933	11830	22776	196289	6835	356434

Slovenia	2890	494	4881	7206	4858	9349	88761	3327	173491
Spain	53015	8771	100622	92252	73986	142432	2421690	107564	5608257
Sweden	7964	1397	17933	18518	17114	32943	375286	19156	998590
Switzerland	7323	1192	16121	14675	13235	25482	309019	15396	802720
Tajikistan	1884	157	4188	17458	12864	24767	186698	7634	398044
Turkey	46634	6413	80959	177014	125190	241010	3532475	138777	7235666
Turkmenistan	1670	187	3750	13390	9836	18936	126978	5177	269864
Ukraine	23736	3531	70024	205440	222354	428074	1564613	93239	4861530
United Kingdom of Great Britain and Northern Ireland (the)	92218	17051	147467	159347	22686	186688	3756262	127502	6647806
Uzbekistan	4947	618	15505	57603	13355	123055	561029	34263	1786482
	**Cancers**			**Coronary Heart Disease**			**Depression**		
**South-East Asia Region**	**Cost amount**	**Lower limit**	**Upper limit**	**Cost amount**	**Lower limit**	**Upper limit**	**Cost amount**	**Lower limit**	**Upper limit**
Bangladesh	33628	2587	83685	280906	195294	377425	7009681	269085	14270033
Bhutan	68	*	169	1049	1049	1049	16414	16414	16414
Democratic People's Republic of Korea	11012	1479	23426	25100	17397	33845	473582	17921	979346
India	257723	38105	550835	3345041	2333427	4477331	55440933	2161185	111041475
Indonesia	32307	7946	60285	85182	59051	114822	3651996	138371	7541557
Maldives	58	14	97	315	219	419	12812	492	25912
Myanmar	5708	544	13700	15856	10917	21538	298906	10984	639281
Nepal	1344	165	2958	22737	15678	30830	663433	24551	1407236
Sri Lanka	5343	794	11800	23404	16283	31423	500780	19276	1016468
Thailand	22251	4244	45112	64580	44822	86942	1634390	62238	3356371
Timor-Leste	78	10	141	450	314	608	13953	524	29212
	**Cancers**			**Coronary Heart Disease**			**Depression**		
**Western Pacific Region**	**Cost amount**	**Lower limit**	**Upper limit**	**Cost amount**	**Lower limit**	**Upper limit**	**Cost amount**	**Lower limit**	**Upper limit**
Australia	23946	4873	42126	90413	62950	121285	1200477	46380	2427013
Brunei Darussalam	182	34	335	258	178	345	6111	235	12459
Cambodia	855	144	1729	3843	2645	5222	136673	5019	292488
China	568870	51403	1303397	1050261	724568	1423517	19493977	722682	41262249
Cook Islands	*	*	*	18	13	25	321	10	665
Fiji	76	4	151	549	377	739	12336	461	25857

Japan	332430	35648	650558	317178	221435	424159	3893837	152346	7769115
Kiribati	*	*	*	5490	3839	7315	183568	7269	361823
Lao People's Democratic Republic	947	127	1987	16720	11548	22630	541863	20202	1139448
Malaysia	11372	2360	20117	38211	26725	51003	1216539	47984	2407307
Marshall Islands	*	*	*	56	39	72	1655	66	3244
Micronesia (Federated States of)	223	38	414	92	65	125	2588	103	5154
Mongolia	864	36	2094	4962	3431	6708	79841	2995	166762
Nauru	*	*	*	9	3	14	286	12	568
New Zealand	5737	1204	10137	15991	11206	21296	202100	8042	396420
Niue	*	*	*	3	-	5	57	-	115
Palau	*	*	*	31	21	42	676	25	1332
Papua New Guinea	841	131	1721	2977	2057	4033	107803	4006	227707
Philippines	33109	7483	58592	145034	101484	193469	3001831	118655	5926927
Republic of Korea	58592	5744	116227	66077	46130	88370	1567186	61300	3127698
Samoa	30	5	61	77	55	105	1715	64	3645
Singapore	5552	1112	9996	9598	6707	12829	162036	6354	322489
Solomon Islands	41	10	88	234	164	317	7961	299	16651
Tonga	*	*	*	59	40	80	1065	39	2231
Tuvalu	*	*	*	17	9	21	371	17	734
Vanuatu	17	*	16	65	43	88	1657	59	3577
Viet Nam	32018	4074	65813	71264	49479	95895	1518825	57953	3112188

*No data

(continued from previous page)

	**Hypertension**			**Stroke**			**Type 2 Diabetes**		
**African Region**	**Cost amount**	**Lower limit**	**Upper limit**	**Cost amount**	**Lower limit**	**Upper limit**	**Cost amount**	**Lower limit**	**Upper limit**
Algeria	1405861	709946	2088161	43295	21176	62041	104891	69189	139255
Angola	392690	197435	585803	8093	3908	11736	15908	10403	21294
Benin	132830	66726	198304	2979	1434	4330	4871	3182	6536
Botswana	45061	22674	67160	1172	565	1697	2628	1721	3515
Burkina Faso	314055	157977	468263	5332	2577	7722	10714	7017	14329

Burundi	143799	72298	214515	2937	1414	4259	4173	2733	5586
Cabo Verde	12245	6160	18265	249	121	361	535	350	717
Cameroon	451808	227821	672064	13097	6378	18842	20532	13506	27334
Central Africa Republic	55546	27894	82967	1494	717	2176	2666	1739	3574
Chad	263835	132833	393046	5069	2455	7325	7562	4958	10093
Comoros (The)	9636	4838	14394	260	123	377	266	176	356
Congo	106342	53612	158206	3022	1472	4351	5924	3899	7897
Côte d'Ivoire	606380	306174	900800	14772	7222	21180	22940	15128	30462
Democratic Republic of the Congo	1364238	686953	2032052	28344	13738	40928	61834	40568	82526
Equatorial Guinea	22016	11071	32847	307	150	445	798	524	1073
Eritrea	60092	30243	89544	2197	1063	3174	3465	2268	4628
Eswatini	26641	13431	39632	632	312	912	1481	978	1975
Ethiopia	1315670	660761	1964800	14499	6970	21092	24462	15968	32808
Gabon	44931	22634	66893	923	449	1328	2194	1440	2921
Ghana	34854	17543	51944	16238	7856	23482	23513	15406	31418
Gambia (The)	305459	153391	456221	3150	1514	4582	4179	2728	5607
Guinea	179135	90065	267224	710	340	1029	890	584	1193
Guinea-Bissau	23922	12013	35722	11591	5573	16854	15381	10044	20623
Kenya	228895	114662	342696	348	165	510	607	397	819
Lesotho	48834	24601	72712	1834	890	2647	3819	2508	5092
Liberia	68685	34527	102466	12367	5963	17942	9689	6338	12968
Madagascar	311021	156236	464383	3798	1825	5515	6652	4344	8918
Malawi	509186	257643	754850	11494	5656	16383	17747	11748	23480
Mali	588877	298041	872761	2861	1407	4076	3246	2152	4293
Mauritania	119849	60457	178211	1461	715	2099	6103	4020	8119
Mauritius	10701	5357	16025	3519	1677	5161	3628	2354	4891
Mozambique	713995	360539	1060575	1564	765	2243	2928	1933	3892
Namibia	48010	24164	71539	6091	2952	8809	7109	4661	9495
Niger	462272	232999	687906	73671	35823	106113	97720	64226	130185
Nigeria	1747324	877472	2609652	2730	1312	3975	3965	2589	5317
Rwanda	133970	67293	200032	91	45	137	97	62	133

Sao Tome and Principe	3309	1667	4932	5886	2852	8504	12626	8280	16862
Senegal	230628	115960	344023	83	42	117	232	150	310
Seychelles	1704	855	2542	2339	1125	3399	1997	1308	2682
Sierra Leone	237501	120095	352301	55102	27067	78688	125651	83095	166418
South Africa	1185471	596023	1768450	2707	1303	3926	3454	2259	4626
South Sudan	*	*	*	848	410	1225	1086	711	1453
Togo	59499	29837	88989	1552	744	2271	1584	1031	2132
Uganda	153170	76709	229376	3007	1433	4416	5607	3638	7559
United Republic of Tanzania	252149	126322	377491	5614	2676	8227	6982	4534	9411
Zambia	234238	117891	349074	4778	2312	6910	8559	5611	11435
Zimbabwe	277779	139996	413396	6332	3079	9124	15043	9885	20039
	**Hypertension**			**Stroke**			**Type 2 Diabetes**		
**Region of the Americas**	**Cost amount**	**Lower limit**	**Upper limit**	**Cost amount**	**Lower limit**	**Upper limit**	**Cost amount**	**Lower limit**	**Upper limit**
Antigua and Barbuda	3859	1951	5731	73	35	104	295	197	391
Argentina	2032980	1029020	3012786	38122	18783	54293	97286	64453	128626
Bahamas (The)	16283	8245	24119	360	180	514	1280	848	1691
Barbados	17783	9005	26343	399	198	570	1271	841	1684
Belize	9528	4815	14150	194	97	278	727	481	966
Bolivia (Plurinational State of)	237056	119756	351972	6055	2964	8664	16939	11181	22472
Brazil	10758934	5454263	15920090	265597	131482	376777	549724	365243	724819
Canada	964784	486498	1435074	24803	12078	35682	52295	34401	69612
Chile	550944	277649	819970	10868	5278	15655	35247	23161	46965
Colombia	1785523	904393	2644277	36573	18055	51998	132456	87866	174912
Costa Rica	198500	100604	293802	3629	1792	5150	14854	9865	19595
Cuba	513958	259791	762677	13849	6794	19793	35683	23582	47293
Dominica	*	*	*	88	43	124	314	206	417
Dominican Republic (The)	354265	179181	525389	10543	5182	15047	15953	10554	21118
Ecuador	324563	163595	482966	8133	3958	11717	24725	16251	32935
El Salvador	162776	82227	241684	3397	1663	4867	13591	8969	18025
Grenada	3395	1713	5051	84	40	121	301	198	401
Guatemala	391989	198154	581651	7437	3646	10625	39440	26068	52267

Guyana	22514	11375	33428	6779	3322	9707	2528	1669	3354
Haiti	300927	152020	446805	10367	5079	14842	25980	17148	34465
Honduras	219931	111104	326545	3766	1841	5389	18324	12098	24313
Jamaica	97032	48987	144166	2625	1284	3764	7875	5190	10465
Mexico	2944259	1484786	4379075	59132	28799	85048	323321	212728	430327
Nicaragua	158018	79827	234623	2741	1339	3925	13340	8806	17697
Panama	120374	60815	178729	2834	1385	4058	10022	6616	13295
Paraguay	230066	116311	341353	5704	2801	8154	12057	7970	15976
Peru	600062	303141	890964	16781	8216	24023	40702	26870	54003
Saint Kitts and Nevis	*	*	*	58	40	114	187	166	334
Saint Lucia	9910	5014	14691	216	105	303	846	562	1120
Saint Vicent and the Grenadines	4183	2115	6211	120	60	172	425	277	563
Suriname	24049	12181	35615	821	403	1168	2457	1631	3247
Trinidad and Tobago	73320	37075	108760	1579	773	2251	6694	4428	8864
United States of America	10851048	5489857	16088046	360457	177337	513999	1130464	748292	1495835
Uruguay	94098	47361	140212	2516	1221	3640	3334	2187	4459
Venezuela (Bolivarian Republic of)	733784	370316	1090596	18874	9214	27093	62847	41407	83534
	**Hypertension**			**Stroke**			**Type 2 Diabetes**		
**Eastern Mediterranean Region**	**Cost amount**	**Lower limit**	**Upper limit**	**Cost amount**	**Lower limit**	**Upper limit**	**Cost amount**	**Lower limit**	**Upper limit**
Afghanistan	1060300	536326	1572339	27114	13330	38684	70155	46424	92865
Bahrain	47657	24109	70671	997	490	1426	10902	7216	14430
Djibouti	2841236	1433708	4223300	97960	47802	140655	149961	98779	199368
Egypt	2295739	1159188	3410307	70894	34663	101623	187533	123673	249025
Iran (Islamic Republic of)	1375712	698423	2032813	61609	30631	87089	138917	92541	182706
Iraq	75886	38076	113427	3884	1862	5665	9046	5892	12148
Jordan	240622	122679	354088	5232	2637	7314	29685	19928	38768
Kuwait	224771	113601	333589	5875	2880	8403	14172	9364	18790
Lebanon	226038	114238	335472	7053	3458	10082	23078	15245	30596
Libya	1044258	526202	1554358	35349	17172	50949	65948	43324	87902
Morocco	122305	61750	181694	2472	1212	3546	9511	6272	12636
Oman	7045290	3557903	10464239	133339	65226	191052	308411	203451	409436

Pakistan	87982	44471	130560	1422	700	2035	17081	11290	22644
Qatar	1590473	807705	2349433	43925	21860	62036	167683	111763	220420
Saudi Arabia	455370	230337	675279	10875	5345	15511	14376	9515	19031
Somalia	1146158	579755	1699657	37733	18553	53830	74202	49100	98222
Sudan	699882	354019	1037866	17632	8668	25157	38342	25370	50753
Syrian Arab Republic	227763	114910	338610	12675	6184	18205	32548	21429	43282
Tunisia	515706	261020	764297	12591	6201	17933	50913	33727	67319
United Arab Emirates	*	*	*	24474	12033	34919	35737	23648	47303
Yemen	35481	17947	52617	854	423	1219	1224	807	1618
	**Hypertension**			**Stroke**			**Type 2 Diabetes**		
**European Region**	**Cost amount**	**Lower limit**	**Upper limit**	**Cost amount**	**Lower limit**	**Upper limit**	**Cost amount**	**Lower limit**	**Upper limit**
Albania	138738	69974	206317	3846	1877	5533	3149	2072	4190
Andorra	*	*	*	488	300	300	1236	846	846
Armenia	93809	47225	139786	2132	1034	3086	4188	2746	5594
Austria	400331	201957	595216	8132	3965	11683	15806	10405	21025
Azerbaijan	320105	161450	476033	10683	5204	15364	16481	10845	21929
Belarus	233528	117258	348828	8398	4036	12226	4299	2810	5773
Belgium	507907	256646	753955	10920	5353	15624	22930	15143	30408
Bosnia and Herzegovina	175401	88366	261137	6036	2930	8706	8952	5877	11937
Bulgaria	564366	285415	837073	25245	12404	36041	18034	11924	23881
Croatia	298931	150846	444328	6976	3405	10021	10691	7043	14211
Cyprus	60532	30665	89634	1069	376	1099	4767	2249	4534
Czechia	625262	315522	929386	14038	6851	20155	3410	2249	4534
Denmark	235019	118505	349592	4496	2383	7007	8289	5931	11968
Estonia	79153	39949	117621	1508	717	2106	1821	1166	2358
Finland	131899	66278	196879	3463	3084	9077	6860	8266	16684
France	2837885	1431306	4220371	47209	24374	71712	70615	49227	99348
Georgia	120303	60477	179498	4003	3296	9698	4671	5206	10500
Germany	5366928	2717012	7952202	114153	41872	123191	327886	162029	326989
Greece	578634	292555	858457	16649	6780	19948	22840	12544	25315
Hungary	766037	387390	1136228	20320	8117	23881	29241	15746	31775

Iceland	11536	5818	17153	212	110	324	518	364	732
Ireland	174128	87911	258704	2751	1280	3766	8424	5295	10675
Israel	199743	100746	297045	4490	2313	6800	13816	9598	19371
Italy	4171721	2111448	6182651	77737	29026	85396	202874	102058	205964
Kazakhstan	603201	304070	897516	19033	10437	30706	28831	21355	43093
Kyrgyzstan	81228	40783	121340	2221	2350	6908	2359	3381	6825
Latvia	119409	60228	177571	4223	2166	6370	2531	1755	3538
Lithuania	148924	75051	221654	5105	2900	8535	2601	1994	4030
Luxembourg	22824	11507	33947	344	184	538	1535	1102	2224
Malta	24927	12615	36940	506	186	550	1550	776	1562
Monaco	*	*	*	45	23	66	62	51	93
Montenegro	32834	16560	48823	1285	659	1944	1543	1070	2163
Netherlands	702056	353871	1044699	14524	7116	20935	25892	17137	34581
North Macedonia	*	*	*	4770	2332	6867	6682	4411	8902
Norway	216852	109446	322273	5066	2427	7140	10368	6707	13535
Poland	2341203	1181904	3478537	49013	22947	67506	88617	55986	112985
Portugal	790786	400472	1171313	15733	5624	16542	41907	20174	40711
Republic of Moldova	85772	43034	128216	2280	2906	8546	2109	3630	7334
Romania	1366140	690250	2028121	48005	20739	61021	34146	19895	40153
Russian Federation	4304836	2163405	6424633	159063	137636	404916	90247	105674	213262
San Marino	*	*	*	25	15	43	57	41	80
Serbia	661834	334792	981385	24612	9600	28240	29344	15426	31131
Slovakia	344259	173912	511145	8675	3799	11177	11264	6653	13426
Slovenia	141984	71676	210977	2376	1123	3298	4208	2681	5412
Spain	1753772	883878	2610008	33493	18827	55374	96808	73487	148308
Sweden	315961	159066	470726	7391	4786	14080	13297	11639	23491
Switzerland	257316	129566	383283	4772	3015	8869	12664	10812	21830
Tajikistan	201283	101518	299340	4361	2252	6625	11542	8045	16241
Turkey	2235399	1127865	3323143	76527	37921	111566	144478	96650	195042
Turkmenistan	154341	77843	229521	5627	2895	8519	5815	4041	8153
Ukraine	1589142	799215	2369937	51711	39217	115377	30726	31519	63610

United Kingdom of Great Britain and Northern Ireland (the)	2552633	1289917	3789003	53199	22686	66738	197657	113670	229394
Uzbekistan	575691	289486	858666	17182	13355	39302	30234	31804	64180
	**Hypertension**			**Stroke**			**Type 2 Diabetes**		
**South-East Asia Region**	**Cost amount**	**Lower limit**	**Upper limit**	**Cost amount**	**Lower limit**	**Upper limit**	**Cost amount**	**Lower limit**	**Upper limit**
Bangladesh	4032467	2032912	5999489	102022	49636	146859	170101	111849	226534
Bhutan	57877	28032	83633	266	130	379	822	822	822
Democratic People's Republic of Korea	548306	275958	817116	41409	20042	59883	25975	17024	34702
India	47173010	23824659	70059195	849183	415517	1216462	2588469	1707827	3435785
Indonesia	5944645	2992339	8857717	299224	144876	432526	266970	175032	356538
Maldives	14130	7128	21006	249	122	354	646	428	859
Myanmar	584679	293275	874229	16872	8077	24631	36859	24001	49556
Nepal	386425	193986	577329	5027	2413	7326	16734	10912	22463
Sri Lanka	697110	351550	1036831	19063	9284	27416	73419	48308	97717
Thailand	2263961	1140271	3371417	62884	30502	90750	116695	76596	155680
Timor-Leste	16670	8381	24874	767	369	1107	873	573	1173
	**Hypertension**			**Stroke**			**Type 2 Diabetes**		
**Western Pacific Region**	**Cost amount**	**Lower limit**	**Upper limit**	**Cost amount**	**Lower limit**	**Upper limit**	**Cost amount**	**Lower limit**	**Upper limit**
Australia	722289	364406	1073811	15674	7647	22520	36808	24235	48952
Brunei Darussalam	9698	4887	14427	287	141	415	1537	1009	2046
Cambodia	157820	79160	235995	5397	2581	7880	8612	5611	11580
China	21897988	10995113	32709777	1169515	561697	1702459	989399	645533	1327631
Cook Islands	486	244	727	16	7	22	42	28	56
Fiji	13657	6863	20381	476	228	692	2371	1549	3174
Japan	6908075	3490436	10255173	293046	143586	419303	200057	132103	265338
Kiribati	212677	107607	315285	8782	4321	12519	14043	9298	18576
Lao People's Democratic Republic	482059	242204	719606	15947	7676	23175	31630	20665	42390
Malaysia	1132344	572686	1679408	36449	17914	52028	72591	48019	96114
Marshall Islands	3524	1783	5218	91	44	128	330	220	440
Micronesia (Federated States of)	3333	1684	4950	128	66	184	359	238	476
Mongolia	62724	31535	93570	2357	1137	3422	1265	826	1694

Nauru	567	288	842	13	4	13	30	22	39
New Zealand	185565	93946	274935	3567	1761	5076	7655	5075	10116
Niue	121	62	182	0	0	0	10	5	13
Palau	1038	525	1537	36	18	49	99	65	129
Papua New Guinea	94662	47540	141372	3751	1805	5457	10225	6674	13715
Philippines	3202794	1620244	4748945	120955	59491	172516	166870	110440	220840
Republic of Korea	1325797	669862	1968227	66831	32744	95637	126369	83440	167608
Samoa	2029	1020	3035	84	43	123	210	142	285
Singapore	192449	97266	285613	5678	2787	8122	13527	8937	17931
Solomon Islands	6812	3427	10164	359	174	518	839	550	1126
Tonga	1472	739	2195	43	21	62	138	94	188
Tuvalu	718	361	1061	15	8	22	44	26	55
Vanuatu	1645	823	2458	87	43	126	162	108	225
Viet Nam	2583476	1301507	3846350	106438	51671	153505	132089	86736	176136

*No data

Table S7. Total number of estimated cases by health outcomes attributed to physical inactivity by WHO Regions and World Bank income classification, 2020-2030

	**Cancers**			**Coronary Heart Disease**			**Dementia**			**Depression**		
	Cases	Lower	Upper	Cases	Lower	Upper	Cases	Lower	Upper	Cases	Lower	Upper
African Region (AFR)	91072	17592	189209	488082	337587	659606	489194	254951	806932	21584993	809533	45085294
Region of the Americas (AMR)	687267	138820	1333233	1526438	1065327	2042028	3325931	1784152	5282201	39589118	1546665	79110075
Eastern Mediterranean Region (EMR)	174691	28862	357418	2261399	1581982	3017304	704325	375140	1128860	34848297	1376126	68873505
European Region (EUR)	804329	148080	1611968	2933301	2041096	3937251	3619975	1910500	5871576	33951645	1308532	68822395
South-East Asia Region (SEAR)	315630	53708	673175	2733001	1894049	3685185	2392199	1264868	3870699	45555456	1723848	94206623
Western Pacific Region (WPR)	1931309	265640	4079625	2957353	2059069	3966848	3338951	1729355	5554675	46607660	1801221	94202495
**Low-income**	**45190**	**7176**	**98495**	**280536**	**193774**	**379714**	**291450**	**150305**	**487772**	**12749620**	**475230**	**26818244**
**Lower-middle-income**	**551525**	**84713**	**1172948**	**5555980**	**3867314**	**7454804**	**3512999**	**1855930**	**5690470**	**93783768**	**3619913**	**189804252**
**Upper-middle-income**	**1284246**	**160804**	**2843168**	**3537171**	**2451776**	**4768621**	**4100154**	**2137983**	**6758453**	**57590456**	**2180920**	**118994008**
**High-income**	**1531653**	**276946**	**3037711**	**3135028**	**2190216**	**4189175**	**7255133**	**3876255**	**11581896**	**51612088**	**2025767**	**102641122**
**Total Global**	**3412614**	**529639**	**7152322**	**12508715**	**8703080**	**16792314**	**15159736**	**8020473**	**24518591**	**215735932**	**8301830**	**438257626**

(continued from previous Table)

	**Hypertension**			**Stroke**			**Type 2 Diabetes**		
	**Cases**	**Lower**	**Upper**	**Cases**	**Lower**	**Upper**	**Cases**	**Lower**	**Upper**
African Region (AFR)	16713759	8403254	24933050	355018	171245	514860	547218	357954	732431
Region of the Americas (AMR)	33208413	16776253	49306968	827763	405366	1184954	2707778	1787419	3592447
Eastern Mediterranean Region (EMR)	26570506	13440062	39401911	687125	337838	980348	1576853	1043379	2087251
European Region (EUR)	40629351	20492280	60420352	1085505	528914	1560584	1665577	1096141	2216192
South-East Asia Region (SEAR)	48944577	24633470	72939604	1107618	536029	1601702	2432208	1594155	3249112
Western Pacific Region (WPR)	65053617	32821732	96711421	3121103	1522306	4483201	2901910	1910953	3858976
**Low-income**	**10966964**	**5510003**	**16371643**	**201375**	**96912**	**292625**	**310727**	**202980**	**416456**
**Lower-middle-income**	**94488796**	**47665807**	**140491128**	**2282407**	**1112722**	**3279770**	**4583067**	**3017189**	**6096215**

**Upper-middle-income**	**66354447**	**33398687**	**98876032**	**2703037**	**1308498**	**3907852**	**3198315**	**2096644**	**4271824**
**High-income**	**62782805**	**31734178**	**93167857**	**1413101**	**693206**	**2019937**	**3155947**	**2085419**	**4182896**
**Total Global**	**234593012**	**##########**	**348906660**	**6599920**	**3211338**	**9500184**	**11248056**	**7402232**	**14967391**

Table S8. Direct healthcare costs attributable to physical inactivity, by health outcomes and by country (in US$), 2020-2030

	Cancers			Coronary Heart Disease			Depression		
**African Region**	**Cost amount**	**Lower limit**	**Upper limit**	**Cost amount**	**Lower limit**	**Upper limit**	**Cost amount**	**Lower limit**	**Upper limit**
Algeria	29928484	6753055	57317499	49593840	34589117	66396898	831052267	32363229	1666209241
Angola	8573438	1886973	17102444	2114695	1462346	2858315	148179595	5557139	309508855
Benin	416818	77138	857558	96579	66729	130717	5503925	205043	11587624
Botswana	374505	65713	882432	490994	339460	661685	23591394	892463	48840720
Burkina Faso	994771	183291	2104898	250615	173537	338352	13988365	526976	29075433
Burundi	1141736	167002	2609864	77544	53643	104718	4688608	175817	9793245
Cabo Verde	135208	7170	296220	38022	26334	51559	1820960	68283	3791811
Cameroon	3302760	839802	6029921	619874	431109	832513	39279546	1510545	79813575
Central Africa Republic	134978	31156	264098	20949	14454	28379	1748373	64835	3698424
Chad	703687	176861	1312152	184610	127963	248695	12277422	466060	25303917
Comoros (The)	68221	10642	197608	15309	10334	20602	633859	23441	1340586
Congo	642957	152478	1212750	254428	176887	341827	17283699	663839	35166770
Côte d'Ivoire	4206310	1042338	7669207	825053	575360	1104947	38468962	1496259	77227148
Democratic Republic of the Congo	5147222	1147916	10052799	485324	336657	653699	37384072	1420732	76941834
Equatorial Guinea	223853	56766	400749	121133	84054	162789	12348492	463007	25792039
Eritrea	250952	68358	471886	52697	36513	71059	4075423	154321	8420624
Eswatini	120221	28242	241137	221119	153981	297622	11083576	426602	22551658
Ethiopia	9484183	2298725	18167878	904776	624529	1225716	64483151	2395450	136162506
Gabon	514190	138986	944569	205014	142242	276038	13457429	513242	27583231
Ghana	7861329	1603307	15873553	1587601	1100003	2141241	77869604	2944512	161166962
Guinea	569861	56233	1213944	216146	149224	292761	11015431	408683	23288318
Guinea-Bissau	121234	31102	220099	29225	20107	39508	1671628	62672	3491830

Kenya	8720173	1198248	20347770	971775	670908	1315944	64249557	2390120	135467482
Lesotho	114300	14506	246164	40309	27455	55103	2571070	93451	5575747
Liberia	775296	171394	1462643	245665	170599	331104	13104594	499463	26873761
Madagascar	2823365	550967	5934055	327405	226498	442564	21364880	800671	44662219
Malawi	2502616	291004	6317210	374886	258817	507685	18652718	694150	39305227
Mali	2438026	530230	4679536	381774	267237	509130	15149989	599823	29861399
Mauritania	1211451	223340	2382092	263384	184093	350708	8702084	345506	17115063
Mauritius	1608573	353697	3127487	628096	435254	842897	20962257	808611	42444632
Mozambique	756273	112475	1869176	141996	97640	193759	9078074	329125	19731523
Namibia	1183186	281252	2187498	915334	638543	1225558	33089776	1288381	66378085
Niger	1608154	306981	3298746	172541	119621	232677	9335250	353518	19288661
Nigeria	52822654	13733880	95305799	14522523	10092644	19521119	684516576	26231253	1396147641
Rwanda	617119	124561	1241210	175491	121173	237780	13792078	511942	29149330
Sao Tome and Principe	36357	-838	77039	5594	3761	7651	220801	8165	465304
Senegal	1328472	258611	2654941	391678	271632	528088	15749597	597540	32478136
Seychelles	*	*	*	26276	18091	35910	640334	24054	1334581
Sierra Leone	793554	198616	1567064	549150	379017	744575	26448757	980692	55949229
South Africa	53163740	11383128	102193988	36189225	25301321	48316668	1348231890	53100164	2671878508
South Sudan	854499	156372	1829662	371107	256774	501402	22535626	845312	47070416
the Gambia	142193	19831	180276	36703	25404	49453	2218076	83727	4599973
Togo	198070	40848	401555	54410	37385	73969	2925052	107312	6273482
Uganda	813457	125915	1926411	175964	120882	240046	20188748	731644	43895540
United Republic of Tanzania	1968121	290284	4757198	279091	191758	380359	16016978	582016	34712546
Zambia	*	*	*	625261	433321	842972	38733493	1465816	80099039
Zimbabwe	*	*	*	707932	491710	951712	22619337	866252	46172035

	Cancers			Coronary Heart Disease			Depression		
**Region of the Americas**	**Cost amount**	**Lower limit**	**Upper limit**	**Cost amount**	**Lower limit**	**Upper limit**	**Cost amount**	**Lower limit**	**Upper limit**
Antigua and Barbuda	*	*	*	97314	68708	131539	1374390	53563	2745680
Argentina	79917745	18114349	148651846	44050051	30853399	58692937	1048037316	41615447	2059719867
Bahamas (The)	2816728	609516	5409193	655131	459144	871577	9361589	373724	18325407
Barbados	2938612	629713	5350527	791320	555018	1052789	8364739	333404	16387604
Belize	137817	31833	242111	122162	85134	163271	2209396	86552	4414326
Bolivia (Plurinational State of)	3601874	698482	6947560	777938	542816	1040790	70131905	2739737	140142947
Brazil	332651034	72113054	521960079	126950596	89170031	168629908	7881658480	317042379	15292672132
Canada	838906300	180840995	1619943153	115138390	80084298	154623453	1727244386	66437205	3508722437
Chile	263789348	43890345	531111701	14419020	10018597	19385659	474076296	18143794	968243278
Colombia	55778744	11224900	104588308	30909286	21675622	41125947	644785542	25752421	1259929271
Costa Rica	5460501	1109740	10129896	3218695	2258627	4275897	102179255	4100494	198672982
Cuba	17188816	3248807	33767008	42589847	29758338	56909040	705048059	27679356	1401780410
Dominica	*	*	*	52526	36786	70144	604601	23865	1182084
Dominican Republic (The)	7026371	1550508	13179351	7873245	5506301	10506660	161554961	6374521	319531064
Ecuador	8677384	1572294	17110434	1936990	1345709	2602669	162663417	6234651	331681526
El Salvador	3093018	534857	6060901	1282002	894705	1716042	45166958	1764622	90256065
Grenada	*	*	*	51483	35340	68974	713268	27107	1448402
Guatemala	7816073	1452834	14986887	2319445	1620722	3098832	121541668	4773846	241528601
Guyana	393803	86261	662965	241195	168293	322839	6300111	245866	12589528
Haiti	4920149	824371	9744399	1992288	1389984	2664487	48924656	1911379	97765400
Honduras	3168747	536049	6292162	1032883	721390	1382019	38790701	1515671	77514687
Jamaica	3151315	652115	6041005	1409805	982377	1888057	18232741	707850	36649882
Mexico	65648934	13705073	124873525	50743868	35300763	68133769	1850346720	71224716	3755779035

Nicaragua	2547189	495137	4870505	901627	629203	1205851	36299279	1417657	72535413
Panama	32968520	5707082	64197320	2294906	1601492	3070266	66673563	2605143	133232801
Paraguay	4755483	1017848	9002935	1162133	810727	1552012	89404831	3514839	177533070
Peru	26487907	4725132	51403069	3712090	2590960	4966295	193039847	7542098	385746107
Saint Kitts and Nevis	*	*	*	67444	47898	89799	1279152	49750	2574764
Saint Lucia	185714	35805	339125	117830	81742	156433	1568791	62242	3096444
Saint Vicent and the Grenadines	*	*	*	54338	38033	72350	739117	28541	1477460
Suriname	2188175	584383	4017542	5086699	3571545	6769280	100333552	4005602	195874401
Trinidad and Tobago	23522113	4083756	45857679	4085052	2855075	5455534	53675943	2115156	106373917
United States of America	6254431764	1283945571	12043673204	2357663820	1649936703	3144453298	61218938280	2421583192	120784063272
Uruguay	35425313	6963638	70570067	3982499	2761349	5369252	79645133	3017740	164562883
Venezuela (Bolivarian Republic of)	20558069	4384661	38947972	17782248	12387366	23838598	505560928	19582419	1019418415
	**Cancers**			**Coronary Heart Disease**			**Depression**		
**Eastern Mediterranean Region**	**Cost amount**	**Lower limit**	**Upper limit**	**Cost amount**	**Lower limit**	**Upper limit**	**Cost amount**	**Lower limit**	**Upper limit**
Afghanistan	6619998	1039498	9618905	4432446	3100841	5914271	126487101	4994914	249986762
Bahrain	24018464	4877795	48127594	3928713	2749547	5240914	84021690	3317488	166055995
Egypt	70138639	12790620	147237383	114309456	79610711	153282307	1486074638	57502263	2999681535
Iran (Islamic Republic of)	157276453	29513915	307302018	463446256	323148146	620613924	6439036559	250508449	12923170302
Iraq	21649208	5456632	39135095	22930537	16147585	30372477	404184462	16450938	775324621
Jordan	1979712	419723	3832648	2234275	1539495	3032461	44114315	1626152	94000647
Kuwait	95659532	20574454	177573058	22359480	15862565	29373340	467613912	19699170	868781252
Lebanon	9209269	1995690	17872293	21127186	14757738	28237580	287322556	11266860	571978607
Libya	5162044	1311414	9308894	11691546	8166674	15627082	223699149	8773099	445314220
Morocco	14563793	2963412	28775036	13695331	9513188	18419381	238749656	9128068	488147337
Oman	33396954	5781413	65226923	4803513	3348283	6432756	130070956	5057457	261249577

Pakistan	79313409	13331855	167141642	20936614	14602516	28028645	448787287	17481651	899559092
Qatar	7911634	1684765	15003232	5893849	4118787	7876118	224400679	8807227	446266940
Saudi Arabia	299023495	63840971	554318772	120783412	85101622	159885847	3166161677	129207437	6058550650
Somalia	2137600	489619	4076691	4650374	3253369	6203128	321092825	12679853	634602482
Sudan	12141649	2758436	23305984	10493105	7340871	14001178	226707864	8952596	448061313
Syrian Arab Republic	5407868	1203520	10328582	34539525	24160823	46084546	406656027	16059722	803707269
Tunisia	7645195	1430573	15703109	14615277	10175230	19605324	240140971	9279126	485496956
United Arab Emirates	27005936	6120976	50060966	18547991	12991718	24718344	492662050	19555161	968701897
Yemen	*	*	*	33968411	23762387	45321813	879995866	34748782	1739208434
Djibouti	460217	73908	927555	51812	36285	69153	2880716	113772	5693536
	**Cancers**			**Coronary Heart Disease**			**Depression**		
**European Region**	**Cost amount**	**Lower limit**	**Upper limit**	**Cost amount**	**Lower limit**	**Upper limit**	**Cost amount**	**Lower limit**	**Upper limit**
Albania	2990906	349542	5468375	1128947	785722	1515937	12787059	492345	25920131
Andorra	*	*	*	1331587	930261	1775438	32976959	1299953	65320628
Armenia	2877633	470433	4979126	2959791	2052601	3989632	21790565	825434	44998389
Austria	384031179	69902225	660427531	45629521	31758847	61223608	600985558	23203034	1215975841
Azerbaijan	9628087	1266623	17665869	10448175	7269850	14024445	83862818	3232458	169995185
Belarus	10965550	1812481	20137770	12283521	8476266	16651335	115060884	4267089	243547148
Belgium	612955464	105598011	993566199	36530373	25503356	48846538	847816560	33186550	1690733135
Bosnia and Herzegovina	4972525	851645	8469793	2945257	2045628	3962643	32260027	1231548	66084858
Bulgaria	14160424	2321972	22864086	16184951	11317433	21602474	64172851	2529613	127050405
Croatia	49492801	8238731	82938410	6446680	4488637	8643017	60088959	2325861	121259221
Cyprus	22286789	4186083	27224193	1581000	1109324	2103749	33942614	1355538	66263426
Czechia	212973332	39587850	365111947	17443876	12149590	23389785	225208547	8716300	454469970
Denmark	222177016	39922030	396554366	13078047	9095563	17561525	387160665	14882348	786690333

Estonia	19880206	3463053	34342041	3310192	2306570	4436426	29728161	1151743	59849818
Finland	65566797	12072273	213300753	15301250	10572568	20703078	167573889	6252892	352070287
France	2494567191	479378471	4444725539	147832586	102863184	198445727	3596457141	138574041	7292211605
Georgia	4309862	704090	11565947	2936360	2030381	3970461	24004331	898983	50227355
Germany	5304883288	857916189	6702821886	573453239	401779005	763831841	7411482123	294729221	14544781780
Greece	187019083	28095835	246418376	34985691	24452594	46723586	342698408	13480018	679992933
Hungary	150768830	25997859	207011342	24293766	16987615	32430622	230277765	9077352	456019328
Iceland	12533669	2231082	21997491	936749	652410	1259888	14005064	537334	28401041
Ireland	174264021	32245583	290175368	11628053	8105606	15578335	296850991	11533878	596548051
Israel	266812381	43898451	462847618	8898699	6191552	11942962	298942512	11520707	605976894
Italy	2504868561	389958681	3114867737	288749421	202220904	384791752	3330520654	132187266	6548685861
Kazakhstan	21786734	3413133	47263024	15897683	11049284	21361221	196721600	7545852	400800857
Kyrgyzstan	1032675	117323	4457092	589809	407069	799604	7645585	283284	16192854
Latvia	25836073	4149426	47389781	2431569	1692217	3264621	34232287	1319121	69370644
Lithuania	41153877	6570504	87014976	4722704	3282212	6350417	44838412	1715317	91601822
Luxembourg	26211386	4738468	49725290	1706595	1191471	2291160	51915218	2003155	105523352
Malta	17743683	3001483	22608181	797376	555990	1061582	22194896	880921	43609408
Monaco	*	*	*	74830	52135	101457	1583412	61089	3206552
Montenegro	895514	150917	1556410	1250338	867006	1678041	15255702	587073	30926051
Netherlands	903750671	149178949	1492566290	113143171	78634334	152077665	1160590335	44485926	2366518075
North Macedonia	27563021	4146989	46877688	1482540	1030977	1989700	18089524	696529	36669040
Norway	256525567	46804637	420084462	21122648	14714995	28299452	400539752	15525477	807007899
Poland	497684492	78447465	825778438	38044322	26515951	50967216	645239531	25060915	1297344941
Portugal	230891895	39570570	293265955	11956028	8384231	15916383	1886920579	75251371	3692371492
Republic of Moldova	2424773	373962	11715216	1350659	930877	1833790	12571730	462990	26821135

Romania	44341751	7737241	65079560	14870385	10381316	19886829	208858561	8168847	416828864
Russian Federation	142804535	28700708	541920903	186340323	128776665	252068262	1536992019	57423232	3224432260
San Marino	*	*	*	47600	32456	63482	1233824	45513	2497448
Serbia	21684849	3776344	28109171	20431102	14292398	27255835	166924295	6594868	329744363
Slovakia	108270184	18915576	127266796	12163479	8488064	16270721	88457163	3456947	176760267
Slovenia	45778925	8069544	75850142	2576935	1796265	3452357	62637601	2433887	126038823
Spain	822866961	137350468	1199648964	98874201	68701291	132927876	1616781831	61910907	3300288078
Sweden	200050910	34889926	440422482	15968046	11070202	21517983	574781814	21803577	1185290996
Switzerland	440215891	57377757	942471971	31612381	21931580	42585682	703260079	26722212	1447830132
Tajikistan	1794931	219593	3760879	1376644	958139	1848055	15553018	599237	31535036
Turkey	154798094	25085274	257565401	107264837	74688028	143873041	2261430812	87417995	4569554778
Turkmenistan	3050970	411983	6426145	4448423	3095957	5971281	44566270	1717661	90336246
Ukraine	52417989	7615607	149269526	60304167	41734406	81444406	485202292	18244710	1010537331
United Kingdom of Great Britain and Northern Ireland (the)	8214790735	1622813490	12728680824	205673151	143613708	274974602	5612516807	219811218	11186956016
Uzbekistan	6621686	951485	19487939	7018776	4856000	9481798	72219374	2712287	150629732
	**Cancers**			**Coronary Heart Disease**			**Depression**		
**South-East Asia Region**	**Cost amount**	**Lower limit**	**Upper limit**	**Cost amount**	**Lower limit**	**Upper limit**	**Cost amount**	**Lower limit**	**Upper limit**
Bangladesh	82258213	7247649	201521203	10048215	6985807	13500783	251540938	9656047	512077194
Bhutan	47243	0	116095	122996	122996	122996	1945161	1945161	1945161
Democratic People's Republic of Korea	9097868	1769764	17948930	3491713	2420099	4708219	67340707	2548148	139257709
India	703279845	111229448	1462287279	273265771	190624185	365765696	4588575047	178870697	9190360183
Indonesia	53981671	13366245	71702086	9687978	6716032	13059029	426224983	16149357	880176057
Maldives	212577	51426	395490	110835	77054	147438	4740832	182572	9586837
Myanmar	6158481	760196	14201629	1376440	947690	1869683	34674162	1274105	74158710
Nepal	1364976	204983	2919731	1441144	993723	1954065	42255846	1563749	89630583

Sri Lanka	14872692	2210251	31798837	4040222	2810968	5424510	87440379	3365678	177483891
Thailand	40587098	8748566	78125097	11505321	7985314	15489253	306326341	11664966	629069536
Timor-Leste	51688	6867	95508	43874	30616	59281	1428282	53591	2990290
	**Cancers**			**Coronary Heart Disease**			**Depression**		
**Western Pacific Region**	**Cost amount**	**Lower limit**	**Upper limit**	**Cost amount**	**Lower limit**	**Upper limit**	**Cost amount**	**Lower limit**	**Upper limit**
Australia	704238585	154727240	1210095857	125562505	87422828	168436391	1761302948	68047245	3560838895
Brunei Darussalam	1692631	298733	3112445	146089	100784	195342	3655331	140562	7452425
Cambodia	1672169	281586	3171156	282846	194669	384340	10627101	390245	22742571
China	1100041686	151129676	2275213837	241613315	166687448	327481141	4737789617	175639608	10028320844
Cook Islands	*	*	*	7016	5078	9758	113330	3418	234878
Fiji	198597	22331	369715	55758	38296	75068	1323807	49479	2774808
Japan	8815622997	1171296735	16581560101	413054918	288370625	552374272	5314148	207916	10602968
Kiribati	*	*	*	274474	191931	365715	9695657	383932	19110694
Lao People's Democratic Republic	803417	144697	1537514	833262	575510	1127807	28529185	1063639	59992152
Malaysia	23478496	5027083	39987275	12354665	8640947	16490691	415547919	16390524	822292894
Marshall Islands	*	*	*	10844	7572	13969	338734	13517	663908
Micronesia (Federated States of)	1546961	289118	2826870	33371	23542	45307	847856	33750	1688493
Mongolia	1382510	73964	3274414	944619	653170	1277068	16057404	602404	33538699
Nauru	*	*	*	4399	1564	6768	146426	6231	290746
New Zealand	113260509	25457287	195523708	17576184	12316845	23406912	234674313	9338113	460314532
Niue	*	*	*	1262	2063	0	20152	7	40606
Palau	*	*	*	19677	13331	26662	453588	16782	893767
Papua New Guinea	1584515	268124	3132227	84417	58318	114355	3229502	120011	6821499
Philippines	59542204	14343061	102614749	15571429	10895714	20771636	340484091	13458503	672264425
Republic of Korea	372631426	41625966	720391956	51697552	36091436	69139359	1295366366	50667821	2585216266

Samoa	61988	7311	106716	8147	5821	11104	191654	7144	407332
Singapore	73266529	15229980	129667914	10439589	7295079	13953897	186194148	7301331	370569288
Solomon Islands	26972	2659	66380	8733	6117	11826	313745	11780	656202
Tonga	*	*	*	6207	4124	8325	117521	4317	246174
Tuvalu	*	*	*	3329	1773	4070	76236	3506	150798
Vanuatu	28299	1328	28629	2627	1735	3556	70701	2522	152611
Viet Nam	39260045	6849635	75173284	8309991	5769661	11182131	187106512	7139323	383395521

*No estimates

(continued from previous table)

	**Hypertension**			**Stroke**			**Type 2 Diabetes**		
**African Region**	**Cost amount**	**Lower limit**	**Upper limit**	**Cost amount**	**Lower limit**	**Upper limit**	**Cost amount**	**Lower limit**	**Upper limit**
Algeria	319038872	161111593	473876634	49399214	24161511	70787895	231351524	152606717	307142162
Angola	37741426	18975521	56301333	5520662	2665396	8004917	15192086	9934241	20335942
Benin	2138138	1074075	3192058	241874	116428	351560	46702	30508	62666
Botswana	10562146	5314746	15742152	1400393	675160	2027989	454203	297414	607472
Burkina Faso	6687298	3363867	9970910	667087	322411	966104	347656	227693	464959
Burundi	2303549	1158161	3436363	216106	104047	313389	125347	82098	167801
Cabo Verde	*	*	*	90511	43981	131216	3674	2403	4923
Cameroon	12120697	6111776	18029523	1703165	829428	2450289	3946717	2596150	5254218
Central Africa Republic	1181424	593288	1764648	91214	43777	132869	81558	53200	109337
Chad	3919031	1973114	5838345	415425	201196	600316	274952	180271	366979
Comoros (The)	289465	145340	432394	38825	18416	56294	2277	1507	3047
Congo	665281	335399	989743	529429	257885	762248	247666	163004	330148
Côte d'Ivoire	20493139	10347416	30443317	2516374	1230254	3607950	2118246	1396896	2812817
Democratic Republic of the Congo	9060739	4562473	13496099	5271578	2555054	7611999	2744396	1800543	3662786

Equatorial Guinea	3146802	1582408	4694934	236708	115592	343135	59458	39053	79946
Eritrea	822114	413752	1225045	123855	59926	178934	36036	23587	48131
Eswatini	4583154	2310520	6817971	563115	278021	812714	48926	32314	65254
Ethiopia	30873008	15505163	46105225	1702195	818236	2476157	9290434	6064256	12460118
Gabon	4703826	2369540	7003035	494750	240576	711778	226654	148764	301738
Ghana	1801616	906830	2684997	3773339	1825338	5456574	3586092	2349699	4791880
Gambia (The)	*	*	*	95883	46407	138508	3790	2482	5071
Guinea	10302026	5173339	15386720	500368	240534	727678	132755	86655	178140
Guinea-Bissau	4306533	2165225	6424254	83757	40088	121367	4211	2763	5644
Kenya	1092571	548647	1631526	2369065	1139032	3444698	7785574	5084068	10438878
Lesotho	17320702	8676587	25932137	122936	58178	180306	18427	12051	24847
Liberia	2118449	1067229	3154315	486536	236173	701803	94609	62134	126137
Madagascar	1437223	722469	2144091	1446286	697392	2098259	729401	477049	976154
Malawi	13839261	6951901	20663333	870695	418228	1264035	976694	637779	1309522
Mali	8421553	4261221	12484650	979860	482172	1396647	507772	336130	671802
Mauritania	23463968	11875535	34775416	552893	271943	787752	46910	31099	62038
Mauritius	34439303	17372641	51209915	1977256	967717	2840516	1007933	663906	1340912
Mozambique	398151	199326	596215	625056	297832	916557	488952	317279	659127
Namibia	149609603	75546865	222231541	1738340	849853	2493742	595793	393286	791923
Niger	741421	373165	1104780	476275	230828	688805	455812	298851	608799
Nigeria	42890205	21618042	63824865	33003857	16048618	47537273	379583900	249477668	505689781
Rwanda	74577933	37451598	111383128	502714	241653	731934	367753	240132	493142
Sao Tome and Principe	7467593	3750932	11149945	25677	12993	38742	541	349	746
Senegal	96705	48715	144137	856806	415173	1237892	771294	505803	1030061
Seychelles	73047565	36728410	108963519	124254	63101	175626	1583	1025	2118

Sierra Leone	182304	91524	271906	1262789	607503	1835031	133293	87242	178961
South Africa	64043663	32384372	95000096	72968170	35843898	104201591	1163847610	769668516	1541453700
South Sudan	32661259	16421193	48723089	493160	237327	715175	1402081	917040	1877699
Togo	1256157	629927	1878757	154712	74159	226383	25537	16621	34369
Uganda	4947069	2477534	7408369	469101	223556	688946	1139916	739669	1536780
United Republic of Tanzania	6562083	3287469	9824053	721090	343738	1056722	3759540	2441303	5067589
Zambia	16828687	8469830	25078970	1507141	729491	2179490	3860525	2530635	5157910
Zimbabwe	12175726	6136358	18120142	1417833	689418	2042992	1517667	997299	2021734
	**Hypertension**			**Stroke**			**Type 2 Diabetes**		
**Region of the Americas**	**Cost amount**	**Lower limit**	**Upper limit**	**Cost amount**	**Lower limit**	**Upper limit**	**Cost amount**	**Lower limit**	**Upper limit**
Antigua and Barbuda	1067604	539759	1585506	93162	44656	132703	2721	1818	3607
Argentina	750802669	380028806	1112656184	78662562	38757644	112030493	249674790	165412179	330105766
Bahamas (The)	6441687	3261774	9541627	768972	384653	1098131	86796	57504	114667
Barbados	4742640	2401613	7025462	666186	330504	950393	65246	43160	86427
Belize	895455	452521	1329838	88481	44241	126793	19411	12843	25792
Bolivia (Plurinational State of)	24564616	12409565	36472610	3124027	1529257	4470142	6784189	4478105	9000170
Brazil	3872242711	1963041165	5729792043	471014120	233171970	668182710	29890929542	19859924313	39411650274
Canada	1010266986	509432925	1502727705	127655712	62162400	183648132	810397271	533102795	1078754838
Chile	272351376	137251914	405340500	26003275	12628605	37456319	112663510	74031671	150118059
Colombia	476517959	241363124	705701011	47884922	23639434	68081489	1006245527	667500617	1328771335
Costa Rica	52709213	26714113	78015392	4563843	2253639	6476737	16611313	11032259	21913288
Cuba	240339786	121484855	356647093	32928827	16154123	47061901	84686464	55967161	112240477
Dominica	*	*	*	50325	24595	70933	420177	275793	558206
Dominican Republic (The)	77318315	39106147	114666024	10982691	5398048	15674612	23770275	15725407	31466037
Ecuador	58611802	29543094	87217288	7432917	3617295	10708444	30263732	19891435	40312929

El Salvador	18341785	9265433	27233227	1894575	927489	2714432	4310950	2844878	5717377
Grenada	459891	232043	684216	56178	26740	80908	3388	2229	4513
Guatemala	43008476	21741183	63817888	4081484	2000834	5831094	49299777	32584496	65333112
Guyana	2317755	1171029	3441336	3235734	1585642	4633316	102506	67673	135998
Haiti	18184422	9186302	26999501	3262493	1598160	4670710	7902026	5215899	10483610
Honduras	17967528	9076781	26677496	1487414	727148	2128290	4472595	2953008	5934400
Jamaica	12332589	6226135	18323205	1676402	820057	2403623	1972300	1299831	2620955
Mexico	722471651	364341540	1074551365	74243211	36158379	106782753	7998265011	5262432173	10645365545
Nicaragua	17345853	8762693	25754910	1528214	746377	2187897	3510765	2317430	4657301
Panama	41684347	21059644	61892119	4540507	2218981	6501559	3470176	2290825	4603469
Paraguay	47199136	23861744	70030193	5494614	2698103	7854907	5207339	3442217	6900151
Peru	96268197	48633048	142937741	12473996	6107301	17857271	79467584	52461859	105437383
Saint Kitts and Nevis	*	*	*	83620	57663	125405	1232	810	1620
Saint Lucia	1115975	564631	1654367	129066	62736	181052	12106	8042	16027
Saint Vicent and the Grenadines	456302	230709	677520	61447	30775	88154	2681	1748	3551
Suriname	41860337	21203322	61990148	5750553	2829381	8183890	1240397	821849	1639601
Trinidad and Tobago	30875400	15612465	45799430	3523530	1724627	5022753	1302668	861679	1724974
United States of America	23435875118	11856883035	34746638055	3806526957	1872729050	5427973904	469832310001	310997757499	621684169759
Uruguay	58375156	29381350	86982187	7919695	3844508	11457275	2387668	1566580	3193041
Venezuela (Bolivarian Republic of)	238100353	120161148	353879744	30404138	14842601	43644118	33666607	22181401	44748480
	**Hypertension**			**Stroke**			**Type 2 Diabetes**		
**Eastern Mediterranean Region**	**Cost amount**	**Lower limit**	**Upper limit**	**Cost amount**	**Lower limit**	**Upper limit**	**Cost amount**	**Lower limit**	**Upper limit**
Afghanistan	43132636	21817548	63962195	6869567	3377245	9800979	18962327	12548070	25100702
Bahrain	18214634	9214516	27010626	2243713	1102812	3209459	1821952	1205936	2411533
Djibouti	970770	491040	1439617	118492	58662	169120	20733	13669	27405

Egypt	710939281	358745280	1056762184	134324049	65546786	192868432	578566758	381091950	769179768
Iran (Islamic Republic of)	1977144125	998319172	2937034440	323067442	157964986	463106839	3798686910	2505145682	5044289009
Iraq	185420608	94134541	273985710	42644324	21202054	60280980	240991736	160539107	316956379
Jordan	11312133	5675907	16908299	2993797	1435379	4366886	8368130	5450529	11237511
Kuwait	179620183	91577797	264320633	20364966	10264435	28469148	47667821	31999937	62253308
Lebanon	133058259	67248784	197476334	17620124	8637261	25202365	31829564	21032610	42201579
Libya	77181771	39007130	114548676	13250890	6497124	18941480	52351435	34583153	69405770
Morocco	95601968	48173872	142301705	15495212	7527357	22333455	76783118	50441922	102344041
Oman	40434344	20414725	60068506	3970554	1946792	5695648	3688592	2432438	4900558
Pakistan	332486664	167907239	493836295	27325849	13367041	39153297	1161348424	766113432	1541769300
Qatar	51766384	26165613	76818202	4560111	2244780	6525894	13625472	9006005	18063063
Saudi Arabia	1095326296	556249920	1618006666	164420297	81826659	232214029	1318455796	878768822	1733115765
Somalia	120468870	60936026	178646152	15829595	7780155	22577733	25266719	16723207	33448172
Sudan	80470537	40703999	119331088	70890348	34854917	101131564	51822010	34291173	68597861
Syrian Arab Republic	238978098	120881449	354384451	33125510	16285604	47263990	86977859	57550471	115130664
Tunisia	52294875	26383699	77745625	14191393	6923911	20383044	23655207	15574346	31456622
United Arab Emirates	312175587	158004888	462656742	37050972	18247463	52770709	109400429	72471649	144653359
Yemen	*	*	*	45980444	22607110	65603161	81068265	53646449	107305604
	**Hypertension**			**Stroke**			**Type 2 Diabetes**		
**European Region**	**Cost amount**	**Lower limit**	**Upper limit**	**Cost amount**	**Lower limit**	**Upper limit**	**Cost amount**	**Lower limit**	**Upper limit**
Albania	19241121	9704473	28613464	2512052	1226060	3614013	549984	361890	731781
Andorra	*	*	*	1722124	843553	2460014	34230	22592	45339
Armenia	18951358	9540431	28239656	2106897	1021826	3049705	689246	451907	920629
Austria	473588735	238913624	704136226	61407342	29940880	88222718	50363819	33153875	66993660
Azerbaijan	53164559	26814433	79061864	9338775	4549523	13430697	4459726	2934673	5933886

Belarus	69976478	35136287	104526013	12381790	5950398	18024058	3029670	1980037	4067821
Belgium	554938077	280410843	823769622	47944858	23502753	68598419	65042675	42954096	86254534
Bosnia and Herzegovina	42416651	21369261	63149906	7003994	3399884	10102180	3086124	2026045	4115177
Bulgaria	187403417	94774949	277958592	41118811	20203641	58703127	19404846	12830359	25696280
Croatia	107161353	54075556	159283541	12027405	5870589	17277305	2803102	1846615	3726015
Cyprus	29537315	14963356	43737986	1007728	496794	1430995	1073734	712444	1417230
Czechia	396269532	199966843	589012797	35378053	17265869	50793962	6598558	4351868	8773469
Denmark	261653740	131935169	389211342	11358785	5527719	16328282	20823949	13699223	27712372
Estonia	39046511	19707037	58023021	5651009	2754612	8109069	305401	201241	405879
Finland	113098819	56831093	168816896	26109693	12576092	37924137	15406390	10061219	20641577
France	2900193402	1462731628	4313033095	152621828	74356443	219432591	1681749396	1106693545	2237753167
Georgia	22053153	11086286	32904384	3446146	1660606	5002163	969102	633792	1297344
Germany	6449199026	3264912702	9555808028	884492882	435981548	1259177030	14374335417	9526086818	18998691352
Greece	251156396	126983653	372613717	29177206	14324851	41674212	29350236	19400198	38880066
Hungary	341142054	172517761	506000532	34299549	16847440	48964477	32155623	21266531	42581646
Iceland	12385996	6246821	18417038	1073629	528196	1553893	98740	64980	132116
Ireland	193404733	97643188	287343712	11596614	5686544	16625692	9326395	6148946	12386472
Israel	119856596	60453053	178243052	13197326	6431058	18972964	35912266	23633075	47767781
Italy	2979143742	1507844710	4415205669	346480668	170679983	493572817	3158660975	2092331267	4176490630
Kazakhstan	133606536	67350175	198796030	22077696	10739098	31789663	27092332	17812146	36086088
Kyrgyzstan	4459423	2238989	6661567	552002	265389	802769	158896	103803	213288
Latvia	45905450	23153980	68265224	3476747	1694387	4996729	427478	281073	569183
Lithuania	71477186	36021321	106384511	6410465	3118221	9237229	729556	479681	972127
Luxembourg	27200043	13713199	40455590	1942597	931485	2789647	462449	304292	615801
Malta	19063905	9647812	28251360	1087130	539111	1546893	175507	116282	231992

Monaco	*	*	*	124470	57777	176983	635	419	913
Montenegro	*	*	*	2181588	1064388	3133769	6024148	3962566	8010977
Netherlands	791894389	399154092	1178383594	73216583	35605391	105434733	154229639	101382258	205450557
North Macedonia	*	*	*	4837596	2356809	6960137	808187	532178	1075494
Norway	291353802	147047408	432993294	33095412	16155206	47498828	23440707	15450921	31154831
Poland	958668858	483962511	1424381043	60173560	29397323	86298933	258195966	170210891	342991628
Portugal	492183579	249252962	729022818	26070589	12867127	37073430	98799049	65519405	130503766
Republic of Moldova	9861314	4947729	14741106	1338048	639934	1953875	277405	180328	372339
Romania	446940984	225819493	663512106	69337883	33969044	99227968	42582459	28116153	56478211
Russian Federation	1485257651	746419590	2216631646	260657397	125552896	378490026	1449556689	947388847	1941748386
San Marino	*	*	*	64041	33310	97333	220809	143460	298157
Serbia	201330827	101844196	298538636	34611534	17014781	49374612	36761704	24327803	48656717
Slovakia	151017202	76290561	224225652	18299371	8963008	26197428	6638228	4381612	8806971
Slovenia	87318473	44079847	129748373	4354532	2127819	6244073	2399171	1579900	3185952
Spain	1210329762	609990240	1801243467	110618299	53774881	159369698	1555231322	1022030038	2072303440
Sweden	367249073	184886230	547135969	37785779	18291868	54589166	54408256	35684410	72645513
Switzerland	390200361	196477078	581219833	35974990	17444636	51979889	63632174	41749767	84921468
Tajikistan	12762411	6436800	18979699	1307661	637357	1881141	876154	576459	1165962
Turkey	948250526	478437217	1409668830	165905340	80927087	238286992	2658295088	1750665610	3534751869
Turkmenistan	41113613	20735958	61140319	7478301	3642902	10747370	2460767	1619980	3275364
Ukraine	341430409	171712986	509185549	51097502	24667170	74042255	75772222	49587230	101359244
United Kingdom of Great Britain and Northern Ireland (the)	2390062461	1207765686	3547691260	232961935	114184579	333211716	5053749703	3337809116	6701314343
Uzbekistan	55253168	27784027	82412282	7596432	3665131	11010819	9446055	6180848	12640447
	**Hypertension**			**Stroke**			**Type 2 Diabetes**		
**South-East Asia Region**	**Cost amount**	**Lower limit**	**Upper limit**	**Cost amount**	**Lower limit**	**Upper limit**	**Cost amount**	**Lower limit**	**Upper limit**

Bangladesh	104535450	52700078	155527408	12573752	6117440	18099688	217838608	143238400	290107877
Bhutan	4254194	4254194	4254194	101494	49683	144605	8786	8786	8786
Democratic People's Republic of Korea	54278950	27318069	80889320	20272133	9811847	29316204	50833266	33316379	67912377
India	2923311758	1476414255	4341568849	257165544	125834761	368391855	26653830678	17585733702	35378757967
Indonesia	472111396	237645357	703461540	116854817	56577867	168912820	1918918595	1258092330	2562711471
Maldives	3845213	1940030	5716322	323512	158506	459956	33186	21937	44129
Myanmar	42537574	21336873	63603448	4852630	2323064	7084226	19535859	12721220	26264908
Nepal	16344821	8205088	24419613	994482	477344	1449315	2447730	1596152	3285613
Sri Lanka	81821058	41262057	121694432	11454620	5578426	16473830	25668172	16888775	34162669
Thailand	307985109	155120313	458641386	41524602	20141579	59925563	20702861	13588994	27619090
Timor-Leste	1116454	561350	1665938	239916	115435	346286	13223	8659	17772

	**Hypertension**			**Stroke**			**Type 2 Diabetes**		
**Western Pacific Region**	**Cost amount**	**Lower limit**	**Upper limit**	**Cost amount**	**Lower limit**	**Upper limit**	**Cost amount**	**Lower limit**	**Upper limit**
Australia	1059720159	534645218	1575462618	75612790	36889995	108638225	352808544	232296357	469211400
Brunei Darussalam	5800918	2923191	8629596	496191	243772	717464	48929	32121	65132
Cambodia	12271405	6155149	18349924	1401928	670462	2046874	1549932	1009871	2084116
China	5322057198	2672237283	7949739696	1035991381	497568006	1508089207	183114180040	119472777169	245712842445
Cook Islands	171640	86134	256717	21448	9539	29871	2405	1616	3219
Fiji	1465564	736489	2187135	164792	78956	239544	137304	89691	183795
Japan	9427856	4763603	13995838	1236341743	605779795	1769011724	7263173550	4796068409	9633233968
Kiribati	11233129	5683563	16652657	2014268	991076	2871399	99505	65883	131625
Lao People's Democratic Republic	25380496	12752094	37887390	3014992	1451267	4381556	1574887	1028922	2110632
Malaysia	386788430	195619263	573655701	40957159	20129738	58462775	374521379	247746208	495885317
Marshall Islands	721243	364875	1067948	57501	27879	80974	4959	3305	6613
Micronesia (Federated States of)	1091910	551687	1621663	160658	82828	230841	3143477	2084404	4168422

Mongolia	12614882	6342283	18818561	1446086	697661	2099715	141309	92278	189246
Nauru	290217	147309	430953	19284	6126	19456	53	39	69
New Zealand	215474349	109088220	319248886	13862141	6843978	19727572	8802460	5835597	11632262
Niue	42710	21926	64249	*	*	*	43	21	55
Palau	*	*	*	78072	39104	106276	399	262	520
Papua New Guinea	2835825	1424178	4235132	448716	215933	652799	1622592	1059086	2176415
Philippines	363278394	183776916	538651279	49158796	24178490	70114330	375756596	248688802	497286477
Republic of Korea	*	*	*	195194084	95635947	279327895	1196998061	790363573	1587623710
Samoa	226747	113980	339167	30740	15753	44967	967	653	1312
Singapore	221141458	111767499	328195414	22688370	11136420	32454181	24149512	15955044	32011858
Solomon Islands	268455	135059	400563	41165	19950	59398	12063	7906	16189
Tonga	162406	81551	242251	18283	8742	26255	753	513	1026
Tuvalu	147525	74156	217989	10781	5841	15975	126	75	158
Vanuatu	70191	35114	104871	7950	3927	11507	1073	716	1490
Viet Nam	318262616	160334758	473838139	48057220	23329512	69308036	259535130	170423094	346080614

*No cost estimates

Table S9. Direct healthcare costs attributable to physical inactivity, by health outcomes and by country (in 1,000,000 INT$), 2020-2030

	Cancers	Coronary Heath Disease	Depression	Hypertension	Stroke	Type 2 Diabetes
**African Region**						
Algeria	102 (23·1 to 196·3)	170 (118·4 to 227·4)	2846 (110·8 to 5705·9)	1093 (551·7 to 1622·8)	169 (82·7 to 242·4)	792 (522·6 to 1051·8)
Angola	31 (6·8 to 62·1)	8 (5·3 to 10·4)	538 (20·2 to 1123·2)	137 (68·9 to 204·3)	20 (9·7 to 29)	55 (36 to 73·8)
Benin	1 (0·2 to 2·3)	0* (0·2 to 0·4)	15 (0·6 to 31·4)	6 (2·9 to 8·6)	1 (0·3 to 1)	0* (0·1 to 0·2)
Botswana	1 (0·2 to 2·1)	1 (0·8 to 1·6)	57 (2·2 to 117·9)	25 (12·8 to 38)	3 (1·6 to 4·9)	1 (0·7 to 1·5)
Burkina Faso	3 (0·5 to 5·6)	1 (0·5 to 0·9)	37 (1·4 to 77·1)	18 (8·9 to 26·4)	2 (0·9 to 2·6)	1 (0·6 to 1·2)
Burundi	3 (0·5 to 7·3)	0* (0·2 to 0·3)	13 (0·5 to 27·6)	6 (3·3 to 9·7)	1 (0·3 to 0·9)	0* (0·2 to 0·5)
Cabo Verde	0* (0 to 0·6)	0* (0·1 to 0·1)	4 (0·1 to 7·9)	0* (0 to 0)	0* (0·1 to 0·3)	0* (0 to 0)
Cameroon	8 (2·1 to 15·2)	2 (1·1 to 2·1)	99 (3·8 to 200·8)	30 (15·4 to 45·4)	4 (2·1 to 6·2)	10 (6·5 to 13·2)
Central Africa Republic	0* (0·1 to 0·5)	0* (0 to 0·1)	4 (0·1 to 7·4)	2 (1·2 to 3·5)	0 (0·1 to 0·3)	0* (0·1 to 0·2)
Chad	2 (0·4 to 3·2)	0* (0·3 to 0·6)	30 (1·1 to 61·5)	10 (4·8 to 14·2)	1 (0·5 to 1·5)	1 (0·4 to 0·9)
Comoros (The)	0* (0 to 0·4)	0* (0 to 0)	1 (0·1 to 3)	1 (0·3 to 1)	0* (0 to 0·1)	0* (0 to 0)
Congo	1 (0·3 to 2·4)	0* (0·3 to 0·7)	34 (1·3 to 69)	1 (0·7 to 1·9)	1 (0·5 to 1·5)	0* (0·3 to 0·6)
Côte d'Ivoire	10 (2·4 to 18)	2 (1·4 to 2·6)	90 (3·5 to 181·5)	48 (24·3 to 71·5)	6 (2·9 to 8·5)	5 (3·3 to 6·6)
Democratic Republic of the Congo	11 (2·4 to 21·1)	1 (0·7 to 1·4)	78 (3 to 161·5)	19 (9·6 to 28·3)	11 (5·4 to 16)	6 (3·8 to 7·7)
Equatorial Guinea	1 (0·1 to 1)	0* (0·2 to 0·4)	31 (1·2 to 64·8)	8 (4 to 11·8)	1 (0·3 to 0·9)	0* (0·1 to 0·2)
Eritrea	No estimates in INT$**					
Eswatini	0* (0·1 to 0·6)	1 (0·4 to 0·8)	29 (1·1 to 58·4)	12 (6 to 17·7)	1 (0·7 to 2·1)	0* (0·1 to 0·2)
Ethiopia	27 (6·6 to 52·4)	3 (1·8 to 3·5)	186 (6·9 to 392·6)	89 (44·7 to 132·9)	5 (2·4 to 7·1)	27 (17·5 to 35·9)
Gabon	1 (0·3 to 2·1)	0* (0·3 to 0·6)	30 (1·1 to 60·5)	10 (5·2 to 15·4)	1 (0·5 to 1·6)	0* (0·3 to 0·7)
Ghana	20 (4·2 to 41·3)	4 (2·9 to 5·6)	203 (7·7 to 419·8)	5 (2·4 to 7)	10 (4·8 to 14·2)	9 (6·1 to 12·5)
Gambia (The)	0* (0·1 to 0·5)	0* (0·1 to 0·1)	7 (0·2 to 13·5)	†	0* (0·1 to 0·4)	0* (0 to 0)
Guinea	1 (0·1 to 2·9)	1 (0·4 to 0·7)	26 (1 to 54·9)	24 (12·2 to 36·3)	1 (0·6 to 1·7)	0* (0·2 to 0·4)
Guinea-Bissau	0* (0·1 to 0·6)	0* (0·1 to 0·1)	4 (0·2 to 9·4)	12 (5·8 to 17·2)	0* (0·1 to 0·3)	0* (0 to 0)
Kenya	21 (2·9 to 49·6)	2 (1·6 to 3·2)	157 (5·8 to 330·1)	3 (1·3 to 4)	6 (2·8 to 8·4)	19 (12·4 to 25·4)
Lesotho	0* (0 to 0·7)	0* (0·1 to 0·2)	7 (0·3 to 15·6)	48 (24·2 to 72·3)	0* (0·2 to 0·5)	0* (0 to 0·1)
Liberia	2 (0·4 to 3·8)	1 (0·4 to 0·9)	34 (1·3 to 69·7)	5 (2·8 to 8·2)	1 (0·6 to 1·8)	0* (0·2 to 0·3)
Madagascar	9 (1·8 to 19·4)	1 (0·7 to 1·4)	70 (2·6 to 146·3)	5 (2·4 to 7)	5 (2·3 to 6·9)	2 (1·6 to 3·2)
Malawi	6 (0·7 to 15·8)	1 (0·6 to 1·3)	47 (1·7 to 98·2)	35 (17·4 to 51·6)	2 (1 to 3·2)	2 (1·6 to 3·3)

Mali	7 (1·4 to 12·7)	1 (0·7 to 1·4)	41 (1·6 to 81·3)	23 (11·6 to 34)	3 (1·3 to 3·8)	1 (0·9 to 1·8)
Mauritania	4 (0·7 to 7·5)	1 (0·6 to 1·1)	28 (1·1 to 54·2)	74 (37·6 to 110·1)	2 (0·9 to 2·5)	0* (0·1 to 0·2)
Mauritius	4 (0·8 to 7·4)	1 (1 to 2)	50 (1·9 to 101)	82 (41·3 to 121·9)	5 (2·3 to 6·8)	2 (1·6 to 3·2)
Mozambique	2 (0·3 to 5·4)	0* (0·3 to 0·6)	26 (1 to 57)	1 (0·6 to 1·7)	2 (0·9 to 2·6)	1 (0·9 to 1·9)
Namibia	3 (0·6 to 4·9)	2 (1·4 to 2·7)	74 (2·9 to 147·7)	333 (168·1 to 494·4)	4 (1·9 to 5·5)	1 (0·9 to 1·8)
Niger	4 (0·7 to 7·5)	0* (0·3 to 0·5)	21 (0·8 to 43·8)	2 (0·8 to 2·5)	1 (0·5 to 1·6)	1 (0·7 to 1·4)
Nigeria	131 (34·2 to 237)	36 (25·1 to 48·5)	1702 (65·2 to 3472·1)	107 (53·8 to 158·7)	82 (39·9 to 118·2)	944 (620·4 to 1257·6)
Rwanda	2 (0·3 to 3·4)	0* (0·3 to 0·7)	38 (1·4 to 80·9)	207 (103·9 to 309·1)	1 (0·7 to 2)	1 (0·7 to 1·4)
Sao Tome and Principe	0* (0 to 0·2)	0*	0* (0 to 0·9)	15 (7·4 to 21·9)	0* (0 to 0·1)	0*
Senegal	3 (0·6 to 6·3)	1 (0·6 to 1·3)	37 (1·4 to 77·3)	0* (0·1 to 0·3)	2 (1 to 2·9)	2 (1·2 to 2·5)
Seychelles	†	0* (0 to 0·1)	2 (0·1 to 3·2)	175 (88 to 261·1)	0* (0·2 to 0·4)	0*
Sierra Leone	3 (0·7 to 5·3)	2 (1·3 to 2·5)	90 (3·3 to 189·7)	1 (0·3 to 0·9)	4 (2·1 to 6·2)	0* (0·3 to 0·6)
South Africa	126 (26·9 to 241·4)	85 (59·8 to 114·1)	3185 (125·4 to 6311·6)	151 (76·5 to 224·4)	172 (84·7 to 246·2)	2749 (1818·2 to 3641·3)
South Sudan	No estimates in INT$**					
Togo	0* (0·1 to 1)	0* (0·1 to 0·2)	7 (0·3 to 15·2)	3 (1·5 to 4·6)	0* (0·2 to 0·6)	0* (0 to 0·1)
Uganda	2 (0·4 to 5·4)	0* (0·3 to 0·7)	56 (2 to 122·6)	14 (6·9 to 20·7)	1 (0·6 to 1·9)	3 (2·1 to 4·3)
United Republic of Tanzania	5 (0·7 to 12·3)	1 (0·5 to 1)	41 (1·5 to 89·6)	17 (8·5 to 25·4)	2 (0·9 to 2·7)	10 (6·3 to 13·1)
Zambia	†	2 (1·5 to 3)	136 (5·1 to 281·1)	59 (29·7 to 88)	5 (2·6 to 7·6)	14 (8·9 to 18·1)
Zimbabwe	†	2 (1·1 to 2·2)	53 (2 to 107·7)	28 (14·3 to 42·3)	3 (1·6 to 4·8)	4 (2·3 to 4·7)
**Region of the Americas**						
Antigua and Barbuda	†	0* (0·1 to 0·2)	2 (0·1 to 3·6)	1 (0·7 to 2·1)	0* (0·1 to 0·2)	0*
Argentina	193 (43·8 to 359·6)	107 (74·6 to 142)	2535 (100·7 to 4982·9)	1816 (919·4 to 2691·8)	190 (93·8 to 271)	604 (400·2 to 798·6)
Bahamas (The)	4 (0·8 to 7)	1 (0·6 to 1·1)	12 (0·5 to 23·7)	8 (4·2 to 12·3)	1 (0·5 to 1·4)	0* (0·1 to 0·1)
Barbados	3 (0·5 to 4·6)	1 (0·5 to 0·9)	7 (0·3 to 14·2)	4 (2·1 to 6·1)	1 (0·3 to 0·8)	0* (0 to 0·1)
Belize	0* (0 to 0·4)	0* (0·1 to 0·3)	3 (0·1 to 6·9)	1 (0·7 to 2·1)	0* (0·1 to 0·2)	0* (0 to 0)
Bolivia (Plurinational State of)	10 (1·8 to 18·4)	2 (1·4 to 2·7)	185 (7·2 to 370·2)	65 (32·8 to 96·3)	8 (4 to 11·8)	18 (11·8 to 23·8)
Brazil	726 (157·4 to 1139·3)	277 (194·6 to 368·1)	17203 (692 to 33379·2)	8452 (4284·7 to 12506·4)	1028 (508·9 to 1458·4)	65243 (43348·1 to 86023·5)
Canada	903 (194·7 to 1744)	124 (86·2 to 166·5)	1860 (71·5 to 3777·5)	1088 (548·5 to 1617·8)	137 (66·9 to 197·7)	872 (573·9 to 1161·4)
Chile	501 (83·3 to 1007·9)	27 (19 to 36·8)	900 (34·4 to 1837·5)	517 (260·5 to 769·2)	49 (24 to 71·1)	214 (140·5 to 284·9)
Colombia	156 (31·4 to 292·7)	87 (60·7 to 115·1)	1805 (72·1 to 3526·5)	1334 (675·6 to 1975·2)	134 (66·2 to 190·6)	2816 (1868·3 to 3719·1)
Costa Rica	10 (2 to 18·5)	6 (4·1 to 7·8)	186 (7·5 to 362·2)	96 (48·7 to 142·2)	8 (4·1 to 11·8)	30 (20·1 to 39·9)

Cuba						
Dominica	†	0* (0·1 to 0·1)	1 (0 to 1·8)	0*	0* (0 to 0·1)	1 (0·4 to 0·9)
Dominican Republic (The)	17 (3·8 to 32·5)	19 (13·6 to 25·9)	399 (15·7 to 788·5)	191 (96·5 to 283)	27 (13·3 to 38·7)	59 (38·8 to 77·6)
Ecuador	17 (3·1 to 33·3)	4 (2·6 to 5·1)	316 (12·1 to 645·3)	114 (57·5 to 169·7)	14 (7 to 20·8)	59 (38·7 to 78·4)
El Salvador	7 (1·2 to 13·4)	3 (2 to 3·8)	100 (3·9 to 200·1)	41 (20·5 to 60·4)	4 (2·1 to 6)	10 (6·3 to 12·7)
Grenada	†	0* (0·1 to 0·1)	1 (0 to 2·4)	1 (0·4 to 1·1)	0* (0 to 0·1)	0*
Guatemala	15 (2·8 to 28·8)	4 (3·1 to 6)	234 (9·2 to 464·5)	83 (41·8 to 122·7)	8 (3·8 to 11·2)	95 (62·7 to 125·7)
Guyana	1 (0·2 to 1·9)	1 (0·5 to 0·9)	18 (0·7 to 35·7)	7 (3·3 to 9·7)	9 (4·5 to 13·1)	0* (0·2 to 0·4)
Haiti	11 (1·9 to 22·2)	5 (3·2 to 6·1)	111 (4·4 to 222·5)	41 (20·9 to 61·5)	7 (3·6 to 10·6)	18 (11·9 to 23·9)
Honduras	7 (1·2 to 14·2)	2 (1·6 to 3·1)	87 (3·4 to 174·6)	40 (20·5 to 60·1)	3 (1·6 to 4·8)	10 (6·7 to 13·4)
Jamaica	6 (1·3 to 12)	3 (1·9 to 3·7)	36 (1·4 to 72·6)	24 (12·3 to 36·3)	3 (1·6 to 4·8)	4 (2·6 to 5·2)
Mexico	145 (30·3 to 276·5)	112 (78·2 to 150·9)	4097 (157·7 to 8316·7)	1600 (806·8 to 2379·5)	164 (80·1 to 236·5)	17711 (11653 to 23572·8)
Nicaragua	7 (1·4 to 14·2)	3 (1·8 to 3·5)	106 (4·1 to 212)	51 (25·6 to 75·3)	4 (2·2 to 6·4)	10 (6·8 to 13·6)
Panama	71 (12·2 to 137·4)	5 (3·4 to 6·6)	143 (5·6 to 285·2)	89 (45·1 to 132·5)	10 (4·8 to 13·9)	7 (4·9 to 9·9)
Paraguay	12 (2·7 to 23·5)	3 (2·1 to 4·1)	234 (9·2 to 463·9)	123 (62·4 to 183)	14 (7·1 to 20·5)	14 (9 to 18)
Peru	51 (9·2 to 99·7)	7 (5 to 9·6)	374 (14·6 to 747·9)	187 (94·3 to 277·1)	24 (11·8 to 34·6)	154 (101·7 to 204·4)
Saint Kitts and Nevis	†	0* (0·1 to 0·1)	2 (0·1 to 3·6)	0*	0* (0·1 to 0·2)	0*
Saint Lucia						
Saint Vicent and the Grenadines	†	0* (0·1 to 0·1)	1 (0 to 2·6)	1 (0·4 to 1·2)	0* (0·1 to 0·2)	0*
Suriname	5 (1·4 to 9·6)	12 (8·5 to 16·1)	239 (9·5 to 466·8)	100 (50·5 to 147·7)	14 (6·7 to 19·5)	3 (2 to 3·9)
Trinidad and Tobago	38 (6·6 to 74·4)	7 (4·6 to 8·8)	87 (3·4 to 172·6)	50 (25·3 to 74·3)	6 (2·8 to 8·1)	2 (1·4 to 2·8)
United States of America	6254 (1283·9 to 12043·7)	2358 (1649·9 to 3144·5)	61219 (2421·6 to 120784·1)	23436 (11856·9 to 34746·6)	3807 (1872·7 to 5428)	469832 (310997·8 to 621684·2)
Uruguay	52 (10·3 to 104·2)	6 (4·1 to 7·9)	118 (4·5 to 243)	86 (43·4 to 128·4)	12 (5·7 to 16·9)	4 (2·3 to 4·7)
Venezuela (Bolivarian Republic of)	No estimates in INT$**					
**Eastern Mediterranean Region**						
Afghanistan	27 (4·2 to 38·6)	18 (12·5 to 23·8)	508 (20·1 to 1004·2)	173 (87·6 to 256·9)	28 (13·6 to 39·4)	76 (50·4 to 100·8)
Bahrain	51 (10·5 to 103·2)	8 (5·9 to 11·2)	180 (7·1 to 356)	39 (19·8 to 57·9)	5 (2·4 to 6·9)	4 (2·6 to 5·2)
Djibouti	1 (0·1 to 1·6)	0* (0·1 to 0·1)	5 (0·2 to 9·6)	2 (0·8 to 2·4)	0* (0·1 to 0·3)	0*
Egypt	244 (44·4 to 511·3)	397 (276·4 to 532·3)	5160 (199·7 to 10416·3)	2469 (1245·7 to 3669·6)	466 (227·6 to 669·7)	2009 (1323·3 to 2671)
Iran (Islamic Republic of)	211 (39·6 to 412·1)	622 (433·4 to 832·3)	8635 (336 to 17331·4)	2652 (1338·9 to 3938·9)	433 (211·8 to 621·1)	5094 (3359·7 to 6765)
Iraq	50 (12·5 to 89·7)	53 (37 to 69·6)	927 (37·7 to 1777·9)	425 (215·9 to 628·3)	98 (48·6 to 138·2)	553 (368·1 to 726·8)

Jordan	5 (1 to 9·3)	5 (3·7 to 7·3)	107 (3·9 to 227·3)	27 (13·7 to 40·9)	7 (3·5 to 10·6)	20 (13·2 to 27·2)
Kuwait	182 (39·2 to 338·6)	43 (30·2 to 56)	892 (37·6 to 1656·6)	343 (174·6 to 504)	39 (19·6 to 54·3)	91 (61 to 118·7)
Lebanon	10 (2·1 to 19)	23 (15·7 to 30·1)	306 (12 to 609·4)	142 (71·6 to 210·4)	19 (9·2 to 26·9)	34 (22·4 to 45)
Libya	12 (3·1 to 22·1)	28 (19·4 to 37·1)	531 (20·8 to 1057·2)	183 (92·6 to 271·9)	31 (15·4 to 45)	124 (82·1 to 164·8)
Morocco	35 (7·1 to 69·3)	33 (22·9 to 44·4)	575 (22 to 1176·1)	230 (116·1 to 342·9)	37 (18·1 to 53·8)	185 (121·5 to 246·6)
Oman	72 (12·4 to 140·1)	10 (7·2 to 13·8)	279 (10·9 to 561·3)	87 (43·9 to 129·1)	9 (4·2 to 12·2)	8 (5·2 to 10·5)
Pakistan	328 (55·2 to 692)	87 (60·5 to 116)	1858 (72·4 to 3724·4)	1377 (695·2 to 2044·6)	113 (55·3 to 162·1)	4808 (3171·9 to 6383·4)
Qatar	14 (3 to 26·9)	11 (7·4 to 14·1)	403 (15·8 to 801)	93 (47 to 137·9)	8 (4 to 11·7)	24 (16·2 to 32·4)
Saudi Arabia	695 (148·4 to 1288·9)	281 (197·9 to 371·8)	7362 (300·4 to 14087·1)	2547 (1293·4 to 3762·1)	382 (190·3 to 539·9)	3066 (2043·3 to 4029·8)
Somalia	5 (1·1 to 9·2)	10 (7·3 to 14)	722 (28·5 to 1427·6)	271 (137·1 to 401·9)	36 (17·5 to 50·8)	57 (37·6 to 75·2)
Sudan	29 (6·7 to 56·3)	25 (17·7 to 33·8)	548 (21·6 to 1082·7)	194 (98·4 to 288·4)	171 (84·2 to 244·4)	125 (82·9 to 165·8)
Syrian Arab Republic	No estimates in INT$**					
Tunisia	23 (4·4 to 48·3)	45 (31·3 to 60·3)	738 (28·5 to 1492)	161 (81·1 to 238·9)	44 (21·3 to 62·6)	73 (47·9 to 96·7)
United Arab Emirates	50 (11·3 to 92·1)	34 (23·9 to 45·5)	907 (36 to 1782·6)	574 (290·8 to 851·4)	68 (33·6 to 97·1)	201 (133·4 to 266·2)
Yemen	No estimates in INT$**					
**European Region**						
Albania	8 (0·9 to 14)	3 (2 to 3·9)	33 (1·3 to 66·3)	49 (24·8 to 73·2)	6 (3·1 to 9·2)	1 (0·9 to 1·9)
Andorra	No estimates in INT$**					
Armenia	9 (1·5 to 15·5)	9 (6·4 to 12·4)	68 (2·6 to 140·4)	59 (29·8 to 88·1)	7 (3·2 to 9·5)	2 (1·4 to 2·9)
Austria	440 (80·1 to 756·9)	52 (36·4 to 70·2)	689 (26·6 to 1393·6)	543 (273·8 to 807)	70 (34·3 to 101·1)	58 (38 to 76·8)
Azerbaijan	33 (4·3 to 60·6)	36 (24·9 to 48·1)	288 (11·1 to 583·1)	182 (92 to 271·2)	32 (15·6 to 46·1)	15 (10·1 to 20·4)
Belarus	35 (5·7 to 63·4)	39 (26·7 to 52·5)	362 (13·4 to 767·3)	220 (110·7 to 329·3)	39 (18·7 to 56·8)	10 (6·2 to 12·8)
Belgium	720 (124 to 1166·8)	43 (30 to 57·4)	996 (39 to 1985·6)	652 (329·3 to 967·4)	56 (27·6 to 80·6)	76 (50·4 to 101·3)
Bosnia and Herzegovina	13 (2·2 to 21·7)	8 (5·2 to 10·2)	83 (3·2 to 169·5)	109 (54·8 to 162)	18 (8·7 to 25·9)	8 (5·2 to 10·6)
Bulgaria	35 (5·7 to 55·7)	39 (27·6 to 52·6)	156 (6·2 to 309·6)	457 (231 to 677·4)	100 (49·2 to 143·1)	47 (31·3 to 62·6)
Croatia	101 (16·8 to 168·9)	13 (9·1 to 17·6)	122 (4·7 to 246·9)	218 (110·1 to 324·4)	24 (12 to 35·2)	6 (3·8 to 7·6)
Cyprus	32 (6 to 38·9)	2 (1·6 to 3)	49 (1·9 to 94·8)	42 (21·4 to 62·6)	1 (0·7 to 2)	2 (1 to 2)
Czechia	386 (71·8 to 662·4)	32 (22 to 42·4)	409 (15·8 to 824·5)	719 (362·8 to 1068·6)	64 (31·3 to 92·2)	12 (7·9 to 15·9)
Denmark	219 (39·4 to 391·1)	13 (9 to 17·3)	382 (14·7 to 776)	258 (130·1 to 383·9)	11 (5·5 to 16·1)	21 (13·5 to 27·3)
Estonia	32 (5·7 to 56·1)	5 (3·8 to 7·2)	49 (1·9 to 97·7)	64 (32·2 to 94·7)	9 (4·5 to 13·2)	0 (0·3 to 0·7)
Finland	68 (12·5 to 221)	16 (11 to 21·5)	174 (6·5 to 364·8)	117 (58·9 to 174·9)	27 (13 to 39·3)	16 (10·4 to 21·4)

France	3003 (577·1 to 5350·4)	178 (123·8 to 238·9)	4329 (166·8 to 8778)	3491 (1760·8 to 5191·8)	184 (89·5 to 264·1)	2024 (1332·2 to 2693·7)
Georgia	15 (2·4 to 40)	10 (7 to 13·7)	83 (3·1 to 173·8)	76 (38·4 to 113·9)	12 (5·7 to 17·3)	3 (2·2 to 4·5)
Germany	6290 (1017·3 to 7947·9)	680 (476·4 to 905·7)	8788 (349·5 to 17246·6)	7647 (3871·4 to 11330·9)	1049 (517 to 1493·1)	17044 (11295·6 to 22527·9)
Greece	296 (44·5 to 390·3)	55 (38·7 to 74)	543 (21·3 to 1076·9)	398 (201·1 to 590·1)	46 (22·7 to 66)	46 (30·7 to 61·6)
Hungary	312 (53·8 to 428·5)	50 (35·2 to 67·1)	477 (18·8 to 943·9)	706 (357·1 to 1047·3)	71 (34·9 to 101·3)	67 (44 to 88·1)
Iceland	11 (2 to 19·9)	1 (0·6 to 1·1)	13 (0·5 to 25·7)	11 (5·7 to 16·7)	1 (0·5 to 1·4)	0* (0·1 to 0·1)
Ireland	190 (35·2 to 317·1)	13 (8·9 to 17)	324 (12·6 to 651·9)	211 (106·7 to 314)	13 (6·2 to 18·2)	10 (6·7 to 13·5)
Israel	238 (39·2 to 413·7)	8 (5·5 to 10·7)	267 (10·3 to 541·7)	107 (54 to 159·3)	12 (5·7 to 17)	32 (21·1 to 42·7)
Italy	3304 (514·3 to 4108·3)	381 (266·7 to 507·5)	4393 (174·3 to 8637·2)	3929 (1988·7 to 5823·3)	457 (225·1 to 651)	4166 (2759·6 to 5508·5)
Kazakhstan	64 (10 to 138·6)	47 (32·4 to 62·6)	577 (22·1 to 1175·5)	392 (197·5 to 583)	65 (31·5 to 93·2)	79 (52·2 to 105·8)
Kyrgyzstan	4 (0·5 to 18·9)	2 (1·7 to 3·4)	32 (1·2 to 68·5)	19 (9·5 to 28·2)	2 (1·1 to 3·4)	1 (0·4 to 0·9)
Latvia	46 (7·4 to 84·1)	4 (3 to 5·8)	61 (2·3 to 123·1)	81 (41·1 to 121·2)	6 (3 to 8·9)	1 (0·5 to 1)
Lithuania	79 (12·6 to 167·2)	9 (6·3 to 12·2)	86 (3·3 to 176)	137 (69·2 to 204·4)	12 (6 to 17·8)	1 (0·9 to 1·9)
Luxembourg	27 (4·8 to 50·4)	2 (1·2 to 2·3)	53 (2 to 106·9)	28 (13·9 to 41)	2 (0·9 to 2·8)	0* (0·3 to 0·6)
Malta	27 (4·5 to 33·8)	1 (0·8 to 1·6)	33 (1·3 to 65·1)	28 (14·4 to 42·2)	2 (0·8 to 2·3)	0* (0·2 to 0·3)
Monaco	No estimates in INT$**					
Montenegro	2 (0·4 to 4·1)	3 (2·3 to 4·4)	40 (1·5 to 80·5)	0*	6 (2·8 to 8·2)	16 (10·3 to 20·9)
Netherlands	1022 (168·7 to 1688·3)	128 (88·9 to 172)	1313 (50·3 to 2676·8)	896 (451·5 to 1332·9)	83 (40·3 to 119·3)	174 (114·7 to 232·4)
North Macedonia	79 (11·9 to 134·1)	4 (2·9 to 5·7)	52 (2 to 104·9)	0*	14 (6·7 to 19·9)	2 (1·5 to 3·1)
Norway	239 (43·5 to 390·9)	20 (13·7 to 26·3)	373 (14·4 to 750·9)	271 (136·8 to 402·9)	31 (15 to 44·2)	22 (14·4 to 29)
Poland	1084 (170·9 to 1798·6)	83 (57·8 to 111)	1405 (54·6 to 2825·6)	2088 (1054·1 to 3102·3)	131 (64 to 188)	562 (370·7 to 747)
Portugal	356 (60·9 to 451·6)	18 (12·9 to 24·5)	2906 (115·9 to 5685·8)	758 (383·8 to 1122·6)	40 (19·8 to 57·1)	152 (100·9 to 201)
Republic of Moldova	7 (1·1 to 33·5)	4 (2·7 to 5·2)	36 (1·3 to 76·7)	28 (14·1 to 42·2)	4 (1·8 to 5·6)	1 (0·5 to 1·1)
Romania	110 (19·2 to 161·7)	37 (25·8 to 49·4)	519 (20·3 to 1036)	1111 (561·2 to 1649·1)	172 (84·4 to 246·6)	106 (69·9 to 140·4)
Russian Federation	420 (84·5 to 1595·4)	549 (379·1 to 742·1)	4525 (169 to 9492·5)	4372 (2197·4 to 6525·6)	767 (369·6 to 1114·2)	4267 (2789 to 5716·3)
San Marino	No estimates in INT$**					
Serbia	54 (9·3 to 69·4)	50 (35·3 to 67·3)	412 (16·3 to 814·4)	497 (251·5 to 737·4)	85 (42 to 121·9)	91 (60·1 to 120·2)
Slovakia	176 (30·8 to 207·1)	20 (13·8 to 26·5)	144 (5·6 to 287·7)	246 (124·2 to 364·9)	30 (14·6 to 42·6)	11 (7·1 to 14·3)
Slovenia	71 (12·6 to 118·2)	4 (2·8 to 5·4)	98 (3·8 to 196·4)	136 (68·7 to 202·2)	7 (3·3 to 9·7)	4 (2·5 to 5)
Spain	1148 (191·7 to 1674·1)	138 (95·9 to 185·5)	2256 (86·4 to 4605·4)	1689 (851·2 to 2513·6)	154 (75 to 222·4)	2170 (1426·2 to 2891·8)
Sweden	211 (36·7 to 463·7)	17 (11·7 to 22·7)	605 (23 to 1247·9)	387 (194·7 to 576·1)	40 (19·3 to 57·5)	57 (37·6 to 76·5)

Switzerland	363 (47·3 to 776·4)	26 (18·1 to 35·1)	579 (22 to 1192·7)	321 (161·9 to 478·8)	30 (14·4 to 42·8)	52 (34·4 to 70)
Tajikistan	8 (1 to 16·9)	6 (4·3 to 8·3)	70 (2·7 to 141·6)	57 (28·9 to 85·2)	6 (2·9 to 8·4)	4 (2·6 to 5·2)
Turkey	494 (80 to 821·6)	342 (238·2 to 458·9)	7214 (278·9 to 14576·2)	3025 (1526·1 to 4496·6)	529 (258·1 to 760·1)	8480 (5584·4 to 11275·4)
Turkmenistan	No estimates in INT$**					
Ukraine	184 (26·7 to 523·1)	211 (146·3 to 285·4)	1700 (63·9 to 3541·6)	1197 (601·8 to 1784·5)	179 (86·5 to 259·5)	266 (173·8 to 355·2)
United Kingdom of Great Britain and Northern Ireland (the)	9300 (1837·2 to 14410·1)	233 (162·6 to 311·3)	6354 (248·8 to 12664·7)	2706 (1367·3 to 4016·3)	264 (129·3 to 377·2)	5721 (3778·7 to 7586·5)
Uzbekistan	29 (4·2 to 86·1)	31 (21·5 to 41·9)	319 (12 to 665·4)	244 (122·7 to 364·1)	34 (16·2 to 48·6)	42 (27·3 to 55·8)
**South-East Asia Region**						
Bangladesh	216 (19 to 528·4)	26 (18·3 to 35·4)	660 (25·3 to 1342·7)	274 (138·2 to 407·8)	33 (16 to 47·5)	571 (375·6 to 760·7)
Bhutan	0* (0 to 0·4)	0* (0·5 to 0·5)	7 (7·2 to 7·2)	16 (15·8 to 15·8)	0 (0·2 to 0·5)	0 (0 to 0)
Democratic People's Republic of Korea	No estimates in INT$**					
India	2369 (374·6 to 4925·3)	920 (642·1 to 1232)	15455 (602·5 to 30955·2)	9846 (4972·9 to 14623·4)	866 (423·8 to 1240·8)	89776 (59232·7 to 119163·7)
Indonesia	168 (41·7 to 223·7)	30 (21 to 40·7)	1330 (50·4 to 2746·1)	1473 (741·4 to 2194·7)	365 (176·5 to 527)	5987 (3925·1 to 7995·4)
Maldives	0* (0·1 to 0·8)	0* (0·1 to 0·3)	9 (0·4 to 18·6)	7 (3·8 to 11·1)	1 (0·3 to 0·9)	0 (0 to 0·1)
Myanmar	21 (2·6 to 47·9)	5 (3·2 to 6·3)	117 (4·3 to 250·4)	144 (72 to 214·7)	16 (7·8 to 23·9)	66 (42·9 to 88·7)
Nepal	5 (0·7 to 10·3)	5 (3·5 to 6·9)	149 (5·5 to 316·5)	58 (29 to 86·2)	4 (1·7 to 5·1)	9 (5·6 to 11·6)
Sri Lanka	53 (7·9 to 114·3)	15 (10·1 to 19·5)	314 (12·1 to 637·7)	294 (148·3 to 437·2)	41 (20 to 59·2)	92 (60·7 to 122·7)
Thailand	103 (22·2 to 198·2)	29 (20·3 to 39·3)	777 (29·6 to 1595·9)	781 (393·5 to 1163·6)	105 (51·1 to 152)	53 (34·5 to 70·1)
Timor-Leste	0 (0 to 0·3)	0* (0·1 to 0·2)	4 (0·2 to 8·6)	3 (1·6 to 4·8)	1 (0·3 to 1)	0 (0 to 0·1)
**Western Pacific Region**						
Australia	708 (155·5 to 1216)	126 (87·9 to 169·3)	1770 (68·4 to 3578·3)	1065 (537·3 to 1583·2)	76 (37·1 to 109·2)	355 (233·4 to 471·5)
Brunei Darussalam	4 (0·7 to 7·4)	0* (0·2 to 0·5)	9 (0·3 to 17·8)	14 (7 to 20·6)	1 (0·6 to 1·7)	0* (0·1 to 0·2)
Cambodia	5 (0·8 to 9·1)	1 (0·6 to 1·1)	30 (1·1 to 65·1)	35 (17·6 to 52·6)	4 (1·9 to 5·9)	4 (2·9 to 6)
China	1814 (249·3 to 3752·6)	399 (274·9 to 540·1)	7814 (289·7 to 16540·3)	8778 (4407·5 to 13112)	1709 (820·7 to 2487·4)	302021 (197053·4 to 405268·5)
Cook Islands	No estimates in INT$**					
Fiji	0* (0·1 to 0·9)	0* (0·1 to 0·2)	3 (0·1 to 6·6)	4 (1·8 to 5·2)	0* (0·2 to 0·6)	0* (0·2 to 0·4)
Japan	9298 (1235·3 to 17488)	436 (304·1 to 582·6)	6 (0·2 to 11·2)	10 (5 to 14·8)	1304 (638·9 to 1865·7)	7660 (5058·2 to 10159·8)
Kiribati	†	0* (0·3 to 0·5)	14 (0·6 to 27·5)	16 (8·2 to 24)	3 (1·4 to 4·1)	0* (0·1 to 0·2)
Lao People's Democratic Republic	1 (0·2 to 2·5)	1 (1 to 1·9)	47 (1·8 to 99·3)	42 (21·1 to 62·7)	5 (2·4 to 7·3)	3 (1·7 to 3·5)
Malaysia	39 (8·3 to 66·2)	20 (14·3 to 27·3)	688 (27·1 to 1361·5)	640 (323·9 to 949·8)	68 (33·3 to 96·8)	620 (410·2 to 821·1)
Marshall Islands	†	0*	0* (0 to 0·7)	1 (0·4 to 1·1)	0* (0 to 0·1)	0* (0 to 0)

Micronesia (Federated States of)	2 (0·3 to 2·8)	0*	1 (0 to 1·7)	1 (0·5 to 1·6)	0* (0·1 to 0·2)	3 (2·1 to 4·2)
Mongolia	4 (0·2 to 10)	3 (2 to 3·9)	49 (1·8 to 102·1)	38 (19·3 to 57·3)	4 (2·1 to 6·4)	0* (0·3 to 0·6)
Nauru	†	0*	0* (0 to 0·4)	0* (0·2 to 0·6)	0* (0 to 0)	0* (0 to 0)
New Zealand	122 (27·3 to 209·9)	19 (13·2 to 25·1)	252 (10 to 494·2)	231 (117·1 to 342·7)	15 (7·3 to 21·2)	9 (6·3 to 12·5)
Niue	No estimates in INT$**					
Palau	†	0*	1 (0 to 1)	0*	0* (0 to 0·1)	0*
Papua New Guinea	2 (0·4 to 4·9)	0* (0·1 to 0·2)	5 (0·2 to 10·6)	4 (2·2 to 6·6)	1 (0·3 to 1)	3 (1·6 to 3·4)
Philippines	151 (36·5 to 261)	40 (27·7 to 52·8)	866 (34·2 to 1709·8)	924 (467·4 to 1369·9)	125 (61·5 to 178·3)	956 (632·5 to 1264·7)
Republic of Korea	533 (59·6 to 1031·1)	74 (51·7 to 99)	1854 (72·5 to 3700·2)	0*	279 (136·9 to 399·8)	1713 (1131·3 to 2272·4)
Samoa	0* (0 to 0·2)	0*	0* (0 to 0·7)	0* (0·2 to 0·6)	0* (0 to 0·1)	0*
Singapore	121 (25·1 to 213·6)	17 (12 to 23)	307 (12 to 610·6)	364 (184·1 to 540·7)	37 (18·3 to 53·5)	40 (26·3 to 52·7)
Solomon Islands	0* (0 to 0·1)	0*	0* (0 to 0·8)	0* (0·2 to 0·5)	0* (0 to 0·1)	0*
Tonga	†	0*	0* (0 to 0·4)	0* (0·1 to 0·3)	0*	0*
Tuvalu	†	0*	0* (0 to 0·2)	0* (0·1 to 0·2)	0*	0*
Vanuatu	0*	0*	0* (0 to 0·2)	0* (0 to 0·1)	0*	0*
Viet Nam	122 (21·3 to 233·4)	26 (17·9 to 34·7)	581 (22·2 to 1190·5)	988 (497·9 to 1471·3)	149 (72·4 to 215·2)	806 (529·2 to 1074·6)

*Not zero costs, please see Table S8, for more details

** No estimates in INT$ (no available conversion rates for these countries), but available estimates in US$· Please, see Table S8

† No cost estimates

Table S10. Direct healthcare costs attributable to physical inactivity, by health outcomes, WHO regions and World Bank income classification (in US$), 2020-2030

	Breast cancer	Colorectal cancer	Endometrial cancer	Gastric cancer	Oseophageal cancer
African Region (AFR)	0.02 (0.01 to 0.04)	0.02 (0.01 to 0.04)	0.02 (-0.01 to 0.05)	0.05 (-0.01 to 0.11)	0.05 (-0.03 to 0.15)
Region of the Americas (AMR)	0.04 (0.01 to 0.06)	0.04 (0.01 to 0.06)	0.04 (-0.02 to 0.09)	0.09 (-0.03 to 0.19)	0.09 (-0.06 to 0.25)
Eastern Mediterranean Region (EMR)	0.04 (0.01 to 0.06)	0.04 (0.01 to 0.07)	0.04 (-0.02 to 0.09)	0.1 (-0.03 to 0.21)	0.1 (-0.06 to 0.27)
European Region (EUR)	0.03 (0.01 to 0.05)	0.03 (0.01 to 0.05)	0.03 (-0.01 to 0.07)	0.07 (-0.02 to 0.17)	0.08 (-0.05 to 0.22)
South-East Asia Region (SEAR)	0.03 (0.01 to 0.05)	0.02 (0.01 to 0.03)	0.03 (-0.02 to 0.08)	0.06 (-0.02 to 0.13)	0.06 (-0.03 to 0.18)
Western Pacific Region (WPR)	0.02 (0.01 to 0.03)	0.03 (0.01 to 0.05)	0.02 (-0.01 to 0.04)	0.08 (-0.02 to 0.17)	0.08 (-0.05 to 0.23)
Low-income	0.02 (0.01 to 0.03)	0.02 (0 to 0.03)	0.02 (-0.01 to 0.04)	0.04 (-0.01 to 0.1)	0.04 (-0.02 to 0.13)
Lower-middle-income	0.03 (0.01 to 0.05)	0.03 (0.01 to 0.05)	0.03 (-0.02 to 0.08)	0.07 (-0.02 to 0.17)	0.08 (-0.05 to 0.22)
Upper-middle-income	0.02 (0.01 to 0.03)	0.02 (0.01 to 0.04)	0.02 (-0.01 to 0.05)	0.06 (-0.02 to 0.13)	0.06 (-0.03 to 0.18)
High-income	0.04 (0.01 to 0.06)	0.04 (0.01 to 0.06)	0.04 (-0.02 to 0.08)	0.09 (-0.03 to 0.2)	0.09 (-0.06 to 0.26)

(continued from previous Table)

	Renal cancer	Bladder cancer	Stroke	Coronary Heart Disease	Type 2 diabetes
African Region (AFR)	0.05 (0.01 to 0.09)	0.01 (-0.01 to 0.04)	0.03 (0.02 to 0.05)	0.03 (0.02 to 0.05)	0.03 (0.02 to 0.04)
Region of the Americas (AMR)	0.09 (0.02 to 0.16)	0.03 (-0.03 to 0.08)	0.06 (0.03 to 0.09)	0.06 (0.04 to 0.08)	0.06 (0.04 to 0.07)
Eastern Mediterranean Region (EMR)	0.1 (0.02 to 0.18)	0.03 (-0.03 to 0.09)	0.07 (0.03 to 0.1)	0.07 (0.05 to 0.09)	0.06 (0.04 to 0.08)
European Region (EUR)	0.08 (0.02 to 0.14)	0.02 (-0.02 to 0.07)	0.05 (0.03 to 0.08)	0.05 (0.04 to 0.07)	0.05 (0.03 to 0.06)
South-East Asia Region (SEAR)	0.06 (0.01 to 0.11)	0.02 (-0.02 to 0.05)	0.04 (0.02 to 0.06)	0.04 (0.03 to 0.05)	0.04 (0.02 to 0.05)
Western Pacific Region (WPR)	0.08 (0.02 to 0.14)	0.02 (-0.02 to 0.07)	0.05 (0.03 to 0.08)	0.05 (0.04 to 0.07)	0.05 (0.03 to 0.07)
Low-income	0.04 (0.01 to 0.08)	0.01 (-0.01 to 0.04)	0.03 (0.01 to 0.04)	0.03 (0.02 to 0.04)	0.03 (0.02 to 0.04)
Lower-middle-income	0.08 (0.02 to 0.14)	0.02 (-0.02 to 0.07)	0.05 (0.03 to 0.08)	0.05 (0.04 to 0.07)	0.05 (0.03 to 0.06)
Upper-middle-income	0.06 (0.01 to 0.11)	0.02 (-0.02 to 0.05)	0.04 (0.02 to 0.06)	0.04 (0.03 to 0.06)	0.04 (0.02 to 0.05)
High-income	0.09 (0.02 to 0.17)	0.03 (-0.03 to 0.08)	0.07 (0.03 to 0.09)	0.07 (0.05 to 0.09)	0.06 (0.04 to 0.08)

(continued from previous Table)

	Depression	Hypertension	Dementia
African Region (AFR)	0.05 (0 to 0.1)	0.01 (0.01 to 0.02)	0.08 (0.04 to 0.13)

Region of the Americas (AMR)	0.09 (0 to 0.18)	0.02 (0.01 to 0.03)	0.14 (0.07 to 0.22)
Eastern Mediterranean Region (EMR)	0.1 (0 to 0.2)	0.02 (0.01 to 0.03)	0.12 (0.07 to 0.2)
European Region (EUR)	0.08 (0 to 0.15)	0.02 (0.01 to 0.03)	0.11 (0.06 to 0.17)
South-East Asia Region (SEAR)	0.06 (0 to 0.12)	0.01 (0.01 to 0.02)	0.11 (0.06 to 0.18)
Western Pacific Region (WPR)	0.08 (0 to 0.16)	0.02 (0.01 to 0.03)	0.07 (0.04 to 0.12)
Low-income	0.04 (0 to 0.09)	0.01 (0 to 0)	0.06 (0.03 to 0.1)
Lower-middle-income	0.08 (0 to 0.16)	0.02 (0.01 to 0.03)	0.11 (0.06 to 0.17)
Upper-middle-income	0.06 (0 to 0.12)	0.01 (0.01 to 0.02)	0.08 (0.04 to 0.14)
High-income	0.09 (0 to 0.19)	0.02 (0.01 to 0.03)	0.13 (0.07 to 0.2)

Table S11. Sensitivity analysis: varying costs of incident cases by -30% and -50%, by WHO Regions and World Bank income classification, in US$, 2020-2030

	Cancers	Coronary Heart Diseases	Dementia	Depression	Hypertension	Stroke	Type 2 Diabetes
**WHO Regions**	Varying costs in -30%						
African Region	101296419	38110049	105091164	1780521012	844258142	84806034	310750762
Region of the Americas	3396574801	502391184	19938850239	13470478512	7542898928	937463778	11655740606
Eastern Mediterranean Region	2055049410	805926598	254254000	12467606437	6353254946	903987320	1134353119
European Region	10319628119	1598213174	9490601206	31172577599	17393085264	2130541866	3494534709
South-East Asia Region	372824859	266168829	2383278747	4261022174	3505892742	379618555	370195397
Western Pacific region	8259380610	808927840	10308031028	13042979150	12423918354	2955375454	4315298412
**World Bank classification**							
Low	35570294	8735467	36534143	419414047	198260483	20679916	32577132
Lower-middle	615382836	426090796	1641564394	7598323663	4972297262	604569313	1037873343
Upper-middle	2450578456	742795246	7544940840	12846260174	9182009978	1986257959	4420165853
High	28572419260	2135254933	37669860166	45869840487	31303406187	3400198088	13293652917
**Total Global**	**31673950846**	**3312876442**	**46892899542**	**66733838370**	**45655973910**	**6011705275**	**18784269246**
**WHO Regions**	Varying costs in -50%						
African Region	72354585	27221464	75065117	1271809478	603041530	60575738	221962891
Region of the Americas	2426124858	358850845	14242035885	9621836605	5387784949	669616984	8325533005
Eastern Mediterranean Region	1467892435	575661856	181610000	8905494477	4538039247	645705229	810257795
European Region	7371162942	1141580839	6779000861	17069381771	12423632331	1521815619	2496098843
South-East Asia Region	266303471	190120592	1702341962	3043608220	2504209101	271156111	264433367
Western Pacific region	5899557579	577805600	7362879306	9316477815	8874227395	2110982467	3082338009
**World Bank classification**							
Low	25407353	6239620	26095816	299583525	141614631	14771368	23269380
Lower-middle	439559169	304350568	1172545995	5427411409	3551640902	431835224	741338102
Upper-middle	1750413183	530568033	5389243457	9046890094	6558578555	1418755685	3157261324
High	20408870900	1525182095	26907042976	27138421036	22359575848	2428712920	9495466369
**Total Global**	**22624250605**	**2366340316**	**33494928245**	**41912306063**	**32611409936**	**4294075197**	**13417335175**

Appendix 1. WHO Regions and countries

WHO regions (194 countries)

African Region (AFRO): 47 countries

Algeria, Angola, Benin, Botswana, Burkina Faso, Burundi, Cabo Verde, Cameroon, Central African Republic, Chad, Comoros (the), Congo, Côte d'Ivoire, Democratic Republic of the Congo, Equatorial Guinea, Eritrea, Eswatini, Ethiopia, Gabon, Ghana, Guinea, Guinea-Bissau, Kenya, Lesotho, Liberia, Madagascar, Malawi, Mali, Mauritania, Mauritius, Mozambique, Namibia, Niger, Nigeria, Rwanda, Sao Tome and Principe, Senegal, Seychelles, Sierra Leone, South Africa, South Sudan, the Gambia, Togo, Uganda, United Republic of Tanzania, Zambia, and Zimbabwe.

Region of the Americas (AMRO): 35 countries

Antigua and Barbuda, Argentina, Bahamas (the), Barbados, Belize, Bolivia (Plurinational State of), Brazil, Canada, Chile, Colombia, Costa Rica, Cuba, Dominica, Dominican Republic (the), Ecuador, El Salvador, Grenada, Guatemala, Guyana, Haiti, Honduras, Jamaica, Mexico, Nicaragua, Panama, Paraguay, Peru, Saint Kitts and Nevis, Saint Lucia, Saint Vincent and the Grenadines, Suriname, Trinidad and Tobago, United States of America, Uruguay, and Venezuela (Bolivarian Republic of).

South-East Asia Region (SEARO): 11 countries

Bangladesh, Bhutan, Democratic People's Republic of Korea, India, Indonesia, Maldives, Myanmar, Nepal, Sri Lanka, Thailand, and Timor-Leste.

European Region (EURO): 53 countries

Albania, Andorra, Armenia, Austria, Azerbaijan, Belarus, Belgium, Bosnia and Herzegovina, Bulgaria, Croatia, Cyprus, Czechia, Denmark, Estonia, Finland, France, Georgia, Germany, Greece, Hungary, Iceland, Ireland, Israel, Italy, Kazakhstan, Kyrgyzstan, Latvia, Lithuania, Luxembourg, Malta, Monaco, Montenegro, Netherlands, North Macedonia, Norway, Poland, Portugal, Republic of Moldova, Romania, Russian Federation, San Marino, Serbia, Slovakia, Slovenia, Spain, Sweden, Switzerland, Tajikistan, Turkey, Turkmenistan, Ukraine, United Kingdom of Great Britain and Northern Ireland (the), and Uzbekistan.

Eastern Mediterranean Region (EMRO): 21 countries

Afghanistan, Bahrain, Djibouti, Egypt, Iran (Islamic Republic of), Iraq, Jordan, Kuwait, Lebanon, Libya, Morocco, Oman, Pakistan, Qatar, Saudi Arabia, Somalia, Sudan, Syrian Arab Republic, Tunisia, United Arab Emirates, and Yemen.

Western Pacific Region (WPRO): 27 countries

Australia, Brunei Darussalam, Cambodia, China, Cook Islands, Fiji, Japan, Kiribati, Lao People's Democratic Republic, Malaysia, Marshall Islands, Micronesia (Federated States of), Mongolia, Nauru, New Zealand, Niue, Palau, Papua New Guinea, Philippines, Republic of Korea, Samoa, Singapore, Solomon Islands, Tonga, Tuvalu, Vanuatu, and Viet Nam.

Appendix 2. Estimating the costs for diabetes complications: the approach used to avoid double counting

IDF's direct healthcare costs estimates for diabetes account for complications of diabetes related to coronary heart diseases and stroke.· To subtract these costs from the total costs by county, WHO Regions and World Bank income levels, we estimated the number of incident cases due to (1) patients with diabetes and coronary heart disease, (2) diabetes and stroke and (3) diabetes and coronary heart disease and stroke, and subtracted the costs associated to these comorbidities from the total costs· The illustrate the approach used, we show here the example of SEARO:

The risk of diabetes (incidence proportion) in the total population (R_{dt} = 0·0010726) for both sexes was estimated by dividing the total number of new cases of diabetes in SEARO (1,414,955) by the total population of SEARO (1,319,203,305)· The same calculation was used for the risk of stroke (Rst) and coronary heart disease (Rchdt) in the total population: 0·0017706 (Rst) and 0·0043688 (Rchdt), respectively·

The risk of stroke among those without diabetes ($R_{s/nd}$) was derived as:

1) the risk in the total population (R_{st}) was considered as the weighted average of the risk in diabetics and non-diabetics:
    a. (risk in diabetics x proportion with diabetics) +
    b. (risk in non-diabetics ($R_{s/nd}$) x proportion without diabetics);
2) the proportion of individuals with diabetes was the R_{dt}
3) the proportion of non-diabetics = (1-R_{dt})
4) the risk among diabetics was the $R_{s/nd}$ multiplied by the relative risk for stroke by comparing diabetics to non-diabetics (RR_s)
5) therefore, $R_{st} = (RR_s \text{ x } R_{dt} \text{ x } R_{s/nd}) + ((1 - R_{dt}) \text{ x } R_{s/nd})$
6) rearranging the formula, $R_{s/nd} = \frac{R_{st}}{(RR_s \text{ x } R_{dt}) + (1 - R_{dt})}$
7) if R_{dt} = 0·0010726, R_{st} = 0·0017706, and RR_s = 2·0, as in Sarwar and colleagues (2010)[36], the $R_{s/nd}$ = 0·0017687, and Rs/d = 0·0017687 x 2·0 = 0·0035373
8) We then repeated the same calculation for coronary heart disease (and all WHO Regions and World Bank income level), and the results are presented in the table below, for the region SEARO, as an example·

**Parameter**		**Numerical value**
Total population	Pop	1,319,203,305
New cases of diabetes		1,414,956
Risk of diabetes/population	R_{dt}	0·0010726
1-R_{dt}	R_{ndt}	0·9989274
New cases of ischemic stroke		2,335,732
Risk of stroke in the total population	R_{st}	0·0017706
1-R_{st}	R_{nst}	0·9982294
Relative risk for stroke among diabetics	RR_s	2·00
Risk of stroke among diabetics	$R_{s/d}$	0·0035373
Risk of stroke among non-diabetics	$R_{s/nd}$	0·0017687
New cases of coronary heart diseases (CHD)		5,763,323
Risk of CHD in the total population	R_{chdt}	0·0043688
1-R_{chdt}		0·9956312
Relative risk of CHD among diabetics	RR_{chd}	2·00
R_{chd} given diabetes	$R_{chd/d}$	0·0087282

R_{chd} without diabetes	$R_{chd/nd}$	0·0043641

Assuming there is no interaction, the individuals with diabetes would be divided in four groups, as below·

Subgroups of diabetics	Stroke	CHD	Formula
1) Diabetics without chd or stroke	No	No	$(R_{dt}) \times (1-R_{chd/d}) \times (1-R_{s/d})$
2) Diabetics with stroke	Yes	No	$(R_{dt}) \times R_{s/d} \times (1-R_{chd/d})$
3) Diabetics with chd	No	Yes	$(R_{dt}) \times (1-R_{s/d}) \times (R_{chd/d})$
4) Diabetics with chd and stroke	Yes	Yes	$(R_{dt}) \times R_{s/d} \times R_{chd/d}$

The number of individuals with interacting diseases by WHO Region and World Bank income classification would then be: ·

	WHO Regions						World Bank income Group				
**Reference**	**SEAR**	**EMR**	**AMR**	**EUR**	**WPR**	**AFR**	**Low**	**Lower-middle**	**Upper-middle**	**High**	**TOTAL Global**
Diabetics with stroke	347	40	46	440	1070	53	26	920	782	802	**2530**
Diabetics with coronary heart disease	855	130	85	1188	1014	79	37	2241	1024	1779	**5081**
Diabetics with stroke and coronary heart disease	0·12	0·03	0·02	0·31	0·37	0·01	0	0·45	0·25	0·45	**1**
**TOTAL**	**1202**	**170**	**131**	**1628**	**2084**	**132**	**63**	**3162**	**1806**	**2582**	**7612**

The total cost for individuals with interacting diseases by WHO Region and World Bank income classification would be:

	WHO Regions						World Bank income Group				
**Reference**	**SEAR**	**EMR**	**AMR**	**EUR**	**WPR**	**AFR**	**Low-income**	**Lower-middle income**	**Upper-middle income**	**High-income**	**TOTAL Global**
Diabetics with stroke	199868	92186	84804	1353233	1588023	24339	4892	408006	817219	3281914	**4512031**
Diabetics with coronary heart disease	140166	82236	45652	1030849	434656	10938	2067	287639	305636	2061614	**2656956**
Diabetics with stroke and coronary heart disease	90·39	100·62	40·29	1238·83	715·68	4·58	0·75	257·43	336·57	2376·27	**2971**
**TOTAL - reference**	**340124**	**174522**	**130497**	**2385322**	**2023395**	**35282**	**6960**	**695902**	**1123192**	**5345904**	**7171958**
